# Transcriptomic Profiling of Electroacupuncture Regulating the Molecular Network in Hippocampus of Rats with Cerebral Ischemia-Reperfusion Injury

**DOI:** 10.1155/2022/6053106

**Published:** 2022-09-02

**Authors:** Jiang Pan, Hong-Wei Shen, Kai-Lin Yang, Cheng Chen, Wen-Ying Shi, Bi-Dan Lou, Sai-Qun Li, Jin-Wen Ge, Wei Zhang

**Affiliations:** ^1^Department of Acupuncture, The First Affiliated Hospital of Hunan University of Chinese Medicine, Changsha City, Hunan Province, China; ^2^Hunan University of Chinese Medicine, Changsha, Hunan, China; ^3^Department of Medical Experimental Research Center, The Second Xiangya Hospital of Central South University, Changsha City, Hunan Province, China; ^4^Department of Acupuncture, Changsha Central Hospital, Changsha City, Hunan Province, China

## Abstract

**Objective:**

To explore the mechanism of electroacupuncture stimulation of the hand-taiyin meridian in regulating the molecular network of rats with cerebral ischemia-reperfusion injury based on transcriptomics.

**Methods:**

Male SD rats were randomly divided into sham operation group, model group, and electroacupuncture (EA) group. Middle cerebral artery embolization/reperfusion injury (MCAO/R) was used to establish the model group and EA group. The sham operation group only performed sham operation without modeling and any intervention, and the model group was bound daily. The EA group received electroacupuncture to stimulate the acupoints of hand-taiyin meridian for 14 days. Then, neurological scores, pathomorphological observations, and Tunel staining were performed. Finally, the affected hippocampus of the rat was used for transcriptome sequencing and RT-PCR detection.

**Results:**

After electroacupuncture intervention in rats, neurological function scores were improved, and neuronal apoptosis was reduced. The results of transcriptomics showed that a total of 1097 differentially expressed genes were obtained, of which 422 were upregulated and 675 were downregulated. The bioinformatics analysis showed that those differentially expressed genes were related to axon development, neuron projection development, neuron projection morphogenesis, plasma membrane cell projection morphogenesis, cell part morphogenesis, notch signaling pathway, long-term potentiation, MAPK signaling pathway, Hedgehog signaling pathway, and so on. The results of RT-PCR showed that Caspase 9 mRNA increased and BDNF, Grin2a, and PlexinD1 mRNA decreased after electroacupuncture intervention (*P* < 0.05).

**Conclusion:**

Electroacupuncture intervention on hand-taiyin meridian may reduce neurological function scores, inhibit neuron apoptosis, and enhance neuronal repair neuroreparation in MCAO/R rats, which may be related to the regulation of genes such as Caspase 9, BDNF, Grin2a, and PlexinD1.

## 1. Introduction

Stroke is the second leading cause of death in the world and the leading cause of death in China [1]. With the increase of aging population, it brings a great burden to society [2, 3]. The cerebral infarct area can be divided into the infarct core area and the ischemic penumbra, and the ischemic penumbra evolves into the irreversible infarct core area over time. Early opening of occluded cerebral blood vessels to restore cerebral blood flow and save neurons in the ischemic penumbra is the key to the treatment of ischemic stroke [4, 5]. At present, the main clinical treatment strategies for acute ischemic stroke are tissue plasminogen activator thrombolysis and mechanical thrombectomy [6, 7]. However, the prognosis of acute ischemic stroke is still unsatisfactory due to the limitation of the treatment time window, the possible reperfusion injury caused by the restoration of blood perfusion, and the lag of cerebral protection therapy [8]. Therefore, it is necessary to pay attention to brain protection therapy to provide the possibility to save more damaged neurons.

The basic pathophysiological feature of ischemic stroke is neuronal death, which is not only due to ischemia directly but also secondary to ischemia/reperfusion injury, such as: oxidative stress, excitatory amino acid toxicity, calcium overload, inflammatory cascade, etc. [2, 9]. Among them, excessive inflammatory response is the main culprit leading to secondary damage to the ischemic penumbra. After interruption of cerebral blood flow, the supply of oxygen and glucose decreases, leading to an imbalance in cellular ion homeostasis and depolarization of neurons and glial cells [10]. Voltage-dependent calcium ion channels are activated, causing a large influx of calcium ions into cells and promoting the increased expression of reactive oxygen species (ROS) [11]. Neuronal depolarization promotes the release of the excitatory neurotransmitter glutamate and aggravates intracellular calcium overload. When occluded vessels are reperfused, ROS are produced explosively with increased oxygen content and infiltration of inflammatory cells [12]. This stimulates ischemic cells including ischemic neurons to secrete pro-inflammatory factors and chemokines; further activates microglia, astrocytes, peripheral blood leukocytes, endothelial cells, etc.; and promotes the migration of these inflammatory cells, aggregates, secretes a large number of pro-inflammatory factors, and produces an inflammatory amplification effect, which promotes inflammation in damaged areas of brain tissue [9, 13, 14]. Focal neuroinflammation disrupts the blood–brain barrier and aggravates brain injury by enhancing excitotoxicity, cytolysis, oxidative stress, and thrombotic inflammatory responses [15]. The clinical manifestations are cerebral hemorrhagic transformation, cerebral edema, and neurological deterioration. It can be seen that the excessively activated inflammatory response is an important pathological link that causes the secondary injury of brain tissue after reperfusion. In vitro and in vivo models have demonstrated that inhibition of inflammatory responses can reduce infarct volume and improve neurological deficits. However, no satisfactory efficacy has been achieved in clinical trials [16].

Acupuncture as a form of complementary medicine has been widely used around the world [17]. Electro-acupuncture is the product of modern scientific and technological progress. It is a combination of traditional acupuncture and electrical stimulation. It not only inherits the advantages of traditional acupuncture but also has the physiological effect of electrical stimulation. Electroacupuncture has the advantages of less side effects and controllable treatment intensity, frequency, and duration, which is beneficial to clinical treatment and research [18]. At present, electroacupuncture has been used to treat various diseases, such as stroke, arthritis, pain, depression, inflammatory bowel disease, etc. [19] More and more evidences support the role of electroacupuncture in maintaining body homeostasis and immune regulation, which may be the basis for electroacupuncture in the treatment of various inflammatory-related diseases [19–21]. Electroacupuncture with neuroprotective effect has achieved good therapeutic effect in the treatment of ischemic stroke patients and animal models, and the effect of electroacupuncture is closely related to its regulation of various pathological processes of cerebral infarction [22–24]. For example, in a rat model of cerebral ischemia/reperfusion, electroacupuncture can inhibit inflammation and oxidative stress, reduce the activity of microglia, and promote nerve regeneration, thereby promoting the recovery of motor function [25–27]. Our previous studies have shown that acupuncture can effectively improve the Cerebral Blood Flow (CBF) in the infarcted area, para-infarcted area, and mirror area, which provides a partial visual basis for clinical selection of acupoints on the heart meridian to treat ischemic cerebrovascular disease [28,29]. Therefore, this study would establish a middle cerebral artery embolism/reperfusion (MCAO/R) rat model and explore the molecular network regulation mechanism of acupuncture-intervention in MCAO/R rats through transcriptomics.

## 2. Materials and Methods

### 2.1. Experimental Materials

#### 2.1.1. Reagents and Instruments

mRNA reverse transcription kit (Beijing Kangwei Century Biotechnology Co., Ltd., China, CW2569). Huatuo brand filiform needle 0.30 mm × 15 mm, Huatuo electroacupuncture therapeutic apparatus (SDZ-II) (Suzhou Medical Products Factory Co., Ltd., SDZ-II). Automatic enzyme labeling plate washer (PW-812), multi-function enzyme labeling analyzer (MB-530) (Shenzhen Huisong Technology Development Co., Ltd.). Fluorescence quantitative RCP instrument (Thermo, PIKOREAL96, USA). Electrophoresis apparatus (Beijing Liuyi Company, China, DYY-6C).

#### 2.1.2. Experimental Animal

12-week-old SPF-grade healthy male SD rats, weighing 280–300 g, were purchased from Hunan Slike Jingda Laboratory Animal Co., Ltd. (animal license number: SYXK (Xiang) 2018–0003, animal quality certificate number: 0017970). The experimental protocol was approved by the Animal Ethics Committee of Hunan University of Chinese Medicine; animal experiments were in accordance with the National Institute of Health's Guide for the Care and Use of Laboratory Animals. The rats were reared in an environment with constant temperature (22°C), constant humidity (60–70%), and a 12-h day cycle.

### 2.2. Experimental Methods

#### 2.2.1. Establishment of Focal Cerebral Ischemia/Reperfusion Injury Model (MCAO/R)

The MCAO/R model is constructed according to reference [30]. The rats were anesthetized by intraperitoneal injection of sodium pentobarbital (30 mg/kg) and fixed on the operating table. The neck of the rat was sterilized with 75% alcohol, the neck was opened in the middle, and the right common carotid artery, internal carotid artery, and external carotid artery were isolated. A nylon surgical thread (18–22 mm) was then inserted into the left internal carotid artery for 120 min. Then, the insertion wire is pulled out to complete the blood reperfusion process in the ischemic area. In the sham operation group, the operation was the same as above except that the left internal carotid artery was not temporarily occluded. After the operation, the rat was placed on an electric blanket to keep warm and resuscitated, and the Langa score was performed after the rat was fully awake [30]. Scores 1–3 represent successful modeling and are included in subsequent experimental studies.

#### 2.2.2. Grouping and Electroacupuncture

**Figure 1:**
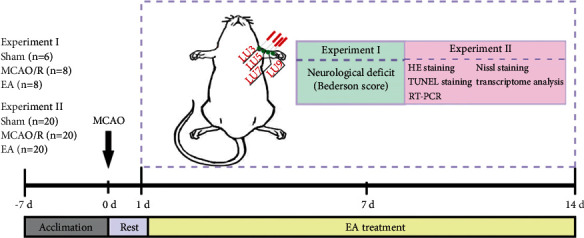
The experimental procedure.

In this study, grouping was performed before modeling and intervention. The rats were randomly divided into a sham operation group (Sham group) (*n* = 26) and modeling group with reference to the random number table method after digital marking on the tail of the rat. After the MCAO/R model of the modeling group was successful, the modeling group was randomly divided into model group (*n* = 28) and electroacupuncture group (EA group) (*n* = 28) according to the random number table method. In the electroacupuncture group, stainless needles were used for electroacupuncture intervention, and all needles were inserted into the depth of 2–3 mm at Tianfu acupoint, Chize acupoint, Kongzui acupoint, and Taiyuan acupoint (The representative of the Hand Tai-yin meridian). After the rats were bundled, 15 mm, 36-gauge filigree needles were used to pierce the acupoints on the affected side, and then the electroacupuncture therapeutic apparatus was connected. After connecting the needle, the polarity is fixed, the distal end is negative, and the proximal end is positive. The parameters of electroacupuncture stimulation were: continuous wave, 20 Hz, voltage 2–4 V, current 4–6 mA, stimulation time 30 minutes. Rats in the model group and the sham-operated group were only bundled with the same degree and duration during the intervention, without electroacupuncture intervention. Electroacupuncture was performed 1 day after MCAO/R for 14 consecutive days, once a day. The experimental procedure is shown in Figure 1.

#### 2.2.3. Neurological Score

Neurological function of the rat model was assessed using the Bederson score on days 1, 7, and 14 of electroacupuncture treatment. The evaluation criteria of the Bederson method are: 0 = no detectable defects; 1 = Rotation of trunk and contralateral forelimb when lifted by tail; 2 = Rat turns in circles to the contralateral side when the rat is fixed with the tail on a flat surface, but the posture is normal at rest; 3 = Reduced resistance to push on the opposite side, leaning to the opposite side at rest, turning significantly to the left; 4 = No spontaneous walking and low level of consciousness [31].

#### 2.2.4. Pathological Observation

The brain tissue was fixed with 4% paraformaldehyde, dehydrated with gradient ethanol under vacuum in xylene, and then embedded in paraffin. The brain tissue was then sectioned coronally (5 *μ*m), followed by Nissl staining and HE staining. Six different fields of view were randomly selected from each group of slices under an optical microscope (×400), and the images were processed by Image-Pro 6.2 software to observe the number of intact neurons in the ischemic cerebral cortex.

#### 2.2.5. Terminal Deoxynucleotidyl Transferase-Mediated Nick End Labeling (TUNEL) Staining

Apoptotic cells in affected cerebral hemisphere were detected according to the method of TUNEL kit. Brain tissue sections deparaffinized in xylene were soaked in graded ethanol (100, 95, 90, 80, 70%) and then treated with proteinase K solution at 37°C for 15–30 min. After washing with phosphate buffered saline (PBS) buffer for 5 min, the sections were incubated with 50 *μ*l terminal transferase (Tdt) and 450 *μ*l horseradish peroxidase (HRP)-dUTP in a box for 2 hours at 37°C. After 3 washes with PBS, 50–100 *μ*l of diaminobenzidine (DAB) substrate was added and left at room temperature for 10 minutes. Tissues were then washed and stained with hematoxylin. After dehydration with graded alcohol, after clearing with xylene, the brain tissue was sealed with neutral glue and imaged with a microscope at ×400. Image J was used to perform cell counting and to calculate apoptotic cell rate. Formula: Apoptotic cell rate (%) = number of positive cells/total number of cells.

### 2.3. Transcriptomic Methods

Total RNA was extracted from the CA1 region of the affected hippocampus of 3 rats in sham operation group, 4 rats in MCAO group, and 4 rats in electroacupuncture group by TRIZOL method. Nanodrop 2000 detects the concentration and purity of samples, evaluates RNA integrity by agarose gel electrophoresis, and determines RNA integrity number (RIN). The total amount of RNA is 1 *μ*g, the concentration is greater than or equal to 50 ng/uL, and the 0D260/280 value is between 1.8 and 2.2; then the library was constructed. Samples with RIN score >8 were used for sequencing. Eukaryotic mRNA sequencing is based on the HiSeq sequencing platform to sequence all mRNAs transcribed from a sample. The Illumnina TnuseqTM RNA sample prep Kit method was used to construct the IlluminaPE library for 2 × 150 bp sequencing. After the QuantiFluor dsDNA System is quantified, it is mixed according to the data ratio, and then the bridge PCR amplification was performed on the cBot to generate groups. Transcriptome data were analyzed after quality control. After the data quality control was completed, statistics and quality assessment were performed again. The DESeq2 package in R software was used for differential expression analysis of transcriptomic data. P-adjust <0.05, Log2FC >1 or <−1 were considered differential genes.

### 2.4. Bioinformatics Analysis

The protein–protein interaction (PPI) data were collected from String (https://cn.string-db.org/) with medium confidence >0.4 and the “Organism” was limited to “*Rattus norvegicus*” [32]. Differentially expressed genes were imported into Metascape (https://metascape.org/gp/index.html#/main/step1) for enrichment analysis to obtain Gene Ontology (GO) enrichment results, Kyoto Encyclopedia of Genes and Genomes (KEGG) pathway, and Reactome pathway [33]. Gene Set Enrichment Analysis (GSEA) was performed by GSEA 4.3.0 software (https://www.gsea-msigdb.org/gsea/index.jsp) [34].

### 2.5. Detection of BDNF, Grin2a, Plexin D1, and Caspase 9 mRNA Expression by Real-Time PCR

The RNA extraction of CA1 region of the affected hippocampus was using the TRIzol Reagent following the instructions. First-strand cDNA was generated from MMLV transcriptase using random primers. Real-time RT-PCR was performed on a CFX96 real-time PCR detection system and gene detection was performed using the Roche SYBR FAST Universal qPCR kit. All primers were purchased from GeneCopoeia. The thermocycling conditions consisted of an initial denaturation for 2 min at 95°C, followed by 40 cycles of 20 sec at 95°C, 30 sec at 60°C, and 40 sec at 72°C. All reactions were performed in an ABI 7500 System. Actin was used as the reference gene. The relative gene expression was calculated using 2^−ΔΔCt^ method.

### 2.6. Statistical Analysis

SPSS version 19.0 was used for statistical analysis and data are presented as mean ± standard deviation. Analyses were performed using one-way ANOVA followed by Bonferroni correction or unpaired Student's *t*-test. *P* < 0.05 was considered to indicate a statistically significant difference.

## 3. Results

### 3.1. Efficacy of Electroacupuncture

#### 3.1.1. Neurological Score

**Figure 2:**
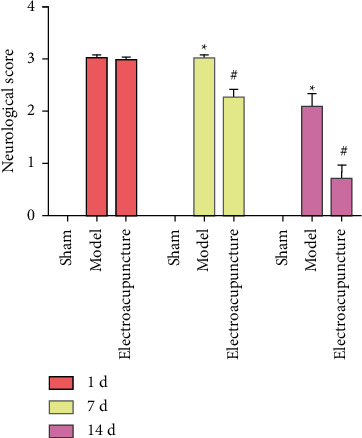
Neurological score (Data are presented as the mean ± SD. ^*∗*^Compared with control group, *P* < 0.05; #compared with model group, *P* < 0.05, *t*-test).

Compared with the sham operation group, the neurological deficit score in the MCAO/R group was increased (*P* < 0.05), indicating that the modeling was successful. Compared with the sham operation group, the neurological function score in the electroacupuncture group was decreased (*P* < 0.05), suggesting that electroacupuncture treatment may improve the neurological function score (Figure 2).

#### 3.1.2. Pathological Changes

**Figure 3:**
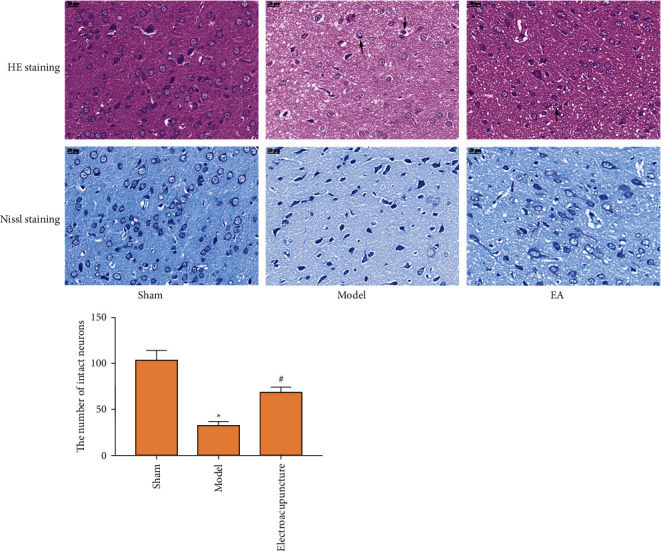
Pathological changes (400×; pathological cells are indicated by black arrows. Data are presented as the mean ± SD. ^*∗*^Compared with control group, *P* < 0.05; #compared with model group, *P* < 0.05, *t*-test).

HE staining showed that compared with the Sham group, the brain tissue of the model group was obviously loose and the number of nerve cells was significantly reduced. Its structure and shape are irregular, the arrangement is unclear, the surrounding swelling is loose, the cytoplasm is lightly stained, and the nucleus is dissolved. Compared with the model group, the pathological improvement of the brain tissue of the EA group was slightly improved on the whole, the number of nerve cells around the infarct was slightly increased, and the structure and shape were more regular. The results of Nissl staining showed that compared with the sham operation group, the Nissl bodies in the model group were significantly reduced. Compared with the model group, the Nissl body in the EA group and the acupuncture group increased significantly (Figure 3).

#### 3.1.3. Neuronal Apoptosis

**Figure 4:**
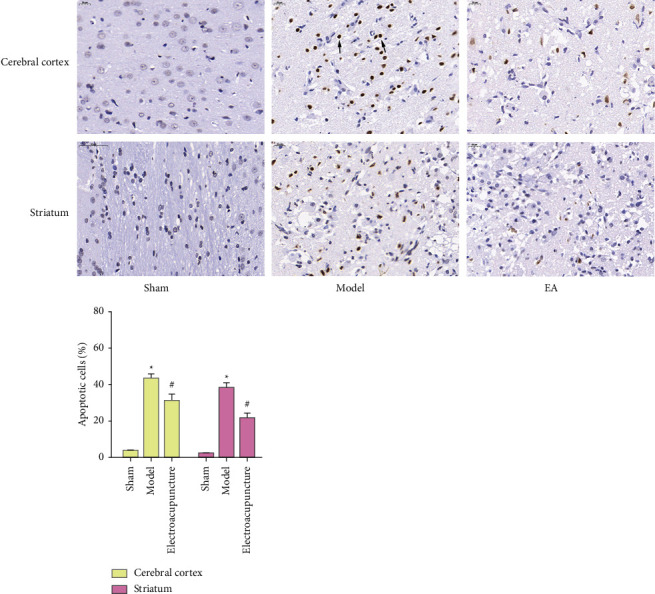
Neuronal apoptosis (400×, Tunel staining. Data are presented as the mean ± SD. *∗*Compared with control group, *P* < 0.05; #compared with model group, *P* < 0.05, *t*-test; pathological cells are indicated by black arrows).

Under light microscopy, the nuclei of apoptotic cells appeared brown (Figure 2). Compared with the sham operation group, the percentage of neuronal apoptosis increased in the MCAO/R group (*P* < 0.05). Compared with the MCAO/R group, the percentage of neuronal apoptosis in the electroacupuncture group was significantly reduced (*P* < 0.05). This suggests that electroacupuncture stimulation can alleviate neurological dysfunction by reducing neuronal apoptosis (Figure 4).

### 3.2. Differentially Expressed Genes

**Figure 5:**
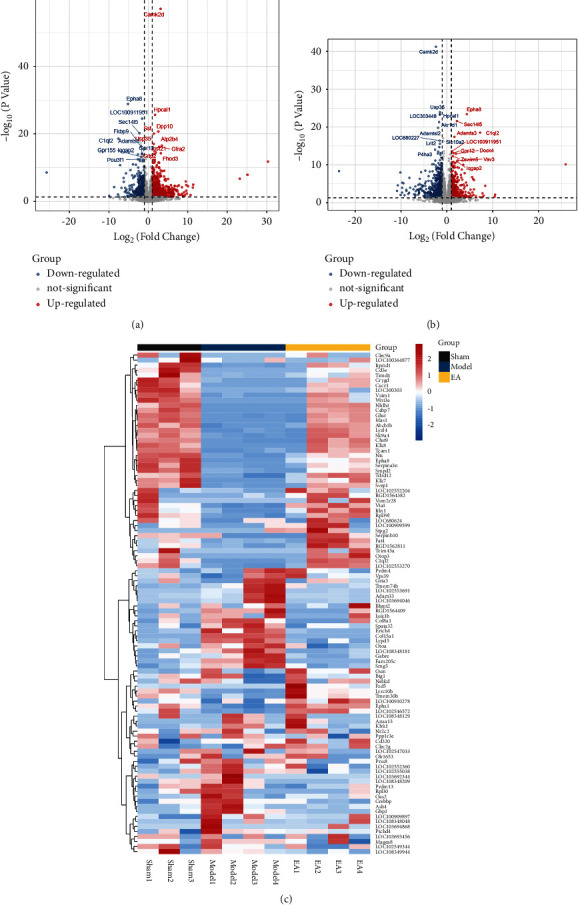
Differentially expressed genes. (a) Volcano plot of model/sham group. (b) Volcano plot of EA/model group. (c) Heatmap of top 50 genes.

**Figure 6:**
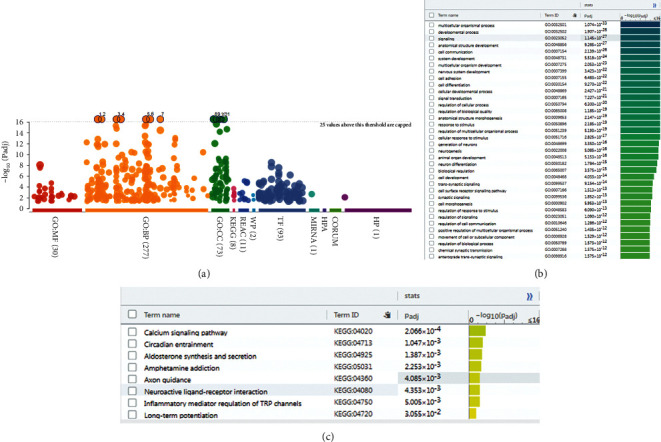
The results of preliminary enrichment of model/sham group. (a) Overview map. (b) Biological processes. (c) Signaling pathways.

Comparing the sham operation group with the model group (Model/Sham group), a total of 1128 differentially expressed genes were obtained, of which 713 were upregulated and 415 were downregulated (Figure 5) (see Table S1). The results of preliminary enrichment showed that the biological processes involved after MCAO/R include regulation of ion transport, cell morphogenesis, regulation of ion transmembrane transport, regulation of transmembrane transport, cell junction organization, synaptic signaling, neuron projection development, cellular component morphogenesis, neuron projection morphogenesis, plasma membrane bounded cell projection morphogenesis, trans-synaptic signaling, cell projection morphogenesis, regulation of system process, and cell morphogenesis involved in differentiation. The pathways involved Neuroactive ligand-receptor interaction, Calcium signaling pathway, Axon guidance, Circadian entrainment, Aldosterone synthesis and secretion, cAMP signaling pathway, Amphetamine addiction, Inflammatory mediator regulation of TRP channels, ECM-receptor interaction, Long-term potentiation, Transcriptional misregulation in cancer, Glutamatergic synapse, Long-term depression, Adrenergic signaling in cardiomyocytes, and Wnt signaling pathway (Figure 6 and Table S2).

**Figure 7:**
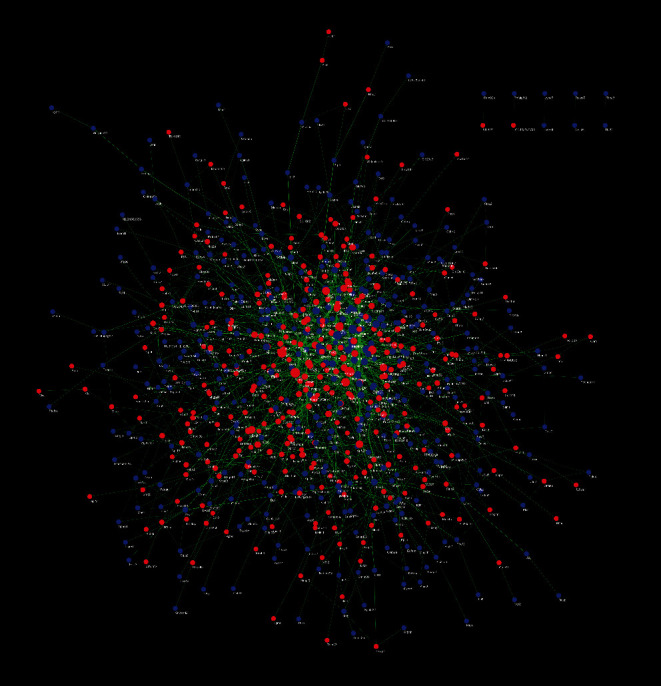
PPI network of differentially expressed genes (red circles stand for upregulated genes; blue circles stand for downregulated genes).

Comparing the model group with the EA group (EA/Model group), a total of 1097 differentially expressed genes were obtained, of which 422 were upregulated and 675 were downregulated (Figure 5) (see Table S3). The upregulated and downregulated genes were input into Cytoscape to construct network (Figure 7). The top 10 upregulated genes were Ptk2b (47 edges), Wnt2 (46 edges), Crebbp (40 edges), Erbb3 (36 edges), Gnb4 (34 edges), Grin2a (31 edges), Gria1 (30 edges), Kit (29 edges), Ldb3 (26 edges), and Fa2h (26 edges); the top 10 downregulated genes were Alb (63 edges), Igf1 (46 edges), Gnb3 (37 edges), Mapk11 (37 edges), Stat4 (32 edges), Plcg2 (31 edges), Mapk13 (27 edges), Mef2c (27 edges), Mapk12 (27 edges), and Cd40 (25 edges).

### 3.3. Bioinformatics Analysis for Differentially Expressed Genes of EA/Model Group

#### 3.3.1. Upregulated Gene Analysis

**Figure 8:**
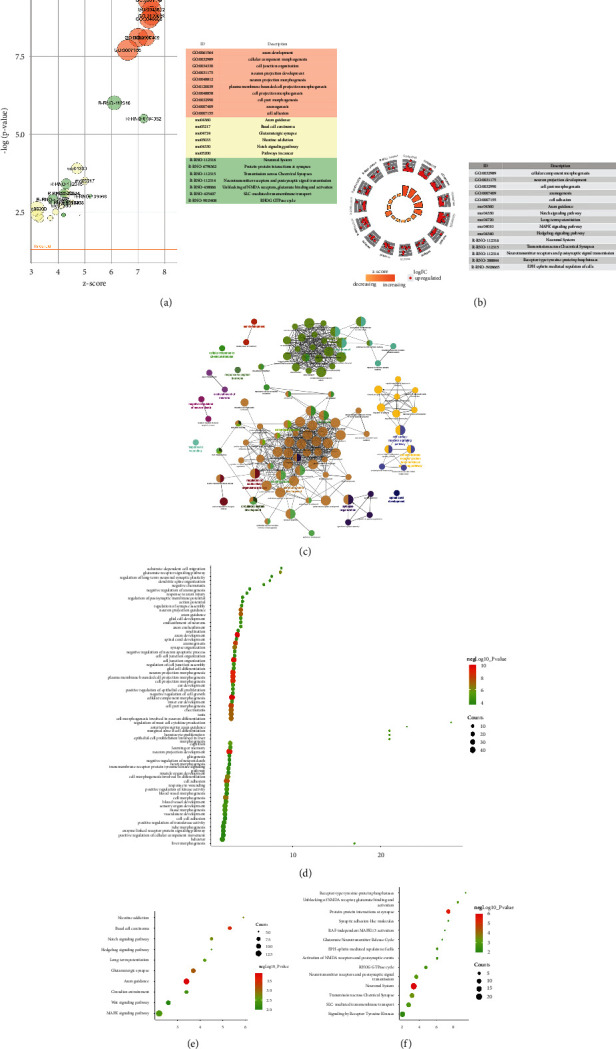
Upregulated gene analysis. (a) Top 10 results of each category. (b) Gene expression profiles of the top 5 enrichment results; (c) enrichment results profile. (d) bubble chart of biological processes; (e) bubble chart of signaling pathways; (f) bubble chat of reactome pathways; *X*-axis stands for enrichment value.

The upregulated genes were input into Metascape for enrichment analysis. The biological processes include axon development, cellular component morphogenesis, cell junction organization, neuron projection development, neuron projection morphogenesis, plasma membrane bounded cell projection morphogenesis, cell projection morphogenesis, cell part morphogenesis, axonogenesis, cell adhesion, axon guidance, neuron projection guidance, chemotaxis, and so on. The signaling pathway include Axon guidance, Basal cell carcinoma, Glutamatergic synapse, Nicotine addiction, Notch signaling pathway, Long-term potentiation, MAPK signaling pathway, Hedgehog signaling pathway, Circadian entrainment, and Wnt signaling pathway. The Reactome pathway include Neuronal System, Protein–protein interactions at synapses, Transmission across Chemical Synapses, Neurotransmitter receptors and postsynaptic signal transmission, Unblocking of NMDA receptors, glutamate binding and activation, SLC-mediated transmembrane transport, RHOG GTPase cycle, Receptor-type tyrosine-protein phosphatases, EPH-ephrin-mediated repulsion of cells, Activation of NMDA receptors and postsynaptic events, Signaling by Receptor Tyrosine Kinases, Synaptic adhesion-like molecules, RAF-independent MAPK1/3 activation, and Glutamate Neurotransmitter Release Cycle (Figure 8 and Table S4).

#### 3.3.2. Downregulated Gene Analysis

**Figure 9:**
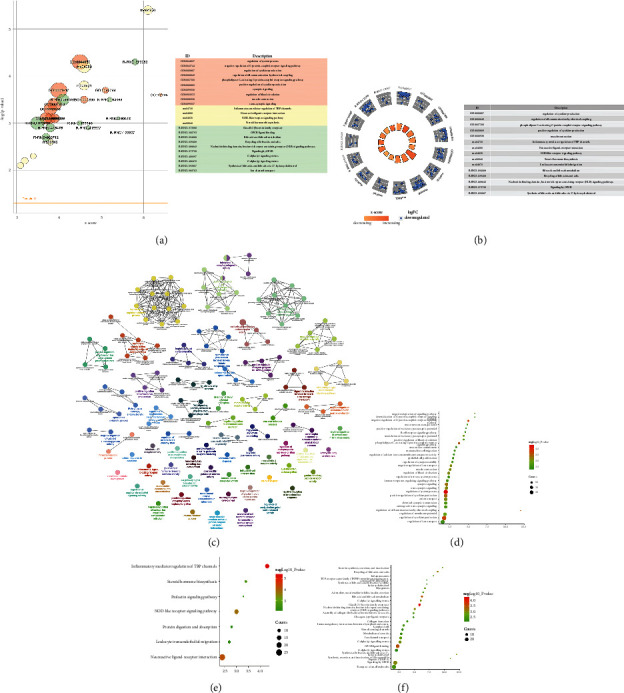
Downregulated gene analysis. (a) Top 10 results of each category. (b) Gene expression profiles of the top 5 enrichment results. (c) Enrichment results profile. (d) Bubble chart of biological processes. (e) Bubble chart of signaling pathways; (f) Bubble chat of reactome pathways; *X*-axis stands for enrichment value.

The downregulated genes were input into Metascape for enrichment analysis. The biological processes include regulation of system process, negative regulation of G protein-coupled receptor signaling pathway, regulation of cytokine production, regulation of cell communication by electrical coupling, phospholipase C-activating G protein-coupled receptor signaling pathway, positive regulation of cytokine production, synaptic signaling, regulation of blood circulation, muscle contraction, trans-synaptic signaling, negative regulation of ion transport, anion transport, modulation of excitatory postsynaptic potential, regulation of membrane potential, chemical synaptic transmission, and so on. The signaling pathway includes Inflammatory mediator regulation of TRP channels, Neuroactive ligand-receptor interaction, NOD-like receptor signaling pathway, Steroid hormone biosynthesis, Prolactin signaling pathway, Leukocyte transendothelial migration, and Protein digestion and absorption. The Reactome pathway includes Class B/2 (Secretin family receptors), GPCR ligand binding, Bile acid and bile salt metabolism, Recycling of bile acids and salts, Nucleotide-binding domain, Leucine-rich repeat containing receptor (NLR) signaling pathways, Signaling by GPCR, G alpha (*z*) signaling events, G alpha (*q*) signaling events, Synthesis of bile acids and bile salts via 27-hydroxycholesterol, Ion channel transport, Synthesis, secretion, and inactivation of Glucagon-like Peptide-1 (GLP-1), Myogenesis, Immunoregulatory interactions between a Lymphoid and a nonlymphoid cell, Adrenaline, noradrenaline inhibits insulin secretion, Incretin synthesis, secretion, inactivation, and so on (Figure 9, Table S5).

#### 3.3.3. All Gene Analysis

**Figure 10:**
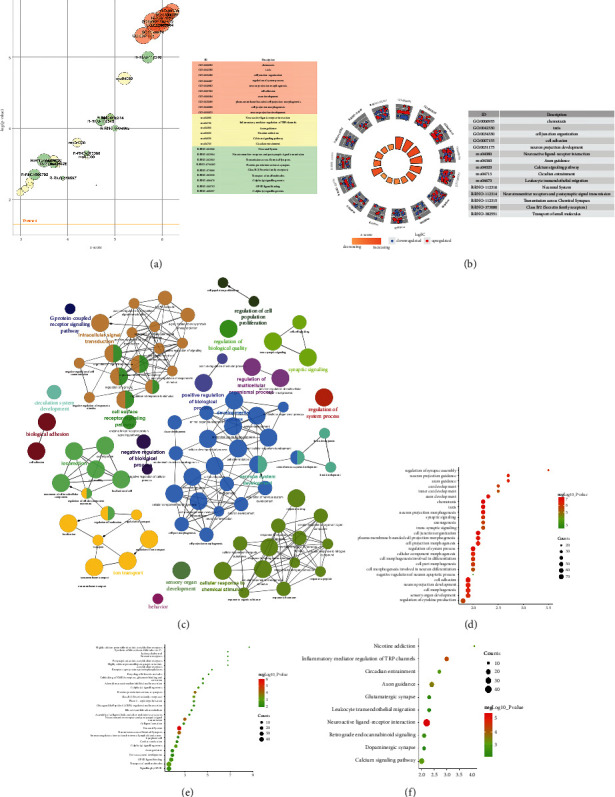
All gene analysis. (a) Top 10 results of each category. (b) Gene expression profiles of the top 5 enrichment results; (c) enrichment results profile. (d) Bubble chart of biological processes; (e) bubble chart of signaling pathways; (f) bubble chat of reactome pathways; *X*-axis stands for enrichment value.

The up- and down-regulated genes were input into Metascape for enrichment analysis. The biological processes include chemotaxis, taxis, cell junction organization, regulation of system process, neuron projection morphogenesis, cell adhesion, axon development, plasma membrane bounded cell projection morphogenesis, cell projection morphogenesis, neuron projection development, axon guidance, cell morphogenesis, neuron projection guidance, cellular component morphogenesis, and so on. The signaling pathway include Neuroactive ligand-receptor interaction, Inflammatory mediator regulation of TRP channels, Axon guidance, Nicotine addiction, Calcium signaling pathway, Circadian entrainment, Retrograde endocannabinoid signaling, Glutamatergic synapse, Leukocyte transendothelial migration, Dopaminergic synapse. The Reactome pathway includes Neuronal System, Neurotransmitter receptors and postsynaptic signal transmission, Transmission across Chemical Synapses, Protein–protein interactions at synapses, Class B/2 (Secretin family receptors), Transport of small molecules, G alpha (*q*) signaling events, GPCR ligand binding, G alpha (*z*) signaling events, Highly calcium permeable nicotinic acetylcholine receptors, Adrenaline, noradrenaline inhibits insulin secretion, Unblocking of NMDA receptors, glutamate binding and activation, Signaling by GPCR, Phase 0–rapid depolarization, Receptor-type tyrosine-protein phosphatases, and so on (Figure 10, Table S6).

### 3.4. Gene GSEA Results for Differentially Expressed Genes of EA/Model Group

**Figure 11:**
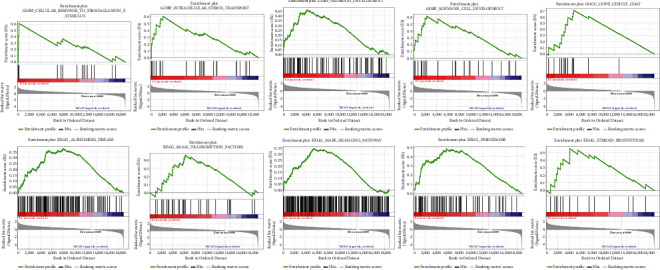
The top 5 GO results and signaling pathways in GSEA (upregulated genes).

**Figure 12:**
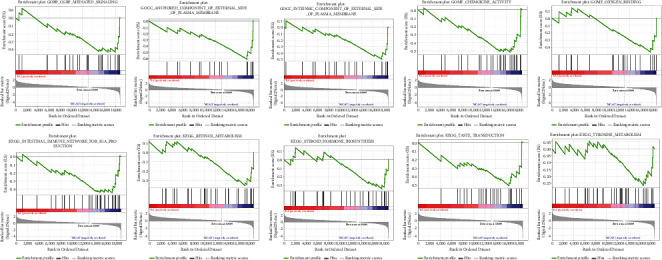
The top 5 GO results and signaling pathways in GSEA (downregulated genes).

The GSEA results showed that the upregulated genes were mainly concentrated in GO results such as Schwann Cell Development, Cellular Response To Prostaglandin E Stimulus, Intracellular Sterol Transport, Midbrain Development, CopII Vesicle Coat, Inclusion Body Assembly, Multivesicular Body Sorting Pathway, Neurotransmitter Receptor Internalization, Negative Regulation Of Endocytosis, Autophagosome Organization, and so on; and the downregulated genes were mainly concentrated in Anchored Component Of External Side Of Plasma Membrane, Oxygen Binding, Intrinsic Component Of External Side Of Plasma Membrane, Cgmp-Mediated Signaling, Chemokine Activity, Nitric Oxide–Mediated Signal Transduction, Neuropeptide Hormone Activity, CCR Chemokine Receptor Binding, Chemokine Receptor Binding, Synaptic Transmission Cholinergic, and so on. The upregulated genes were mainly concentrated in signaling pathways such as Basal Transcription Factors, Peroxisome, Alzheimer's Disease, Steroid Biosynthesis, Mapk Signaling Pathway, ERBB Signaling Pathway, Valine Leucine And Isoleucine Degradation, Regulation Of Autophagy, Wnt Signaling Pathway, Adipocytokine Signaling Pathway and so on; and the downregulated genes were mainly concentrated in signaling pathways such as Taste Transduction, Intestinal Immune Network For Iga Production, Steroid Hormone Biosynthesis, Retinol Metabolism, Tyrosine Metabolism, Neuroactive Ligand Receptor Interaction, Cytokine Cytokine Receptor Interaction, Nod-Like Receptor Signaling Pathway, Pentose And Glucuronate Interconversions, Complement And Coagulation Cascades. The top 5 results were shown in Figures 11 and 12.

### 3.5. Hub Genes Expression Validation

**Figure 13:**
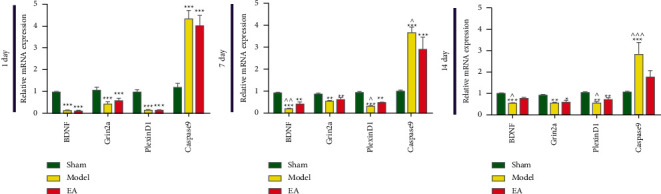
Validation of the expression patterns of four hub genes by RT-qPCR (data are presented as the mean ± SD. Compared with the sham operation group, ^*∗∗∗*^*P* < 0.001, ^*∗∗*^*P* < 0.01 and ^*∗*^*P* < 0.05; compared with EA group,  ^∧∧∧^*P* < 0.001,  ^∧∧^*P* < 0.01 and  ^∧^*P* < 0.05 in the corresponding time, *t*-test).

According to the results of bioinformatics analysis and GSEA, four genes (Caspase9, BDNF, Grin2a, and plexinD1) were selected to validate in ischemic hippocampus. The results of RT-PCR showed compared with the sham operation group, the expression of Caspase 9 mRNA increased, and the expression of BDNF, plexinD1, and Grin2a mRNA decreased in MCAO/R groups. Compared with MCAO/R group, the expression of Caspase9 mRNA decreased, and the expression of BDNF and PlexinD1 mRNA increased in the EA group (Figure 13).

## 4. Discussion

Stroke is a frequently occurring disease among adults in modern society, and it is one of the most common diseases with high disability and high fatality, with a disability rate as high as 33.4%–44.6% [35, 36]. Among them, ischemic stroke is the most important type of stroke in clinical practice, and its incidence accounts for more than 50% of all cerebrovascular diseases [37]. In terms of clinical treatment strategies, reperfusion therapy can improve the clinical symptoms of patients to a certain extent. However, even with the standard therapy of intravenous thrombolytic drugs combined with endovascular thrombectomy, there are still a large number of patients with severe disability, which may be accompanied by complications such as intracranial hemorrhage after thrombolysis. Evidence-based medical studies on the successful endovascular treatment of stroke found that only 18.5%–32.5% of stroke patients could achieve successful reperfusion in time within 3–8 hours after the onset of stroke. Even if patients receive standard medical treatment at an early stage, 50% to 60% of patients still have neuromotor dysfunction of varying degrees [38, 39]. Therefore, it is very important to explore more effective therapies, especially for the improvement of neurological function in the recovery period of cerebral ischemia. Clinical practice and experimental studies have confirmed that acupuncture is safe and effective in treating cerebral ischemic sequelae, and it has been more and more widely used worldwide [40, 41]. Another meta-analysis showed that acupuncture exerted a potential neuroprotective effect in ischemic stroke, reducing infarct volume and improving neurological function scores [42]. Acupuncture on human acupoints, such as Quchi [43] and Baihui [44], can improve neurological function after cerebral ischemia. Traditional Chinese medicine believes that acupuncture at Neiguan acupoint can calm and dredge the meridians. Acupuncture at Neiguan and Dazhui points can significantly reduce the expression of TNF-*α* and NF-*κ*B-p65 in the ischemic hippocampal CA1 region [45], thereby reducing cerebral ischemia injury. Our previous study also found that acupuncture at Neiguan acupoint could improve the recovery of neurological function in rats with middle cerebral artery occlusion [46]. This study found that electroacupuncture could improve neurological function and neuronal apoptosis in MCAO/R model rats.

Cerebral infarction is most likely to cause a large number of free radical formation, calcium overload, excitatory amino acid toxicity, inflammatory mediators, and other related changes in the brain tissue in the blood flow supply area after the interruption of blood flow [47], which further leads to the breakdown of the blood–brain barrier in the brain tissue, resulting in increased blood–brain barrier (BBB) permeability, edema in the brain tissue, and then neuronal cell death [48]. Neurovascular unit (NVU) refers to the basic functional unit composed of neurons, BBB, astrocytes, extracellular matrix, and microvessels [49]. When studying cerebral infarction, taking NVU as a whole can help better understand the pathological mechanism of brain injury process, which is helpful for the treatment of cerebral infarction. In the NVU, microvascular endothelial cells, glial cells, basement membrane, etc., together constitute the BBB [50], and microvessels play an important role in the energy supply of brain cells in brain tissue. BBB plays an important role in maintaining the stability of the internal environment of the nervous system, controlling the normal exchange of ions and water, and maintaining the balance of cerebrospinal fluid [51]. Protecting the BBB in the early stage, ensuring the energy supply of microvessels to brain cells, and reducing the death of neuronal cells play an important role in the treatment of cerebral infarction.

This study explored the molecular network mechanism of electroacupuncture stimulation of hand Tai-yin meridian in the treatment of cerebral ischemia-reperfusion by mRNA high-throughput sequencing analysis. Compared with MCAO/R group, 485 upregulated mRNA and 611 downregulated mRNA were obtained. In the network, the top 10 upregulated genes were Wnt2 (48 edges), Ptk2b (43 edges), Crebbp (42 edges), Kdr (37 edges), Fgfr1 (35 edges), Gria1 (32 edges), Ntrk1 (32 edges), Kit (31 edges), Ldb3 (27 edges), and Pdgfb (26 edges); the top 10 downregulated genes were Alb (58 edges), Igf1 (51 edges), Stat4 (30 edges), Pax6 (29 edges), Mef2c (26 edges), Rhod (25 edges), Sst (25 edges), Mapk13 (23 edges), Rnd1 (23 edges), and Nanog (22 edges). The enrichment analysis results and GSEA results showed that those differentially expressed genes were related to Chemotaxis, Taxis, Cell junction organization, Neuron projection morphogenesis, Cell adhesion, Axon development, Neuroactive ligand–receptor interaction, Inflammatory mediator regulation of TRP channels, Axon guidance, Calcium signaling pathway, Circadian entrainment, Retrograde endocannabinoid signaling, Glutamatergic synapse, Leukocyte transendothelial migration, Dopaminergic synapse, Neuronal System, Neurotransmitter receptors and postsynaptic signal transmission, Transmission across Chemical Synapses, Protein–protein interactions at synapses, and so on. In addition, some biological processes and signaling pathways (such as synaptic Signaling, Neuron projection development, Cellular component morphogenesis, Neuron projection morphogenesis, Neuroactive ligand-receptor interaction, Calcium signaling pathway, Axon guidance, Circadian entrainment, Glutamatergic synapse) in the Model/Sham group can also be found in the EA/Model group, suggesting that these pathways may be the core pathways for EA to treat cerebral ischemia-reperfusion injury.

The results of high-throughput sequencing transcriptomics were further validated by qRTPCR. Among them, brain-derived neurotrophic factor (BDNF) is widely distributed in the central system and is one of the important neurotrophic factors that maintain the survival of neurons in the brain [52]. It is secreted by the pyramidal cells of the cerebral cortex and transported anterogradely to the nerve endings through the neuron cell body, thereby nourishing the distal tissues [53]. When the cortex is damaged, the anterograde transported BDNF decreases and cannot continue to maintain the survival of neurons. It may be one of the main factors of motor sensory pathway damage [53]. However, the study found that the expression of BDNF mRNA in the bilateral cerebral cortex in the early stage of stroke was upregulated, and the mRNA expression in the contralateral cortex was higher than that in the affected side. This suggests that the early changes of BDNF on the contralateral side may be involved in the repair and regeneration of damaged cortical nerves [54, 55]. The present study demonstrates that reactivation of Sema3E-Plexin-D1 signaling after ischemic stroke is critical for the re-establishment of healthy vasculature through modulation of VEGF signaling during vascular remodeling. Among them, the expressions of Sema3E and PlexinD1 in the nervous and vascular system changed significantly after birth [56]. In the developing brain, PlexinD1 is widely detected in capillary endothelial cells. Furthermore, the findings suggest that it is different from the developing brain or peripheral vasculature. In the developing mouse retina, PlexinD1 expression is regulated by VEGF signaling, a major hypoxia-inducible pathway, during retinal angiogenesis [57]. Furthermore, Sema3E/PlexinD1 signaling inhibits postischemic angiogenesis by regulating endothelial DLL4 and filopodia formation in a rat model of ischemic stroke [58]. Yu et al. showed that ischemic injury rapidly induced Sema3e expression in neurons in the peri-infarct region, followed by PlexinD1 upregulation in remodeling vessels [59]. Interestingly, the re-emergence of PlexinD1 coincides with the entry of cerebral blood vessels into an active angiogenic process. Consistent with this, Plxnd1 ablation worsened neurological deficit, infarct volume, neuronal survival, and blood flow recovery. Furthermore, decreased and abnormal vascular morphogenesis results from abnormally increased VEGF signaling. Significant extravasation of intravenous tracers in the brain parenchyma, downregulation of connexins, and mislocalization in regenerated vessels were observed in Plxnd1 knockout mice. This suggests that loss of Sema3E-Plexin-D1 signaling is associated with BBB damage. Inhibition of VEGF signaling during vascular remodeling restores abnormal behavioral manifestations, abnormal vascular phenotypes, and defects in BBB disassembly in Plxnd1 knockout mice. These findings suggest that Sema3E-Plexin-D1 signaling can promote functional recovery by downregulating VEGF signaling in the injured adult brain [59]. Thus, the current study shows that PlexinD1 expression is essential in critical situations, such as ischemia-induced vascular remodeling, in which newly sprouted vessels require vascular guidance in response to VEGF signaling [60].

Studies of acupuncture in the treatment of cerebral infarction have also shown that it has an important effect in inhibiting inflammation. Song et al. performed acupuncture at Baihui acupoint and Zusanli acupoint on the ipsilateral side of cerebral ischemia-reperfusion model rats, and found that both IL-1*β* and ICAM-1 in the brain region of the healthy side of the rats showed a trend of increasing, which was statistically different from that of the model group [61]. It can be seen that acupuncture can upregulate the expression of related inflammatory factors in the brain region, inhibit the inflammatory response, and play a role in brain protection. Wang et al. found that the local blood flow of the healthy side of the rat showed a short-term increase trend after electroacupuncture at the Renzhong point of the middle cerebral artery embolism model rat, which shows that electroacupuncture treatment can increase the blood flow compensation of the healthy side to the injured side [62]. Huang et al. used the giant needling method to acupuncture the contralateral limbs of rats with focal ischemia, and found that the giant needling method could improve the neurological function of MCAO rats and reduce the infarct size [63]. He treated the patients with acute ischemic cerebral infarction with giant needling, the balance of the affected limbs of the patients was improved, and the neurological damage was alleviated. Therefore, acupuncture has a good effect on cerebral infarction at present [64].

## 5. Conclusion

In this study, the biological mechanisms of neuroplasticity and angiogenesis of electroacupuncture in the treatment of cerebral ischemia-reperfusion injury were preliminarily explored based on the transcriptomic strategy. In the future, we will further explore the signaling pathways and molecular mechanisms related to neuroplasticity in combination with other omics. Based on current evidence, electroacupuncture intervention on hand-taiyin meridian can improve neurological function scores, neuron apoptosis, and neuroreparation in MCAO/R rats, which may be related to the regulation of genes such as Caspase 9, BDNF, Grin2a, and PlexinD1.

## Data Availability

All data generated or analyzed during this study are included in this published article.

## Supplementary Materials

Supplementary Materials Table S1: differentially expressed genes of Model/Sham group; Table S2: preliminary enrichment results of Model/Sham group; Table S3: differentially expressed genes of EA/Model group; Table S4: upregulated gene analysis; Table S5: downregulated gene analysis; Table S6: all gene analysis.
