# Supplementary material for: Transcriptomic Profiling of Electroacupuncture Regulating the Molecular Network in Hippocampus of Rats with Cerebral Ischemia-Reperfusion Injury
Source: Evid Based Complement Alternat Med. 2022 Sep 2;2022:6053106. doi: 10.1155/2022/6053106 (PMC9463016; doi:10.1155/2022/6053106)
Supplement: Supplementary Materials — Table S1: differentially expressed genes of Model/Sham group; Table S2: preliminary enrichment results of Model/Sham group; Table S3: differentially expressed genes of EA/Model group; Table S4: upregulated gene analysis; Table S5: downregulated gene analysis; Table S6: all gene analysis. [file 6053106.f1.zip › Table S1 (1).pdf]

Table S1 Differentially expressed genes of Model/Sham group

Gene	log2FoldChange	FDR
Trim37	-25.80151494	2.73E-09
Vps39	-9.943400223	0.025716
Klk8	-8.672668624	6.07E-05
Smpd2	-7.708118763	0.009242
C1ql2	-7.609739414	2.74E-19
Smg5	-7.490049024	0.044929
Cd3e	-7.16520388	1.86E-11
Nhlh1	-6.799021898	0.000106
Serpina3n	-6.272620566	1.23E-07
Cxcr1	-6.224837903	1.67E-06
LOC102546572	-6.020191643	0.049475
Wnt3a	-5.965936216	0.001536
Klk7	-5.884307904	0.000105
Itprid1	-5.68443659	0.000851
Timd4	-5.673123093	4.98E-09
Otop3	-5.527115367	0.000332
Vstm1	-5.465417324	0.003248
Gria3	-5.283080488	0.02391
Btg1	-5.257563869	0.038785
LOC300303	-5.173128656	0.03026
Epha8	-5.164879928	1.45E-29
Dmrt3	-5.015023812	0.002769
Chst9	-4.763948163	2.62E-05
LOC688459	-4.732358859	0.000172
Ghsr	-4.710011036	0.000465
Cyp2e1	-4.693206439	0.007266
Crygd	-4.59241223	0.028712
Svep1	-4.536407306	5.43E-10
Nts	-4.283238458	0.011251
Pga5	-4.279436473	0.017137
LOC102553270	-4.250382353	1.57E-05
Abcb1b	-4.148096975	6.58E-05
Cabp7	-4.114919921	1.93E-08
Clec9a	-4.075885197	0.032465
Mas1	-4.051540939	0.000359
Ttr	-3.913532559	0.036714
Prox1	-3.904615894	1.07E-11
Slc9a4	-3.865289642	0.001315
Ppl	-3.836279599	1.24E-07
Tcam1	-3.795289668	0.001389
Crebbp	-3.79245919	0.042189
Sostdc1	-3.762982621	0.007504
Lyzl4	-3.683743377	1.10E-05
Shisa6	-3.610196111	0.002255
Fibcd1	-3.56164592	5.45E-08
LOC102555817	-3.554128285	0.02667
Btbd16	-3.465476736	0.000929
Htr4	-3.412478471	0.001386
Ccdc27	-3.394835381	0.00536
Lrrc10b	-3.383738696	2.70E-08
RGD1562638	-3.380067885	0.004312
RGD1562811	-3.326621236	0.044809
Col6a5	-3.223386731	0.001994
Kcp	-3.162709308	1.33E-11

Fat4	-3.144199804	0.000929
Rpl39l	-3.121057975	0.014749
Grin2a	-3.11414431	0.001841
Htr1a	-3.056570953	0.034227
Krt27	-3.038011795	0.002053
Lhx9	-3.036504985	0.000285
Meox2	-3.024856057	0.000223
Cldn1	-2.990700543	0.0004
Twist1	-2.960877745	0.001139
Klk10	-2.950357135	0.006718
Pax2	-2.933238603	0.027794
Hdc	-2.831137442	0.033618
LOC108348049	-2.819224022	6.85E-09
Piezo2	-2.795356448	0.000847
Mmp23	-2.779399289	0.000115
Kcnj13	-2.76275411	0.018868
Ddit4l	-2.762566944	0.000791
Fgfr4	-2.750393728	0.007768
Arg1	-2.68020936	0.003581
NEWGENE_620180	-2.679398017	0.011653
Vav3	-2.645764344	3.50E-13
Slc2a9	-2.641528356	0.000297
Slc9a2	-2.640637071	0.002559
Icam5	-2.633377085	3.68E-08
Iqgap2	-2.625507521	1.16E-14
Pcsk1	-2.612020594	0.018355
Tanc1	-2.604343378	1.67E-06
Wnt2	-2.594879473	0.001667
Gstm6	-2.580478972	2.86E-07
Bhlhe22	-2.574857709	0.004981
Ntrk1	-2.563898171	0.037679
Itgbl1	-2.56324892	0.00057
Hpca	-2.556465953	9.30E-11
Stac	-2.543625164	0.037465
Atp2a1	-2.537315461	0.010411
Lingo3	-2.509171857	6.36E-05
Neurog2	-2.501127737	0.044798
Nr3c2	-2.494677947	4.21E-07
Ccdc85a	-2.493560883	0.000208
Wipf3	-2.480635381	1.14E-09
Neurod6	-2.479140595	0.039702
Lpl	-2.438175663	3.00E-06
Rem2	-2.433081968	5.47E-05
Dupd1	-2.430400653	0.004694
Alkal2	-2.411048479	0.040641
Clcf1	-2.402713222	3.91E-05
Fbn1	-2.401670028	0.000301
Rerg	-2.396158061	1.09E-06
Kctd4	-2.354180975	0.00043
Calml4	-2.353345975	3.32E-06
LOC102550773	-2.331515117	0.027959
Clmp	-2.331391099	0.002979
Sncg	-2.319944822	6.41E-06
Gpr161	-2.310947618	0.00019
Tspan18	-2.285217317	0.007712
Gjd2	-2.26152754	0.019709

Dpf3	-2.248435054	2.23E-05
Neurod1	-2.246578667	0.019992
Gpr176	-2.242261883	1.11E-07
Sec14l5	-2.242105117	6.70E-21
Gdf10	-2.239414167	0.004477
Tmem54	-2.223445372	4.07E-09
Rgs13	-2.186252288	0.021116
Cd55	-2.173912028	6.43E-07
Dnah9	-2.169330675	5.91E-07
Pkp2	-2.163434079	0.004683
Dgat2	-2.161095451	1.62E-08
Slc16a14	-2.126661436	0.000166
Tle1	-2.124091884	1.08E-12
Il16	-2.10674234	2.35E-08
Cacna1h	-2.10408503	2.39E-07
Pou3f1	-2.093992591	3.30E-13
LOC108349154	-2.086250336	0.000107
Smpdl3b	-2.068384316	3.15E-05
LOC103690059	-2.061285009	0.014766
Kcng2	-2.048396561	0.003447
Itga11	-2.036752121	0.020922
St6galnac5	-2.033400343	1.34E-05
Tuba8	-2.023356684	0.022741
Tmem74	-1.999309375	0.013057
Mpp7	-1.996277716	0.002354
Galnt17	-1.994524854	0.006594
Dusp5	-1.980311532	0.001021
Thpo	-1.979435826	0.028147
C1qtnf6	-1.958555035	0.002241
Ankdd1a	-1.957219428	5.76E-08
Kit	-1.954944891	0.00108
Ror2	-1.9527301	7.20E-05
Nrp1	-1.924528516	0.000388
Smo	-1.913176418	6.30E-05
Tph2	-1.909509082	0.007382
Dsp	-1.908745624	0.000163
Epha7	-1.908561158	0.000306
Pxdn	-1.906606505	0.000166
Gabra5	-1.899361684	0.005118
Sema3e	-1.893375339	7.20E-05
Zfp804a	-1.890996376	3.91E-05
Tnfrsf25	-1.889721632	0.03024
Myom2	-1.887888095	0.00083
Gpc4	-1.885276324	8.01E-05
Tdrd5	-1.88389557	0.029328
Sema5a	-1.875855565	0.000525
Fkbp9	-1.875281729	5.81E-17
Clstn2	-1.867319099	0.004818
Nrip3	-1.852332668	0.020792
Nr4a3	-1.849485995	0.00048
Pwwp2b	-1.846275111	3.31E-09
Tbc1d8b	-1.844025122	4.26E-07
Lyst	-1.843593247	1.30E-07
Plk5	-1.834424771	0.001016
Rsph10b	-1.823572085	0.001685
Scn3b	-1.823420607	1.88E-05

Lrrn1	-1.819598649	4.26E-07
Foxo6	-1.817249637	5.53E-08
Cyp1b1	-1.799655434	0.011108
Cebpb	-1.78861773	7.24E-05
Jph1	-1.785163974	0.025127
Esyt3	-1.782130098	9.47E-08
Kank4	-1.774168756	0.000208
St5	-1.763521958	8.35E-05
Chrna2	-1.762491583	0.000398
Plppr1	-1.762329149	0.00037
St18	-1.762064444	2.71E-06
Tjp3	-1.761341954	0.000514
Cacng8	-1.760866876	7.20E-05
Crlf1	-1.751482746	0.000429
Pxmp4	-1.743715775	0.001259
Galnt18	-1.742727996	3.49E-06
Gpr155	-1.734630485	6.47E-14
Rnf182	-1.717401494	0.011217
Itgb4	-1.713392884	0.005007
Rab38	-1.712848344	0.038902
Hhip	-1.70655917	0.021829
Cdc42bpg	-1.704502615	8.96E-05
Mical1	-1.701709531	0.010177
LOC100361898	-1.694795935	0.017622
Epha6	-1.6929129	0.000511
Rgs10	-1.691789764	0.001209
Slc16a11	-1.680350253	0.000368
Prss55	-1.675421926	0.000152
Stk26	-1.656358485	8.96E-05
Ephb2	-1.649657577	0.020334
Sipa1l3	-1.643218353	6.60E-06
LOC108348064	-1.64039353	7.26E-07
Nipal4	-1.638529359	0.011594
Cebpd	-1.634066116	1.61E-06
Ccdc71l	-1.633763637	0.000344
Homer3	-1.621618539	4.80E-05
Dock10	-1.619184174	1.52E-06
Nr4a2	-1.614036142	0.043517
Cotl1	-1.610296239	0.000711
Perp	-1.610182423	0.032543
Rcn3	-1.609353674	0.000145
Cpne6	-1.608647882	0.00335
Hunk	-1.601505343	0.013656
Arhgef37	-1.600299028	1.05E-05
Dock4	-1.594186011	9.31E-08
Pxylp1	-1.592725553	8.31E-05
Gpr12	-1.592108001	9.17E-15
Adamts3	-1.591617606	6.67E-15
Htr5b	-1.589892936	0.035149
Smpd5	-1.587937837	0.024134
Selenov	-1.582733708	0.01688
Lsm8	-1.582606666	0.000714
Fam155b	-1.581330747	5.11E-05
Pdcl2	-1.57987939	0.041334
Tp53inp1	-1.573027883	0.000474
Clgn	-1.570994543	0.000333

Hs3st4	-1.570942573	0.000182
Pqlc1	-1.568566098	0.001685
LOC100911951	-1.56740629	2.96E-25
Grifin	-1.564387262	0.035016
Cdc40	-1.561258418	4.59E-05
Zbtb18	-1.560696746	0.042452
Kcnip2	-1.559170971	0.002199
Cecr2	-1.555319798	3.00E-06
Dusp6	-1.554658158	0.000183
Rreb1	-1.551436143	0.009993
Cdo1	-1.54169137	0.000328
Scube2	-1.541043592	0.002242
Strip2	-1.540253285	3.65E-07
Fscn2	-1.540062594	0.000598
Arl5c	-1.534719261	0.01253
Adcy1	-1.531178433	0.02986
B3galt5	-1.530524478	2.98E-07
Fam19a2	-1.528317534	0.01303
Bok	-1.52098212	0.005281
Tiam1	-1.516667042	0.000292
C2cd4a	-1.514610255	0.03914
Pgm5	-1.514534296	0.03146
Arl15	-1.51277943	3.68E-08
Ptpre	-1.50997719	0.000186
Plekhg1	-1.508165109	0.000301
Fam241a	-1.507093187	0.014518
Zfpm2	-1.501431226	0.001037
Kcnab2	-1.49969052	0.012024
RGD1309350	-1.488562592	0.03711
LOC100912481	-1.484027005	0.019747
Ankrd36	-1.48346745	0.003678
Cdh9	-1.478319216	0.001613
Slc38a10	-1.470895823	0.008455
Anln	-1.466465591	0.007964
Lmo2	-1.456586246	0.001487
Aldh1a1	-1.456319389	0.009152
Dagla	-1.447612728	0.005586
Gria1	-1.441107224	2.98E-05
Rprml	-1.440922777	0.000523
Il1rap	-1.434658632	0.007266
Acan	-1.433758122	0.005319
LOC679711	-1.432661013	0.027554
Dph1	-1.422678944	1.37E-12
Tbc1d1	-1.421395797	4.56E-05
Ddo	-1.420982608	5.07E-05
Arhgef26	-1.419219239	1.59E-07
Pdyn	-1.412811459	0.029792
Kcnab1	-1.407427839	3.68E-08
Cnih2	-1.401042257	4.84E-06
Olfml2b	-1.398195014	5.30E-05
Arc	-1.397781408	2.45E-07
RGD1310819	-1.397339133	1.31E-06
Plppr4	-1.390398986	0.004679
Cadm2	-1.382721051	0.005864
Scg2	-1.372191516	0.001354
Dgkh	-1.369091612	0.000929

Ahcyl2	-1.368836194	8.13E-05
Cttnbp2	-1.362915968	4.03E-13
Lgi1	-1.357655318	0.010177
Bcl11b	-1.353659652	0.003551
Grm1	-1.349196928	2.24E-08
Tecta	-1.340058923	0.005463
Mybph	-1.338120582	1.09E-06
Sgpp2	-1.336703322	0.003866
Xkr8	-1.334379111	0.001047
RT1-M1-4	-1.328377279	0.013052
Ptk2b	-1.328363462	0.000322
Frat1	-1.311852482	3.91E-05
Lats2	-1.302601442	0.002643
LOC100362565	-1.299317225	0.044363
Dcc	-1.291772449	4.44E-08
Wasf1	-1.287634641	0.014219
Kcnc3	-1.287625353	0.013052
Nefl	-1.283823635	0.027101
Kctd6	-1.283035906	0.007591
Tbkbp1	-1.282439271	0.002888
Cttn	-1.277893164	2.71E-06
Arhgef25	-1.2759492	0.018552
Exoc3l4	-1.275493581	0.013268
Plxdc1	-1.272324041	5.23E-05
Pmp22	-1.27066855	7.20E-05
Ncdn	-1.269970844	1.48E-07
Nt5dc3	-1.266505753	0.002874
Slit3	-1.265770471	0.001012
Lrp1b	-1.265528672	1.36E-05
Ppil6	-1.262068021	0.006356
Porcn	-1.258441422	0.000293
B3gat2	-1.253931652	8.50E-05
Clptm1l	-1.252856461	0.014948
Creb3l1	-1.24983272	0.000319
Smarcd2	-1.247276484	2.28E-05
Arhgap12	-1.245986586	0.000765
Efna2	-1.243010677	0.020691
Vps29	-1.238105413	0.000678
Fut4	-1.236821905	0.015875
LOC100361087	-1.235236917	0.022265
Ap1s3	-1.232138526	0.04397
LOC103692273	-1.226920295	0.029321
Sumo4	-1.225591954	0.00159
RGD1306556	-1.22521542	0.01662
Arpc2	-1.224572458	0.00012
Camk2b	-1.22221659	7.05E-05
Ptpru	-1.22156395	0.019643
Msx1	-1.220901889	0.000209
Arpc5	-1.219230611	0.002734
Arrdc2	-1.213584592	0.032834
Ehd3	-1.213486391	0.011561
Tagln	-1.211144202	0.000819
Actr3	-1.209707633	2.60E-05
Fhdc1	-1.208697344	0.000177
Lmo1	-1.207295357	0.000253
Faxc	-1.207279565	0.048631

Fam163b	-1.203954538	0.000819
Dll1	-1.194481201	0.000651
F12	-1.192251182	0.012142
Prkcg	-1.189435861	0.040147
Raver2	-1.188235614	0.001282
Cldn11	-1.188081302	0.032557
Ceacam1	-1.187518573	0.001054
Mast3	-1.187016719	5.07E-06
Thbs3	-1.186987932	1.33E-07
Sh3bp5	-1.182339369	0.001667
LOC100910944	-1.181457783	0.042585
Msra	-1.176056327	0.004819
Plxna4	-1.167587324	0.021862
Ugt8	-1.167528459	0.01629
Tesc	-1.164231991	0.000191
Plekhg3	-1.162681932	3.49E-06
Bcl6	-1.161946144	0.016313
Mcm4	-1.158637804	1.87E-06
Sh2d5	-1.157027243	6.83E-06
Slc29a4	-1.156018547	0.000758
Ppfia4	-1.149315687	5.44E-10
Rasgrp1	-1.146475422	0.012788
Ryr3	-1.141036354	2.91E-05
Sox15	-1.140325996	0.006882
Ppp3ca	-1.13679985	0.001037
Tmem38a	-1.133401833	0.000111
Ampd2	-1.13120998	2.80E-08
Adam19	-1.130583882	0.048155
RGD1563354	-1.12469059	2.70E-08
Slc1a1	-1.120371716	0.011268
Hsd17b11	-1.118406763	1.17E-06
Zswim5	-1.113791954	2.24E-08
Kcnq3	-1.113672923	0.02307
Asphd2	-1.111197865	0.033999
Maml2	-1.110132634	0.003337
Abca8a	-1.109874282	0.014372
Lmo3	-1.109768452	4.65E-06
Dock11	-1.109411791	0.001756
Acta2	-1.108570126	0.024399
Agt	-1.106266558	0.000429
Kbtbd11	-1.102897505	0.000139
Plppr2	-1.099762491	0.014338
Gabrb3	-1.097692253	0.045184
Pip5k1b	-1.090660465	0.044366
Rnf128	-1.089497105	0.022265
Slitrk2	-1.089058482	0.047401
Reps1	-1.084831244	0.002174
Agfg2	-1.083456823	2.23E-06
Dcakd	-1.082697828	0.001878
Adamts10	-1.07988799	0.016823
Ehd1	-1.079688214	7.97E-11
Fra10ac1	-1.079440394	5.80E-08
Fgfr1	-1.075689339	0.000133
Eml5	-1.075497897	6.66E-05
Pcdh19	-1.07473215	0.037465
Napa	-1.067469896	0.008105

Inhbb	-1.06640991	0.02017
LRRTM1	-1.065816244	1.51E-06
Lrrn2	-1.062393173	0.001132
Gpr68	-1.061644785	0.016227
Coro7	-1.054926232	3.66E-11
Tmem88b	-1.053888963	0.005958
Cyth3	-1.053585077	0.019434
Efnb3	-1.047765266	0.026461
Ubash3b	-1.047475951	0.000389
Prpf4	-1.044867634	0.022256
Nbea	-1.04133671	0.003635
Clip4	-1.041045263	0.002469
LOC100909455	-1.040292478	0.035149
Ppp4r4	-1.040183458	2.38E-06
Tmcc3	-1.038441648	6.43E-05
Adgrb3	-1.029869786	0.015022
Kndc1	-1.028917319	0.019931
Shbg	-1.028850635	3.86E-05
Rab40b	-1.019020222	0.000828
Mycl	-1.017497351	0.009284
Arl6ip5	-1.015861512	0.02743
Kcnd2	-1.008823098	0.000838
Tnfrsf21	-1.008570893	0.038006
Sptb	-1.006012568	0.02117
Mctp1	-1.000597983	0.030723
Slc25a52	1.001292683	0.038128
Nog	1.00270324	0.004111
Klf6	1.002805352	1.79E-10
LOC108349536	1.00454977	0.013641
Otud1	1.007069854	1.51E-06
Tp53i3	1.007385346	0.010674
Stmn1	1.007445316	2.80E-07
Cabyr	1.007571351	0.042585
Sptbn5	1.007925801	0.047014
Ncam2	1.01103128	0.001351
Elavl4	1.012524497	0.012617
LOC102553140	1.013087305	0.034063
Magi3	1.01516972	0.001667
Hsf4	1.016136163	0.001285
Prrg3	1.017849389	0.011217
Wdr6	1.017913811	0.00015
Klf4	1.020070814	3.70E-05
Slc35f1	1.022562007	0.000663
Il3ra	1.023707088	0.000398
Atg16l2	1.024334743	0.00281
Gstt2	1.025751144	0.023172
LOC680077	1.027532705	0.005123
Hsbp1l1	1.030299604	0.000247
Cntd1	1.031938559	0.001768
Ltc4s	1.034106201	0.001045
Sash3	1.036149318	0.045666
Tmc5	1.037144041	0.001435
Cntnap4	1.039626805	0.000319
Rgs17	1.040583523	1.47E-09
Pou2f2	1.043234908	0.001384
Slc41a2	1.043797511	0.025189

LOC103690234	1.044534627	0.01886
Mrnip	1.045904033	0.01715
Slc8a3	1.049649168	0.005463
Naip5	1.051576874	0.009771
Nlrp3	1.053081859	0.003814
LOC108348250	1.053149773	5.65E-05
MGC114483	1.055050771	0.000969
Crip2	1.055622823	0.030173
RT1-S2	1.057412044	0.049187
Cracr2b	1.059775506	0.009152
Slc6a13	1.059916586	0.009559
Antxr1	1.060508838	0.003649
Trpc3	1.06372519	0.000708
Farp1	1.063852879	9.60E-08
Nipal2	1.065697741	0.000722
Ankrd34b	1.065842603	0.014908
Insyn2b	1.066803755	6.30E-08
Tmem220	1.067718843	0.038902
Lcat	1.069877623	0.000698
Stard10	1.071231759	0.042016
RGD1308544	1.073355951	6.44E-07
Uvssa	1.073431833	0.003649
Arhgap15	1.07493576	0.005306
Kcnh2	1.075627521	0.000544
Chrna5	1.076230745	0.04619
Mip	1.077755839	0.000161
Fam241b	1.080889596	4.64E-06
Pde9a	1.083589945	0.003742
Slc23a3	1.083932902	0.032693
Dtx4	1.086077601	0.011884
Cd40	1.08812932	0.006121
Sema4a	1.088179861	0.000995
Inpp5d	1.094543597	0.00242
Tmem202	1.095190565	0.00164
Man1c1	1.097440157	0.000699
Fli1	1.104836248	0.018139
Ltbp3	1.104944979	1.52E-05
LOC108352688	1.106935998	0.008666
Socs2	1.107489613	0.006031
C1qc	1.10901969	0.019318
Myom3	1.109220986	0.032561
Nav3	1.109757032	0.001415
Mx2	1.114034687	0.007783
Col6a2	1.116845175	5.68E-05
Ptprm	1.117074318	0.047014
Vgll3	1.12226199	0.038109
Sdk2	1.123148316	5.64E-08
Kcnip1	1.123894986	1.33E-05
Ascl2	1.125386336	0.037479
RGD1566368	1.127902009	0.006593
Zfhx4	1.127911687	0.004277
Psmb11	1.130912427	0.023102
Prr5	1.135371822	0.025702
Tigd3	1.136825002	0.0008
LOC108348192	1.138324919	0.025958
Tmsb15b2	1.140277698	0.011738

Cacna2d2	1.141628656	0.012366
Npas2	1.142584344	0.019782
LOC102548478	1.143597716	0.000168
Acr	1.145932384	0.008878
LOC108353290	1.146153	0.032323
Dleu7	1.147814517	0.001741
Gsdma	1.148027874	0.00054
Cdhr3	1.148553721	0.03096
LOC100364265	1.149905672	0.005697
LOC103689949	1.151239968	0.036151
Cdh24	1.155202663	0.000256
Cybrd1	1.157381188	5.61E-05
Arpp19	1.158114772	0.029792
LOC500354	1.159122414	0.014472
Pafah1b3	1.159485078	0.021942
RGD1565117	1.159940344	0.042933
Timp2	1.162233386	0.012142
Ptprf	1.163014589	0.000285
Frmpd3	1.164352531	0.00284
Cxxc4	1.16441715	3.30E-12
LOC691414	1.164527251	0.019561
Arnt2	1.164683997	0.000862
Rtp4	1.165400665	0.023647
Ptpn14	1.165526122	0.000459
Ddx58	1.169608421	0.045034
Dach2	1.170425027	0.000985
Cacna1g	1.171291424	0.011148
Pcdh10	1.178752382	0.00089
Akr1d1	1.183052982	1.35E-09
Cd83	1.184204918	0.00034
Cdc14b	1.189378833	2.21E-06
Nrg1	1.196990722	6.80E-07
LOC103692792	1.197996708	0.000118
Pip5kl1	1.198286782	0.000261
St3gal1	1.200967119	0.034114
H2afy2	1.201536624	2.08E-05
Rtbdn	1.203580472	0.00346
Map3k9	1.209980841	0.045523
N5	1.214090133	0.000882
Adgra1	1.215975797	8.63E-05
Ubxn7	1.216927376	0.03391
Nyap2	1.218529509	5.45E-08
LOC102555814	1.221100317	0.01977
Piwil1	1.221258685	0.000152
Ptafr	1.22811854	0.036187
Fam69c	1.228821152	0.04442
Fbxw10	1.232919371	0.02391
RGD1307461	1.23544859	0.045488
Irf7	1.235742512	0.038785
Ifi44	1.236189248	0.016732
Tox3	1.23674701	0.005054
Sh3pxd2b	1.237174657	9.83E-06
Gpr82	1.239588568	0.030345
Tmem255a	1.240358502	7.62E-06
Ppef2	1.241759442	0.036679
Slc7a3	1.241966237	0.002241

Tle2	1.24322402	3.99E-09
Thrb	1.243257245	9.30E-11
Cfap61	1.247182412	0.049859
Fam92b	1.247963854	0.000389
RGD1560556	1.249065542	6.58E-05
Sec1	1.24986728	0.022839
Chek2	1.256632795	0.009707
Mpeg1	1.258983173	0.027101
LOC501396	1.261876442	0.007193
Mxra7	1.263550913	7.42E-05
Liph	1.26443732	0.048692
Pdzrn4	1.2644793	0.032877
Nrsn1	1.266938994	0.000713
Frmd3	1.267879858	0.00536
LOC103690779	1.271424292	0.006444
Rbms1	1.272904466	0.019665
Elmo1	1.27413964	5.93E-06
Sh3rf3	1.278051503	6.77E-07
Pcdhga2	1.280518889	0.011561
Trpv2	1.282448324	1.26E-09
Arhgap25	1.283392637	0.015643
Gcat	1.286264658	0.001362
Dock2	1.288698814	0.004171
Aadat	1.290854723	0.030428
Clcnkb	1.292021412	0.014001
Myof	1.293307707	0.00046
Oard1	1.298600885	0.027173
Tmem145	1.299832503	0.000429
Pwp2	1.300901256	0.000388
Cdh18	1.306542012	0.031598
Arl4a	1.310369653	2.87E-06
LOC108348167	1.310891986	0.000214
Rwdd2a	1.311747366	0.008146
Trim66	1.315226184	0.00242
LOC108350921	1.320434722	0.000508
RGD1561662	1.322572552	0.047014
Rassf7	1.323208835	0.032992
Dnase1	1.326202253	0.04142
Cables1	1.327399248	0.000878
Vash2	1.327440544	0.006394
Cass4	1.327811974	0.049859
Lrmp	1.332600324	0.0004
LOC684998	1.335116864	0.034017
Emid1	1.338208262	0.021942
Tctex1d1	1.340260861	0.015883
Prph	1.344711435	0.014372
Sh3bp2	1.344954627	0.011561
Tmem119	1.346005333	0.018776
LOC108352191	1.346026559	0.010576
LOC100911699	1.346204065	0.000157
Sema4g	1.347839145	7.28E-08
Tubd1	1.348752451	0.023283
Pdzrn3	1.351754807	3.77E-06
Sox5	1.352122991	4.36E-13
Sync	1.352223536	5.26E-05
Cntn6	1.352934655	0.002367

Etl4	1.35749125	3.37E-05
LOC100912904	1.35808775	0.000715
Spag4	1.362466393	0.013052
Frmd6	1.363158708	0.004252
LOC108353239	1.364596162	3.28E-09
Samd9	1.365032444	2.80E-10
Dbndd1	1.365889854	0.000204
Oasl2	1.36909997	0.032715
Mapk13	1.371125435	0.009561
Usp35	1.374818199	1.04E-17
Tpd52l3	1.37499198	0.004947
Pcdh7	1.376738519	0.012552
Adamts13	1.380442911	0.019568
Lrrtm3	1.380526617	3.25E-06
Shroom3	1.380960081	0.010026
Pnpla3	1.382631138	0.000615
Hsd17b1	1.385846186	0.048495
Adap2	1.389854654	0.004612
Naaladl2	1.39121316	0.00837
Vwa7	1.392222496	0.000503
Impa2	1.392605281	0.03706
Dlx4	1.394707867	0.047014
Smim24	1.395693752	0.017852
Vat1	1.399535433	0.003301
Ch25h	1.400744003	0.017617
Sst	1.401944722	8.60E-21
Mpl	1.402063498	0.0011
Nckap1l	1.40231496	0.019351
Birc3	1.40268126	0.038502
Kcnk13	1.405699827	0.044366
Kcnh6	1.40900201	0.000667
LOC103690508	1.413334772	0.019013
Gipc2	1.417120086	0.032557
Gmfg	1.420451653	0.004357
Art3	1.423602347	0.046253
Sorcs1	1.429042038	0.001757
Slamf8	1.429877632	0.044366
Adamtsl1	1.434941259	0.045488
C2cd4c	1.441535817	2.62E-05
Grip2	1.441776027	9.17E-15
LOC108351576	1.442103569	0.00444
LOC103693202	1.44577323	0.00105
Cdh6	1.445823256	0.044366
LOC100910885	1.446506605	0.003238
Npffr1	1.446935923	0.032857
Cndp1	1.446950025	0.024321
Arhgap45	1.453271755	0.033555
Gpsm1	1.454005974	0.004612
Alb	1.455442181	0.007734
Ramp3	1.45966527	0.036023
Ankrd63	1.463962385	0.038367
Cck	1.468019038	0.016918
Tlr7	1.468054157	0.009773
Fes	1.474561141	0.045184
Grm4	1.481595845	0.009342
Siglec1	1.485218748	0.004136

Gap43	1.490493367	6.02E-10
Ccl24	1.498367787	0.002507
Rab3b	1.499598852	3.45E-12
Plppr3	1.503819462	0.007247
Xaf1	1.506617869	0.008377
Galns	1.507574103	0.000162
Vat1l	1.50886784	0.022783
P2ry2	1.510284291	0.02137
Enkur	1.510563314	0.003695
Herc6	1.512970837	2.23E-06
Cd37	1.515773769	0.043847
Grem2	1.516523426	1.57E-08
Prkg2	1.522952358	0.013681
LOC100361655	1.525554305	0.000256
Cpne5	1.538180254	0.033244
Rnd1	1.540161197	0.027481
Tmprss2	1.549799798	0.016927
Lsmem2	1.5505368	0.043547
Ldb2	1.551607075	1.88E-07
C2	1.551960073	0.014963
Nr6a1	1.552368582	0.03914
Itih3	1.553492015	0.000437
Tent5a	1.554840909	0.010941
LOC681177	1.555887894	0.020922
Igf1	1.55732147	0.00314
Smoc1	1.559666259	0.00123
Nlrc4	1.564342914	0.0437
Trem2	1.567795018	0.032824
Nr1h4	1.569403223	0.023147
LOC108349482	1.570943501	0.002071
Sdk1	1.575860038	0.00536
Il7r	1.577628411	0.003458
Coro6	1.583524183	0.000149
Rsph1	1.584121853	0.043933
Fam131c	1.587068206	1.02E-05
Abcc3	1.597373322	0.012626
Chtf18	1.601765705	0.009097
Brip1	1.602213773	0.025716
Adamtsl2	1.603682412	1.67E-12
Dact2	1.604111695	0.004947
Fbxo17	1.606870463	0.030164
Dusp1	1.61113535	0.003169
Rassf5	1.611375602	0.000242
Rai14	1.614502165	0.011708
RGD1566251	1.614822546	0.000215
Ass1	1.615402013	7.36E-06
Adgrd1	1.617492249	0.008438
Klra1	1.619102687	9.82E-06
Ephx4	1.623373234	0.000713
Limd1	1.623847233	0.021793
Zfhx3	1.625944629	0.019752
Clnk	1.626157702	0.014372
Gpc6	1.634290618	0.008868
Bhmt	1.63527547	4.90E-05
Cdk6	1.636367131	0.01272
Prokr2	1.638685203	0.026546

Mreg	1.639803194	0.004742
Gng13	1.640206755	0.000317
Kazald1	1.642028807	0.031077
Btbd11	1.646385033	2.14E-05
Sntb1	1.648561704	0.000529
Dok5	1.65105338	4.36E-13
Nhs	1.65767168	0.002685
Gabra3	1.65896117	1.06E-05
Coro2a	1.675087325	3.56E-08
Col2a1	1.680097952	0.006194
Zic3	1.683740496	0.016758
Lrmda	1.68686901	0.045355
Sh2b2	1.687594248	0.000665
Cdh4	1.690898378	0.011738
Kcnk3	1.693730662	0.001415
LOC685680	1.704056218	0.005281
Th	1.714495575	0.000769
Hpcal1	1.72117665	2.29E-26
Runx1	1.722657925	0.000374
Ftcd	1.726218032	0.002905
Igfbp6	1.734226408	0.002955
Lgals7	1.742506752	0.038104
LOC103690039	1.742529533	0.000304
Cit	1.743134703	0.000278
Mns1	1.746051533	0.020302
Sstr1	1.754082759	0.016921
LOC103689995	1.756413455	0.00128
Sla	1.764087885	0.007043
Tnfaip8l2	1.765116169	0.00128
Tmem270	1.766203737	1.10E-05
RGD1563056	1.768143963	0.00142
Camk2n1	1.768529671	0.002017
Steap3	1.768765163	0.006241
Muc6	1.768990227	0.033507
Fxyd6	1.771065801	5.05E-06
LOC100911109	1.775419224	4.57E-05
Prkcd	1.775906279	0.000362
Bean1	1.776485536	0.00128
LOC108348074	1.777714084	5.04E-08
Adssl1	1.778505868	0.007303
Maats1	1.780941087	0.008987
Hcn3	1.781361338	6.29E-06
Nle1	1.785278865	0.037592
Ifit3	1.786018347	1.01E-05
Slfn13	1.788796163	0.034876
LOC502684	1.788841257	0.031289
Pmfbp1	1.805686806	0.028022
Olfm2	1.82069061	0.000242
Mroh7	1.833976063	0.025635
Lzts1	1.840202763	2.64E-07
Zic1	1.842965546	0.034027
Zan	1.848858225	0.034227
LOC102551559	1.850136286	0.005725
Opn3	1.85377164	0.025404
Igsf21	1.866602065	3.45E-15
Lilra5	1.868915498	0.041334

Cdkn3	1.870230055	0.047134
Treml1	1.874640325	0.002131
Ireb2	1.875700103	0.013241
Ccl9	1.87763248	0.015292
LOC103693257	1.877701762	0.038367
Ttc22	1.878128155	3.00E-06
Gsg1l	1.879780193	0.018876
Gli1	1.881365425	0.008271
Stard8	1.883294082	0.000309
Gpx3	1.883916485	8.95E-08
Fnbp1l	1.884535686	0.010719
Cd6	1.895596023	0.002677
Gng4	1.903175033	4.24E-08
Tfap2b	1.906193738	0.028286
Pou3f2	1.918343602	0.000158
Stpg1	1.919950247	0.043076
Wnt3	1.923749357	0.027599
Cacng5	1.924124317	0.019693
LOC100912658	1.930090488	0.014134
Hmcn2	1.930720718	0.00035
Muc2	1.931067838	0.009683
Fyb1	1.932404791	0.016066
Serpine1	1.932712914	0.026364
Mef2c	1.936702362	7.55E-07
Stx1a	1.938021973	0.000301
Tspan1	1.947316606	0.000127
Garnl3	1.954366415	4.05E-08
Batf3	1.958725706	0.020571
RGD1565071	1.96053567	0.000429
Hsh2d	1.965121984	0.011571
LOC100910088	1.968506643	0.008362
Blk	1.968551385	0.006559
Nat8f3	1.976148155	0.000906
LOC108348429	1.976369633	5.14E-05
Tec	1.983223594	0.002177
Grid2ip	1.98783201	0.003254
Gpat2	1.9878434	0.00243
Fuom	1.988357252	0.001415
Il36rn	1.993620887	0.016011
Rasgef1b	2.000440172	0.000878
Mctp2	2.002139346	0.023448
Frem1	2.00395756	0.019899
Postn	2.006261979	0.000389
Tshz3	2.011702764	0.000363
Efcab6	2.020838612	5.86E-06
Sall1	2.020882734	0.000246
Col13a1	2.024170208	0.036215
Hrasls	2.029052315	0.003453
Iqca1	2.034060866	0.015557
Sfrp4	2.036862916	0.003315
Als2cr12	2.037851525	0.021518
Magel2	2.038625307	0.047014
Zfp804b	2.048907176	0.006503
Was	2.051553556	0.023419
Ptprt	2.056862215	6.70E-11
Stac2	2.05862886	7.55E-05

Scube1	2.066479434	0.000533
LOC103692936	2.069989267	0.023843
Mas1l	2.071332347	0.005848
LOC684871	2.083692968	0.003514
Rgs1	2.085472699	0.010121
Catsperz	2.092328803	0.00091
Cmtm4	2.101668733	0.019517
Nppa	2.108676743	0.000323
Gckr	2.109093104	0.026863
Dlx1	2.109405161	0.045488
Spint3	2.110646635	0.03721
Myh1	2.112155184	0.00161
Trdn	2.112612719	0.000137
Ifi27l2b	2.114956472	0.000912
Plcb4	2.117556035	8.25E-07
Cplx3	2.125350947	6.75E-08
Klf5	2.126380686	0.000141
Ipcef1	2.131206785	8.90E-05
Sh2d7	2.133200797	0.025267
Doc2a	2.134252754	1.57E-05
Cckbr	2.140397119	0.000191
Ramp1	2.141577974	0.015875
Zik1	2.154888764	0.017068
Meis1	2.157964508	0.004998
Glt8d2	2.158686328	0.033624
Slfn4	2.161148214	0.014438
Bmp2	2.161999046	0.000929
Dusp26	2.163149026	1.81E-11
Tgif1	2.167859996	0.00159
Lilrb3	2.168949092	0.009342
Satb2	2.17648766	0.010455
Pla2g4e	2.179094391	0.000527
LOC108348336	2.180469149	0.032076
Plekhm3	2.188363131	0.012055
Gipr	2.199084619	1.17E-07
Lamp5	2.200999156	0.01715
LOC108348181	2.208761815	0.026222
Cd247	2.209797636	0.007923
Tox	2.21667289	2.73E-09
Cdh7	2.217601753	1.79E-06
Plaur	2.228903883	0.027918
Wdfy4	2.241613231	0.007923
Catsperg	2.244523971	9.01E-08
Tnnc2	2.253213159	4.92E-06
Zic4	2.253933064	0.035626
Cd226	2.269195448	0.038393
Zfp36l3	2.2841843	0.041663
Olfm4	2.296416853	0.003635
Taf11	2.297212059	0.008912
Epop	2.299177471	0.000317
Arhgap6	2.303587242	0.021686
Col9a1	2.309225859	1.66E-07
LOC300308	2.309318076	2.90E-08
Tnc	2.311655668	0.023516
Plekhg6	2.316172377	3.06E-05
Ret	2.316880749	0.018552

Vstm5	2.320090975	0.031686
Lrrc17	2.322744149	0.033437
Lin28b	2.323940112	0.000489
Ptgfrn	2.327697215	0.00019
Gcdh	2.335005313	0.003755
Kctd8	2.33946463	0.011417
Apold1	2.348975263	0.00331
Gkn2	2.361280197	0.002531
Grp	2.365398972	0.000949
Cep295nl	2.380135786	6.85E-09
Cd4	2.386837194	0.025606
Myh3	2.390050664	0.00646
Tdrp	2.392636407	0.010607
Krt1	2.393585473	0.037465
Kif23	2.396166946	0.022783
Srpk3	2.422521464	2.87E-06
Vwc2l	2.425676611	1.34E-05
Nxph3	2.4350453	0.000166
Smim35	2.437072308	0.021425
Itgb7	2.438606563	0.001814
Tmem91	2.441938818	0.001667
Gpr153	2.487240672	6.29E-06
Gpr15	2.494115394	2.76E-07
Pitpnm3	2.496161529	1.31E-05
Barx2	2.503196917	0.017321
LOC102546864	2.519005798	0.02493
Pstpip1	2.519510397	0.0002
LOC102556277	2.521274161	0.022857
Aunip	2.523184967	0.028428
Dpp10	2.526165852	2.08E-21
Klhl1	2.531721062	8.66E-05
Ano2	2.534872459	0.047473
Cxcl17	2.541769048	0.018947
Arhgap18	2.556642454	0.001286
RGD1561149	2.558087744	7.83E-06
RGD1563400	2.576474799	0.003481
Tpx2	2.577648107	0.001746
LOC306079	2.581144746	0.035568
Rsl1	2.583391374	0.012099
P4ha3	2.584746732	3.30E-12
Sulf1	2.614741674	0.039696
LOC100366216	2.622514296	0.032824
Zbp1	2.635172287	0.046435
Baiap3	2.638129984	0.001762
Cdpf1	2.642683027	0.007275
Yju2	2.655635749	0.004291
Mael	2.658028354	0.023076
Hs3st2	2.674564996	8.55E-11
Adprhl1	2.687225074	0.007492
Col6a3	2.69115503	9.40E-07
Neu2	2.695895857	0.047693
Rexo5	2.700996982	0.003796
Wfdc6a	2.702235872	0.028428
Tmem196	2.712887205	1.09E-06
LOC102555341	2.714966336	1.64E-05
Tpbg	2.716375538	3.30E-12

Chrna4	2.718370747	4.84E-06
Gucy1a1	2.741847649	0.0011
Gfra2	2.750938937	7.54E-17
Rgs4	2.751749702	2.28E-05
Crhr1	2.760977132	1.26E-10
Kcnt1	2.766050759	1.53E-05
Otof	2.768973223	1.01E-05
Il1rapl2	2.774782845	0.006047
Meis2	2.776596449	0.000851
Cbln2	2.777114762	0.000585
Cdhr1	2.793788603	0.001552
LOC100911326	2.823823651	0.021425
Lamc2	2.837287995	1.33E-05
Slc16a12	2.845701539	0.000819
Klhdc8a	2.852807635	0.00019
Calca	2.855882377	0.001671
LOC102546678	2.864107895	1.29E-08
Rprm	2.866384758	5.76E-08
Gtse1	2.866804006	0.003783
LOC103692555	2.875788125	0.0001
Cobl	2.884669036	0.000107
Dtl	2.886719819	0.006293
C3	2.906324412	0.004458
LOC103690131	2.915016558	0.02391
Togaram2	2.918982054	0.006872
Ntsr1	2.919409681	0.016682
Plg	2.926119024	0.012539
Ccl4	2.947974095	0.042452
Gimap9	2.952959864	0.000278
Cux2	2.978055792	4.55E-09
LOC102555624	2.982107106	0.044366
Plcxd3	2.982306645	0.00837
Pcdh11x	2.999430177	3.91E-05
Steap4	3.003685404	0.049814
Slc39a6	3.018782087	0.018126
Slc1a4	3.020881007	0.013106
Sp8	3.021500426	0.018253
Tekt4	3.024717563	0.002294
Mx1	3.053795105	0.004683
LOC680910	3.06614801	0.000991
Camk2d	3.073972272	9.99E-58
Car10	3.076387357	0.000185
Smco4	3.110018878	0.036767
Bmper	3.114003445	6.73E-10
Exoc1l	3.124017369	0.000915
Cldn23	3.134743664	0.023073
Fhod3	3.145101514	5.42E-15
Sema3a	3.150883819	2.90E-08
Efna5	3.157568932	3.68E-08
Gnat1	3.1740311	0.031838
Fcrl1	3.174211521	0.00837
Ecel1	3.18025312	0.012083
Dlk1	3.265314249	0.022604
Cwh43	3.294049173	0.00202
Fap	3.3113833	0.018876
Fibin	3.331698993	0.001347

Hs3st5	3.340528545	0.000819
Gpr52	3.355691798	0.01444
Fam205a	3.371277532	0.020058
Atp2b4	3.409892134	3.14E-17
Rln1	3.474566393	0.014189
Tcerg1l	3.484121686	1.08E-09
Trim54	3.493386086	0.00108
Twist2	3.518014568	0.009106
Slc6a5	3.535556317	0.038622
Tet1	3.538401418	0.000833
Ltb	3.563630532	0.000451
LOC100912070	3.588979559	0.006121
Gkn3	3.594900831	0.01536
Ripor3	3.59545427	0.000116
Dpp4	3.601672404	0.000285
Cyp11b2	3.612121346	1.43E-05
F10	3.623706609	0.005988
Col24a1	3.638165656	0.000162
Dio3	3.680568145	0.038789
Atp6ap1l	3.708095605	0.004593
Lbhd2	3.714988474	0.011621
Rph3al	3.716581561	0.00592
Prdm12	3.77443637	0.028913
RGD1561231	3.855214638	0.009106
Plcg2	3.877415344	4.74E-05
RGD1561143	3.919409206	0.031289
Cxcl13	3.919837931	0.00536
Socs6	3.920977221	0.001362
Rd3l	3.973175732	0.006121
Adra1b	3.98387743	4.24E-08
Rasgef1c	3.992379042	8.55E-10
Enthd1	4.014104175	0.022265
Mab21l2	4.072394128	0.014088
Ovol2	4.082026939	0.000601
Crhr2	4.116619438	4.01E-07
Gabrq	4.150231285	0.004742
Dmgdh	4.151583946	0.0011
Allc	4.169856355	0.001291
Ush2a	4.248368884	0.005988
Samsn1	4.26722529	0.000188
Adra2a	4.293951499	2.39E-07
Ucma	4.331050694	0.008754
LOC108349608	4.371723027	0.003804
Oas1f	4.395645005	0.034876
Cyp26a1	4.395717173	7.49E-07
Spp1	4.416168772	0.023419
Klhdc4	4.418196217	0.001125
Zfp773-ps1	4.526147291	3.27E-05
LOC108350654	4.574569931	0.002874
Vill	4.578421707	3.84E-05
Sprr1a	4.68105019	0.023758
LOC100910579	4.752619715	0.02667
LOC684773	4.822043234	0.003678
Atp10b	4.901150153	0.000359
Lypd5	4.938844779	4.88E-05
Olr59	4.97597219	2.32E-07

Shisal2b	5.044397735	0.003202
Krt71	5.04624916	0.013887
Unc45b	5.051636581	0.036721
Ptchd4	5.101773989	0.000226
LOC103693051	5.132273198	0.000899
Scn5a	5.187626228	6.02E-05
Adam33	5.194006156	0.002759
Themis2	5.199271574	0.000116
Otoa	5.201418238	0.000518
Tsku	5.208809077	0.000259
LOC102552360	5.223555319	0.000256
Gml	5.264341259	0.002861
Hrh2	5.330480021	0.002723
Lilrb3b	5.361251382	0.043517
Mospd4	5.369273861	0.00225
LOC102555038	5.373330399	0.000223
Sycp2l	5.515800973	0.006121
LOC100910278	5.681703363	0.001612
LOC103692344	5.683655032	0.018759
LOC680316	5.688994336	0.001418
Oas2	5.714544202	1.53E-05
LOC108348048	5.745897664	0.003018
Ocm2	5.755001604	0.000571
Slamf9	5.7827462	0.013876
Krt77	5.918503146	0.001435
Serpinb10	5.935625313	0.003706
Tmem74b	5.960354388	8.66E-05
Scn11a	6.014136246	0.000372
Entpd1	6.131148683	0.048937
LOC100360779	6.239182448	0.001763
Osm	6.240307027	0.000348
LOC102553691	6.240322178	0.008553
Six3	6.26614977	0.001675
LOC100910462	6.35919098	0.022555
Cyp11b3	6.435667666	0.009767
Prdm13	6.48455026	0.004954
Xkr5	6.508207325	0.00019
LOC100910068	6.665213677	2.89E-05
Ly6k	6.665213677	2.89E-05
Ppp1r3e	6.803130723	0.000332
RGD1561730	6.943793603	0.000579
Clec2g	7.114255531	0.000538
Magea8	7.284005838	0.000481
Prss8	7.347900369	3.48E-05
LOC103694046	7.390326525	0.001384
LOC108349944	7.638202826	0.007217
LOC102547033	7.654659444	0.012012
Stat4	7.712809505	1.92E-05
LOC680624	8.10903531	0.01287
LOC100909897	8.493481246	6.77E-05
LOC103693456	8.535610509	0.005628
Tssk1b	8.691345021	0.019587
LOC108348129	8.791104891	0.033646
LOC100909599	9.03936126	0.00225
Anxa13	9.255901411	0.016427
Trim43a	9.477516107	1.31E-06

LOC102549344	9.575605494	0.000713
LOC103690016	9.626820385	1.53E-05
Olr1653	9.721686928	0.00516
Spata32	10.05838443	3.41E-05
Vom2r28	10.68338415	0.013981
Klrk1	11.27883163	8.31E-05
Stpg2	11.37304342	1.14E-05
LOC102550456	23.19593467	1.98E-07
Sfta2	25.14221918	1.30E-08
LOC686143	30.32386543	1.62E-12
