# Supplementary material for: Transcriptomic Profiling of Electroacupuncture Regulating the Molecular Network in Hippocampus of Rats with Cerebral Ischemia-Reperfusion Injury
Source: Evid Based Complement Alternat Med. 2022 Sep 2;2022:6053106. doi: 10.1155/2022/6053106 (PMC9463016; doi:10.1155/2022/6053106)
Supplement: Supplementary Materials — Table S1: differentially expressed genes of Model/Sham group; Table S2: preliminary enrichment results of Model/Sham group; Table S3: differentially expressed genes of EA/Model group; Table S4: upregulated gene analysis; Table S5: downregulated gene analysis; Table S6: all gene analysis. [file 6053106.f1.zip › Table S2 (1).pdf]

Table S2 Preliminary enrichment results of M

**Category**	**Term**
GO Biological Processes	GO:0043269
GO Biological Processes	GO:0000902
GO Biological Processes	GO:0034765
GO Biological Processes	GO:0034762
GO Biological Processes	GO:0034330
GO Biological Processes	GO:0099536
GO Biological Processes	GO:0031175
GO Biological Processes	GO:0032989
GO Biological Processes	GO:0048812
GO Biological Processes	GO:0120039
GO Biological Processes	GO:0099537
GO Biological Processes	GO:0048858
GO Biological Processes	GO:0044057
GO Biological Processes	GO:0000904
GO Biological Processes	GO:0006935
GO Biological Processes	GO:0042330
GO Biological Processes	GO:0032990
GO Biological Processes	GO:0048667
GO Biological Processes	GO:0061564
GO Biological Processes	GO:0007420
GO Biological Processes	GO:0051963
GO Biological Processes	GO:0007423
GO Biological Processes	GO:0042391
GO Biological Processes	GO:0007409
GO Biological Processes	GO:0050808
GO Biological Processes	GO:0007411
GO Biological Processes	GO:0097485
GO Biological Processes	GO:0007268
GO Biological Processes	GO:0098916
GO Biological Processes	GO:0043408
KEGG Pathway	hsa04080
KEGG Pathway	hsa04020
KEGG Pathway	hsa04360
KEGG Pathway	hsa04713
KEGG Pathway	hsa04925
KEGG Pathway	hsa04024
KEGG Pathway	hsa05031
KEGG Pathway	hsa04750
KEGG Pathway	hsa04512
KEGG Pathway	hsa04720
KEGG Pathway	hsa05202
KEGG Pathway	hsa04724
KEGG Pathway	hsa04730
KEGG Pathway	hsa04261
KEGG Pathway	hsa04310
KEGG Pathway	hsa05033
KEGG Pathway	hsa04015
KEGG Pathway	hsa04666
KEGG Pathway	hsa04010
KEGG Pathway	hsa04151
KEGG Pathway	hsa04728
KEGG Pathway	hsa04916
KEGG Pathway	hsa04270
KEGG Pathway	hsa04974

KEGG Pathway	hsa05135
KEGG Pathway	hsa04921
KEGG Pathway	hsa04934
KEGG Pathway	hsa04971
KEGG Pathway	hsa05412
KEGG Pathway	hsa04060
KEGG Pathway	hsa05414
KEGG Pathway	hsa04510
KEGG Pathway	hsa04723

Model/Sham group

Description	Pvalue	Enrichmen
regulation of ion transport	1E-20	3.4
cell morphogenesis	1E-20	3.4
regulation of ion transmembrane transport	1E-17	3.6
regulation of transmembrane transport	1E-16	3.3
cell junction organization	1E-16	3.6
synaptic signaling	1E-15	3.6
neuron projection development	1E-15	3.1
cellular component morphogenesis	1E-15	3.3
neuron projection morphogenesis	1E-15	3.6
plasma membrane bounded cell projection morphogenesis	1E-14	3.5
trans-synaptic signaling	1E-14	3.7
cell projection morphogenesis	1E-14	3.5
regulation of system process	1E-14	3.2
cell morphogenesis involved in differentiation	1E-14	3.3
chemotaxis	1E-14	3.3
taxis	1E-14	3.3
cell part morphogenesis	1E-14	3.4
cell morphogenesis involved in neuron differentiation	1E-13	3.6
axon development	1E-13	3.7
brain development	1E-13	2.8
regulation of synapse assembly	1E-13	7
sensory organ development	1E-13	3.1
regulation of membrane potential	1E-13	3.4
axonogenesis	1E-12	3.6
synapse organization	1E-11	3.9
axon guidance	1E-11	4.3
neuron projection guidance	1E-11	4.2
chemical synaptic transmission	1E-11	3.3
anterograde trans-synaptic signaling	1E-11	3.3
regulation of MAPK cascade	1.26E-08	2.4
Neuroactive ligand-receptor interaction	1E-10	3.4
Calcium signaling pathway	1E-09	3.8
Axon guidance	5.01E-09	4.1
Circadian entrainment	7.94E-08	5.1
Aldosterone synthesis and secretion	1E-07	5.1
cAMP signaling pathway	2E-07	3.4
Amphetamine addiction	2.51E-07	5.9
Inflammatory mediator regulation of TRP channels	5.01E-07	4.8
ECM-receptor interaction	5.01E-06	4.6
Long-term potentiation	7.94E-06	5.1
Transcriptional misregulation in cancer	1.58E-05	3.1
Glutamatergic synapse	2E-05	3.8
Long-term depression	2E-05	5.2
Adrenergic signaling in cardiomyocytes	2.51E-05	3.3
Wnt signaling pathway	3.16E-05	3.2
Nicotine addiction	3.16E-05	6.2
Rap1 signaling pathway	5.01E-05	2.8
Fc gamma R-mediated phagocytosis	6.31E-05	3.9
MAPK signaling pathway	7.94E-05	2.4
PI3K-Akt signaling pathway	7.94E-05	2.3
Dopaminergic synapse	0.0001	3.3
Melanogenesis	0.0001	3.7
Vascular smooth muscle contraction	0.0001	3.3
Protein digestion and absorption	0.000126	3.6

Yersinia infection	0.000126	3.2
Oxytocin signaling pathway	0.000126	3
Cushing syndrome	0.000158	3
Gastric acid secretion	0.000158	4.1
Arrhythmogenic right ventricular cardiomyopathy	0.000158	4
Cytokine-cytokine receptor interaction	0.000251	2.3
Dilated cardiomyopathy	0.000251	3.6
Focal adhesion	0.000316	2.6
Retrograde endocannabinoid signaling	0.000316	2.9

Counts	Genes
76	ADRA2A|AGT|ARG1|ATP2A1|ATP2B4|CAMK2B|CAMK2D|CD4|CEBPB|CHRNA4|CLC
74	ADCY1|BCL6|CCK|CDH4|CDH6|CDH7|CDH9|CDH18|DCC|EFNA2|EFNA5|EFNB3|ELAV
59	ADRA2A|AGT|ARG1|ATP2A1|ATP2B4|CAMK2D|CLCNKB|CRHR1|EPHB2|PTK2B|GRI
64	ADRA2A|AGT|ARG1|ATP2A1|ATP2B4|C3|CAMK2D|CLCNKB|CRHR1|EPHB2|PTK2B|G
56	ARHGAP6|ADGRB3|C1QC|C3|CDH6|CDH7|CDH9|CDH18|DSP|EFNA5|CTTN|EPHB2|PT
52	DAGLA|CHRNA2|CHRNA4|CHRNA5|EFNB3|EPHB2|GABRA3|GABRA5|GABRB3|GRIA
64	ADCY1|BLK|CCK|CD3E|CDH4|DCC|EFNA2|EFNA5|EFNB3|ELAVL4|CTTN|EPHA7|EPH
60	ADCY1|CCK|CDH4|DCC|EFNA2|EFNA5|EFNB3|ELAVL4|CTTN|EPHA7|EPHA8|EPHB2|
52	ADCY1|CCK|CDH4|DCC|EFNA2|EFNA5|EFNB3|ELAVL4|CTTN|EPHA7|EPHA8|EPHB2|
52	ADCY1|CCK|CDH4|DCC|EFNA2|EFNA5|EFNB3|ELAVL4|CTTN|EPHA7|EPHA8|EPHB2|
49	DAGLA|CHRNA2|CHRNA4|CHRNA5|EFNB3|EPHB2|GABRA3|GABRA5|GABRB3|GRIA
52	ADCY1|CCK|CDH4|DCC|EFNA2|EFNA5|EFNB3|ELAVL4|CTTN|EPHA7|EPHA8|EPHB2|
59	ADRA1B|ADRA2A|AGT|ATP2A1|ATP2B4|CALCA|CAMK2B|CAMK2D|CRHR1|DSP|CT
55	ADCY1|CCK|CDH4|DCC|EFNA2|EFNA5|EFNB3|ELAVL4|EPHA7|EPHA8|EPHB2|GAP43|
54	CALCA|CDH4|LYST|DCC|DOCK2|DPP4|EFNA2|EFNA5|EFNB3|EPHA7|EPHA8|EPHB2|F
54	CALCA|CDH4|LYST|DCC|DOCK2|DPP4|EFNA2|EFNA5|EFNB3|EPHA7|EPHA8|EPHB2|F
52	ADCY1|CCK|CDH4|DCC|EFNA2|EFNA5|EFNB3|ELAVL4|CTTN|EPHA7|EPHA8|EPHB2|
47	ADCY1|CCK|CDH4|DCC|EFNA2|EFNA5|EFNB3|ELAVL4|EPHA7|EPHA8|EPHB2|GAP43|
45	ADCY1|CCK|CDH4|DCC|EFNA2|EFNA5|EFNB3|EPHA7|EPHA8|EPHB2|GAP43|TNC|LA
66	ADCY1|ZFHX3|ATP2B4|BMP2|BOK|CD3E|CDK6|DLX1|EFNA2|ELAVL4|EPHA7|EPHB2
23	ADGRB3|EFNA5|EPHA7|EPHB2|GPC4|GHSR|IL1RAP|MEF2C|NTRK1|TPBG|GPC6|FAR
55	ASCL2|ATP2B4|BMP2|CEBPD|COL2A1|CRYGD|CYP1B1|DIO3|EPHB2|FBN1|GABRA5|
47	BOK|CAMK2D|CHRNA2|CHRNA4|CHRNA5|DSP|PTK2B|GABRA3|GABRA5|GABRB3|G
40	ADCY1|CCK|CDH4|DCC|EFNA2|EFNA5|EFNB3|EPHA7|EPHA8|EPHB2|GAP43|LAMC2|
36	ADGRB3|C1QC|C3|EFNA5|CTTN|EPHB2|GPC4|GABRB3|NRG1|TNC|IL1RAP|NEFL|PTP
31	CDH4|DCC|EFNA2|EFNA5|EFNB3|EPHA7|EPHA8|EPHB2|GAP43|LAMC2|NTRK1|PTPR
31	CDH4|DCC|EFNA2|EFNA5|EFNB3|EPHA7|EPHA8|EPHB2|GAP43|LAMC2|NTRK1|PTPR
42	CHRNA2|CHRNA4|CHRNA5|GABRA3|GABRA5|GABRB3|GRIA1|GRIN2A|GRM1|GRM4
42	CHRNA2|CHRNA4|CHRNA5|GABRA3|GABRA5|GABRB3|GRIA1|GRIN2A|GRM1|GRM4
52	ACTA2|ADRA1B|ADRA2A|AGT|CEACAM1|BMP2|CD4|CD40|DUSP1|DUSP5|DUSP6|EP
39	ADRA1B|ADRA2A|AGT|C3|CALCA|CCK|CCKBR|CHRNA2|CHRNA4|CHRNA5|CRHR1|
29	ADCY1|ADRA1B|ATP2A1|ATP2B4|CAMK2B|CAMK2D|CCKBR|PTK2B|FGFR1|FGFR4|
24	CAMK2B|CAMK2D|DCC|EFNA2|EFNA5|EFNB3|EPHA7|EPHA8|EPHB2|FES|PLCG2|PPP
16	ADCY1|CAMK2B|CAMK2D|GNG4|GRIA1|GRIA3|GRIN2A|GUCY1A1|PLCB4|PRKCG|P
16	ADCY1|AGT|ATP2B4|DAGLA|CAMK2B|CAMK2D|CYP11B2|KCNK3|NPPA|NR4A2|PLC
24	ADCY1|ATP2A1|ATP2B4|CAMK2B|CAMK2D|CREBBP|CRHR1|CRHR2|GHSR|GIPR|GL
13	CAMK2B|CAMK2D|GRIA1|GRIA3|GRIN2A|PDYN|PPP3CA|PRKCG|STX1A|TH|ARC|CR
15	ADCY1|CAMK2B|CAMK2D|IGF1|IL1RAP|NTRK1|P2RY2|PLCB4|PLCG2|PRKCD|PRKC
13	COL2A1|COL6A2|COL6A3|COL9A1|TNC|ITGB4|ITGB7|LAMC2|SPP1|THBS3|ITGA11|F
11	ADCY1|CAMK2B|CAMK2D|CREBBP|GRIA1|GRIN2A|GRM1|PLCB4|PPP3CA|PRKCG|C
19	BIRC3|BCL6|RUNX1|CD40|CEBPB|DUSP6|FLI1|GRIA3|IGF1|ITGB7|LMO2|MEF2C|MEIS
14	ADCY1|GNG4|GRIA1|GRIA3|GRIN2A|GRM1|GRM4|PLCB4|PPP3CA|PRKCG|SLC1A1|H
10	CRHR1|GRIA1|GRIA3|GRM1|GUCY1A1|IGF1|PLCB4|PRKCG|PRKG2|PLA2G4E
16	ADCY1|ADRA1B|AGT|ATP2A1|ATP2B4|CAMK2B|CAMK2D|PLCB4|MAPK13|SCN5A|S
17	CAMK2B|CAMK2D|CREBBP|GPC4|ROR2|PLCB4|PPP3CA|PRKCG|SFRP4|TLE1|TLE2|W
8	CHRNA4|GABRA3|GABRA5|GABRB3|GRIA1|GRIA3|GRIN2A|GABRQ
19	ADCY1|EFNA2|EFNA5|FGFR1|FGFR4|FYB1|GRIN2A|IGF1|KIT|PLCB4|PRKCG|MAPK13
12	INPP5D|PLCG2|PRKCD|PRKCG|WAS|PIP5K1B|WASF1|ARPC5|ACTR3|ARPC2|VAV3|PL
23	DUSP1|DUSP5|DUSP6|EFNA2|EFNA5|FGFR1|FGFR4|IGF1|IL1RAP|KIT|STMN1|MEF2C|
26	CDK6|COL2A1|COL6A2|COL6A3|COL9A1|EFNA2|EFNA5|FGFR1|FGFR4|GNG4|TNC|IG
14	CAMK2B|CAMK2D|GNG4|GRIA1|GRIA3|GRIN2A|PLCB4|PPP3CA|PRKCG|MAPK13|TH
12	ADCY1|CAMK2B|CAMK2D|CREBBP|KIT|PLCB4|PRKCG|WNT2|WNT3|WNT3A|CREB3
14	ACTA2|ADCY1|ADRA1B|AGT|CALCA|GUCY1A1|NPPA|PLCB4|PRKCD|PRKCG|RAMP
12	COL2A1|COL6A2|COL6A3|COL9A1|COL13A1|DPP4|KCNJ13|PGA5|SLC1A1|SLC8A3|CC

14	CD4|PTK2B|FYB1|MAPK13|WAS|PIP5K1B|ELMO1|ARPC5|ACTR3|ARPC2|VAV3|NLRC[illegible]
15	ADCY1|CAMK2B|CAMK2D|GUCY1A1|MEF2C|NPPA|PLCB4|PPP3CA|PRKCG|RYR3|CA[illegible]
15	ADCY1|AGT|CAMK2B|CAMK2D|CDK6|CRHR1|CRHR2|KCNK3|PLCB4|WNT2|WNT3|C[illegible]
10	ADCY1|CAMK2B|CAMK2D|CCKBR|HRH2|PLCB4|PRKCG|SST|CALML4|SLC9A4
10	ATP2A1|DSP|ITGB4|ITGB7|PKP2|SLC8A3|CACNA2D2|ITGA11|CACNG5|CACNG8
22	BMP2|CD4|CD40|GDF10|IL1RAP|IL3RA|IL7R|CXCR1|IL16|INHBB|LTB|MPL|OSM|CCL4|[illegible]
11	ADCY1|AGT|ATP2A1|IGF1|ITGB4|ITGB7|SLC8A3|CACNA2D2|ITGA11|CACNG5|CACN[illegible]
17	BIRC3|COL2A1|COL6A2|COL6A3|COL9A1|TNC|IGF1|ITGB4|ITGB7|LAMC2|PRKCG|SP[illegible]
14	ADCY1|DAGLA|GABRA3|GABRA5|GABRB3|GNG4|GRIA1|GRIA3|GRM1|PLCB4|PRKC[illegible]

NKB|CRHR1|EPHB2|PTK2B|GRIN2A|GRP|HPCA|HTR1A|IL16|KCNC3|KCND2|KCNH2|KCNJ13|KCN
L4|CTTN|EPHA7|EPHA8|EPHB2|GAP43|NCKAP1L|NRG1|IL7R|ITGB7|LAMC2|STMN1|MEF2C|MSX
N2A|GRP|HPCA|KCNC3|KCND2|KCNH2|KCNJ13|KCNQ3|MEF2C|NPPA|NTSR1|PLCG2|PPP3CA|PTA
RIN2A|GRP|HPCA|IGF1|KCNC3|KCND2|KCNH2|KCNJ13|KCNQ3|MEF2C|NPPA|NTSR1|PLCG2|PPP3
K2B|GPC4|GABRB3|NRG1|TNC|IL1RAP|ITGB4|NEFL|CLDN11|PKP2|PTPRF|SLC1A1|SLC8A3|SNCG
1|GRIN2A|GRM1|GRM4|GUCY1A1|NRG1|HRH2|HTR1A|HTR4|IL1RAP|KCND2|KCNK3|KCNQ3|ME
A8|EPHB2|PTK2B|GAP43|NCKAP1L|TNC|LAMC2|STMN1|NCAM2|NEFL|NTRK1|NR4A2|PAX2|PTP
GAP43|NCKAP1L|ITGB4|LAMC2|STMN1|MYH3|NEFL|NTRK1|NR4A2|PAX2|PGM5|PMP22|PROX1|P
GAP43|NCKAP1L|LAMC2|STMN1|NEFL|NTRK1|NR4A2|PAX2|PTPRM|RET|SLIT3|SMO|TPBG|UGT8
GAP43|NCKAP1L|LAMC2|STMN1|NEFL|NTRK1|NR4A2|PAX2|PTPRM|RET|SLIT3|SMO|TPBG|UGT8
1|GRIN2A|GRM1|GRM4|GUCY1A1|HRH2|HTR1A|HTR4|IL1RAP|KCND2|KCNK3|KCNQ3|MEF2C|NT
GAP43|NCKAP1L|LAMC2|STMN1|NEFL|NTRK1|NR4A2|PAX2|PTPRM|RET|SLIT3|SMO|TPBG|UGT8
TN|PTK2B|GHSR|GRIN2A|GRM1|GUCY1A1|HRH2|HTR1A|IGF1|INHBB|KCNH2|KIT|MYBPH|NEUR
ITGB7|LAMC2|STMN1|MEF2C|NTRK1|NR4A2|PAX2|PROX1|PTPRM|RET|SLIT3|SMO|TPBG|USH2A
ES|GAP43|NCKAP1L|NRG1|CXCR1|IL16|KIT|LAMC2|NTRK1|PLAUR|PRKCD|PTAFR|PTPRM|RET|C
ES|GAP43|NCKAP1L|NRG1|CXCR1|IL16|KIT|LAMC2|NTRK1|PLAUR|PRKCD|PTAFR|PTPRM|RET|C
GAP43|NCKAP1L|LAMC2|STMN1|NEFL|NTRK1|NR4A2|PAX2|PTPRM|RET|SLIT3|SMO|TPBG|UGT8
LAMC2|STMN1|MEF2C|NTRK1|NR4A2|PAX2|PTPRM|RET|SLIT3|SMO|TPBG|WNT3|NRP1|SEMA5A
MC2|STMN1|NCAM2|NEFL|NTRK1|NR4A2|PAX2|PTPRM|RET|SLIT3|SMO|WNT3|NRP1|SEMA5A|LG
|GABRA5|GDF10|GLI1|GRIN2A|NRG1|HPCA|INHBB|KCNK3|MAS1|MEIS1|MEIS2|MSX1|NEFL|NEU
P1|CUX2|IL1RAPL2|LRRN1|CLSTN2|SEMA4A|SLITRK2|WNT3A|CBLN2|LRRTM1|LRRTM3|VSTM5
GNAT1|HPCA|HSF4|KCNK3|KIT|MEIS1|MEIS2|MIP|MSX1|NEUROD1|NHS|ROR2|PAX2|PROX1|PTPR
GRIA1|GRIA3|GRIN2A|GRM1|KCND2|KCNH2|KCNK3|KCNQ3|MEF2C|NPPA|NTSR1|PKP2|PPP3CA|P
STMN1|NTRK1|NR4A2|PAX2|PTPRM|RET|SLIT3|SMO|WNT3|NRP1|SEMA5A|LGI1|NOG|SEMA3E|P
RF|SLC1A1|SLC8A3|SNCG|PPFIA4|NRP1|WASF1|SEMA3E|GPC6|FARP1|KLK8|PDZRN3|ARC|TREM
M|RET|SLIT3|SMO|WNT3|NRP1|SEMA5A|LGI1|NOG|SEMA3E|SEMA3A|CNTN6|LHX9|SEMA4G|SEM
M|RET|SLIT3|SMO|WNT3|NRP1|SEMA5A|LGI1|NOG|SEMA3E|SEMA3A|CNTN6|LHX9|SEMA4G|SEM
4|HRH2|HTR1A|HTR4|KCND2|KCNK3|KCNQ3|MEF2C|NTSR1|PDYN|PMP22|PPP3CA|PRKCG|SLC1A
4|HRH2|HTR1A|HTR4|KCND2|KCNK3|KCNQ3|MEF2C|NTSR1|PDYN|PMP22|PPP3CA|PRKCG|SLC1A
HA7|EPHA8|EPHB2|PTK2B|FGFR1|FGFR4|GRM1|GRM4|NRG1|IGF1|IGFBP6|KIT|NPPA|NTRK1|ROR
CRHR2|GABRA3|GABRA5|GABRB3|GHSR|GIPR|GRIA1|GRIA3|GRIN2A|GRM1|GRM4|GRP|HRH2|H
GRIN2A|GRM1|HRH2|HTR4|NTRK1|NTSR1|PLCB4|PLCG2|PPP3CA|PRKCG|PTAFR|RET|RYR3|SLC8
3CA|SLIT3|SMO|TRPC3|NRP1|SEMA5A|SEMA3E|SEMA3A|RND1|SEMA4G|SEMA4A|PLXNA4|EPH

1|GRIA1|GRIA3|GRIN2A|HTR1A|HTR4|NPPA|SST|SSTR1|TIAM1|VAV3|HHIP|CREB3L1|CALML4

NTRK1|PPP3CA|PRKCG|MAPK13|CACNA1H|CACNA1G|CACNA2D2|RASGRP1|CACNG5|CACNG8
F1|IL3RA|IL7R|ITGB4|ITGB7|KIT|LAMC2|NTRK1|OSM|SPP1|THBS3|ITGA11|GNG13|CREB3L1|COL

Q3|MEF2C|NPPA|NTSR1|PKP2|PLCG2|PPP3CA|PRKG2|PRSS8|PTAFR|RAB3B|RGS4|SCN5A|CCL4|S
1|NEFL|NTRK1|NR4A2|PAX2|PROX1|PTPRM|RET|SLIT3|SMO|TPBG|UGT8|USH2A|WNT3|NRP1|W
AFR|RGS4|SCN5A|STAC|TRPC3|TWIST1|KCNAB1|KCNAB2|CACNA1H|CACNA1G|CACNA2D2|RA
3CA|PTAFR|RGS4|SCN5A|STAC|TRPC3|TWIST1|KCNAB1|NR4A3|KCNAB2|CACNA1H|CACNA1G|
|UGT8|PPFIA4|NRP1|WASF1|CLDN1|SEMA3E|NR1H4|GPC6|FARP1|KLK8|PDZRN3|ARC|TJP3|TRE
F2C|NTSR1|PDYN|PLG|PMP22|PPP3CA|PRKCG|RGS10|SLC1A1|SLC1A4|SNCG|SST|STX1A|TH|TP
RF|PTPRM|RET|SLIT3|SMO|TPBG|UGT8|WNT3|NRP1|WASF1|SEMA5A|LGI1|NOG|SEMA3E|PLPPR
PTPRM|RET|SLIT3|SMO|TPBG|UGT8|WNT3|NRP1|WASF1|SEMA5A|MYOM2|LGI1|NOG|SEMA3E|P
3|WNT3|NRP1|WASF1|SEMA5A|LGI1|NOG|SEMA3E|PLPPR4|FARP1|SEMA3A|KLK8|ARC|COBL|TS
3|WNT3|NRP1|WASF1|SEMA5A|LGI1|NOG|SEMA3E|PLPPR4|FARP1|SEMA3A|KLK8|ARC|COBL|TS
TSR1|PDYN|PLG|PMP22|PPP3CA|PRKCG|SLC1A1|SLC1A4|SNCG|SST|STX1A|TH|TPBG|DOC2A|NA
3|WNT3|NRP1|WASF1|SEMA5A|LGI1|NOG|SEMA3E|PLPPR4|FARP1|SEMA3A|KLK8|ARC|COBL|TS
OD1|NPPA|NTSR1|PDE9A|PKP2|PPP3CA|PTAFR|RGS4|SCN5A|SLC1A1|SLC8A3|STX1A|TH|THRB|
A|WNT3|NRP1|SEMA5A|LGI1|NOG|SEMA3E|PLPPR4|FARP1|SEMA3A|SIPA1L3|ARC|COBL|TSKU|
CCL4|CCL24|SLIT3|SMO|TPBG|WNT3|SCG2|NRP1|CH25H|SEMA5A|LGI1|NOG|SEMA3E|DOCK4|SE
CCL4|CCL24|SLIT3|SMO|TPBG|WNT3|SCG2|NRP1|CH25H|SEMA5A|LGI1|NOG|SEMA3E|DOCK4|SE
3|WNT3|NRP1|WASF1|SEMA5A|LGI1|NOG|SEMA3E|PLPPR4|FARP1|SEMA3A|KLK8|ARC|COBL|TS
A|LGI1|NOG|SEMA3E|PLPPR4|FARP1|SEMA3A|ARC|COBL|TSKU|CNTN6|DOCK10|LHX9|SEMA4G
GI1|NOG|SEMA3E|PLPPR4|SEMA3A|COBL|TSKU|TNFRSF21|CNTN6|LHX9|SEMA4G|SEMA4A|BC
ROD1|NR4A2|PAFAH1B3|PAX2|PCSK1|POU3F1|POU3F2|PPP3CA|PROX1|RGS4|SALL1|SCN5A|SIX

RM|RET|SIX3|SLC1A1|TFAP2B|TH|THRB|TWIST1|USH2A|WNT2|ZIC1|PXDN|NRP1|NOG|KLF4|PLP
RGS4|SCN5A|SLC8A3|STX1A|KCNAB2|CACNA1H|CACNA1G|TRDN|ARL6IP5|SCN11A|CUX2|KCN
LPPR4|SEMA3A|COBL|TSKU|CNTN6|LHX9|SEMA4G|SEMA4A|BCL11B|SLITRK2|WNT3A|PLXNA

A1|SLC1A4|SNCG|SST|STX1A|TH|TPBG|DOC2A|NAPA|CACNA1G|SLC6A5|OTOF|GPR176|KCNIP2|
A1|SLC1A4|SNCG|SST|STX1A|TH|TPBG|DOC2A|NAPA|CACNA1G|SLC6A5|OTOF|GPR176|KCNIP2|
R2|OSM|PLCG2|PPEF2|PRKCD|RET|CCL4|CCL24|DENND2B|THPO|TPBG|NRP1|RASGRP1|RAMP3|S
TR1A|HTR4|MAS1|NTS|NTSR1|P2RY2|PDYN|PLG|PTAFR|RLN1|SST|SSTR1|THRB|GABRQ|NPFFR

FRP4|SNCG|STAC|TRPC3|TWIST1|KCNAB1|KCNAB2|CACNA1H|CACNA1G|CACNA2D2|HOMER
ASF1|SEMA5A|LGI1|NOG|SEMA3E|PLPPR4|FARP1|SEMA3A|KLK8|SIPA1L3|ARC|COBL|TSKU|AR
MP3|TRDN|ARL6IP5|SCN11A|ARC|KCNG2|CACNG5|KCNIP2|KCNIP1|EHD3|FXYD6|TREM2|TESC|
CACNA2D2|RAMP3|TRDN|ARL6IP5|ARPP19|SCN11A|ARC|OPN3|KCNG2|CACNG5|KCNIP2|KCNI
M2|SDK2|DOCK10|PERP|CDH24|SVEP1|SLITRK2|IGSF21|TANC1|WNT3A|CLDN23|MPP7|CBLN2|S
3G|DOC2A|NAPA|CACNA1G|SLC6A5|OTOF|FARP1|GPR176|KCNIP2|GABRQ|GJD2|WNT3A|CBLN
4|FARP1|SEMA3A|EHD1|KLK8|NCDN|ARC|COBL|TSKU|TNFRSF21|CNTN6|DOCK10|LHX9|NYAP
LPPR4|FARP1|SEMA3A|KLK8|ARC|COBL|TSKU|CNTN6|DOCK10|LHX9|NYAP2|SEMA4G|SEMA4
KU|CNTN6|DOCK10|LHX9|NYAP2|SEMA4G|SEMA4A|BCL11B|SLITRK2|WNT3A|PLXNA4|ARHG
KU|CNTN6|DOCK10|LHX9|NYAP2|SEMA4G|SEMA4A|BCL11B|SLITRK2|WNT3A|PLXNA4|ARHG
PA|CACNA1G|SLC6A5|OTOF|FARP1|GPR176|KCNIP2|GABRQ|GJD2|CBLN2|CNIH2|SHISA6|CPLX
KU|CNTN6|DOCK10|LHX9|NYAP2|SEMA4G|SEMA4A|BCL11B|SLITRK2|WNT3A|PLXNA4|ARHG
TNNC2|TRPC3|NR4A3|CACNA1H|CACNA1G|DOCK4|SEMA3A|KLK8|SCN11A|CUX2|KCNG2|CAC
ARHGEF26|CNTN6|DOCK10|LHX9|SEMA4G|SEMA4A|BCL11B|ANTXR1|SLITRK2|WNT3A|PLXNA
MA3A|VAV3|CXCL13|CNTN6|SLAMF8|LHX9|SEMA4G|SEMA4A|BCL11B|WNT3A|PLXNA4|ARH
MA3A|VAV3|CXCL13|CNTN6|SLAMF8|LHX9|SEMA4G|SEMA4A|BCL11B|WNT3A|PLXNA4|ARH
KU|CNTN6|DOCK10|LHX9|NYAP2|SEMA4G|SEMA4A|BCL11B|SLITRK2|WNT3A|PLXNA4|ARHG

3|SLC1A1|SMO|SSTR1|TH|WNT2|WNT3|ZIC1|NAPA|NRP1|SEMA5A|NOG|TOX|ARNT2|ARPC5|SE

PR4|MAB21L2|SIPA1L3|FSCN2|TSKU|DLL1|SDK2|PLAAT1|SMOC1|BCL11B|WNT3A|UNC45B|BM
IP2|EHD3|TREM2|SCN3B|GABRQ|KCNK13|GJD2|HCN3|PIEZO2|KCNH6|HSH2D|PIP5KL1|CNIH2

EMA3A|ARL6IP5|TREM2|DOK5|LMO3|DUSP26|BMPER|MAGI3|CXCL17|ALKAL2|DUSP29|SH3RF

3|RAMP3|TRDN|ARL6IP5|SCN11A|ARC|KCNG2|CACNG5|KCNIP2|KCNIP1|EHD3|TRPV2|FXYD6|T
HGEF26|CNTN6|DOCK10|LHX9|SHROOM3|NYAP2|SEMA4G|SEMA4A|CDH24|BCL11B|ANTXR1|S
SCN3B|KCNK13|JPH1|DPP10|HCN3|CACNG8|TMEM38A|UBASH3B|GSG1L|CNIH2|STAC2|SHISA6
1|EHD3|FXYD6|TREM2|TESC|SCN3B|KCNK13|JPH1|DPP10|HCN3|CACNG8|TMEM38A|UBASH3B

2|KLHL1|SEMA4G|PRDM12|SEMA4A|BCL11B|VASH2|SLITRK2|WNT3A|PLXNA4|ARHGEF25|EPH

MA3A|SATB2|TSKU|DLL1|MACROH2A2|PCDH19|KLHL1|NEUROD6|BCL11B|FAT4|CTTNBP2|KN

REM2|TESC|SCN3B|KCNK13|JPH1|DPP10|HCN3|CACNG8|TMEM38A|UBASH3B|GSG1L|CNIH2|CR

ACR2B|STAC2|LILRA5|SHISA6
