# Supplementary material for: Transcriptomic Profiling of Electroacupuncture Regulating the Molecular Network in Hippocampus of Rats with Cerebral Ischemia-Reperfusion Injury
Source: Evid Based Complement Alternat Med. 2022 Sep 2;2022:6053106. doi: 10.1155/2022/6053106 (PMC9463016; doi:10.1155/2022/6053106)
Supplement: Supplementary Materials — Table S1: differentially expressed genes of Model/Sham group; Table S2: preliminary enrichment results of Model/Sham group; Table S3: differentially expressed genes of EA/Model group; Table S4: upregulated gene analysis; Table S5: downregulated gene analysis; Table S6: all gene analysis. [file 6053106.f1.zip › Table S3 (1).pdf]

Table S3 Differentially expressed genes of EA/Model group

Gene	log2FoldChange	FDR
LOC686143	-23.50988209	4.76E-09
Bhmt2	-10.863264	0.000761
Klrk1	-10.68317507	2.54E-05
Trim43a	-10.13191592	1.07E-08
Vom2r28	-10.08898598	0.008926
Tssk1b	-9.781756172	0.002075
LOC102549344	-9.771900677	0.000111
Spata32	-9.754618321	4.97E-06
Stpg2	-9.455751669	7.23E-06
LOC108348129	-9.269859689	0.010945
LOC108349944	-8.873052586	0.00039
LOC100909599	-8.824121361	0.00074
Olr1653	-8.427339816	0.004881
LOC102547033	-8.238424898	0.002341
Prss8	-8.175766385	2.61E-07
Prdm13	-8.010809	0.000107
LOC100364877	-7.297986772	0.000274
LOC103693456	-7.17910391	0.005962
Anxa13	-6.995536341	0.043234
LOC680624	-6.881133277	0.014101
LOC103694868	-6.880218074	0.007407
Ppp1r3e	-6.875266112	4.70E-05
Col15a1	-6.802007345	0.000915
Clec2g	-6.542403608	0.000398
Rpl30	-6.268495592	0.007613
Magea8	-6.254231472	0.000378
RGD1564409	-6.229478492	2.03E-05
LOC100909897	-6.178583513	0.000303
Serpinb10	-6.15301184	0.000636
LOC108348209	-6.104538798	0.001367
LOC103694046	-6.086089469	0.003408
Gabre	-6.034011943	0.002507
Otoa	-5.955382528	1.08E-05
Oas2	-5.91152391	4.66E-07
Osm	-5.812628589	0.000183
Adam33	-5.768924519	0.000278
LOC108348181	-5.615290776	8.92E-09
LOC102555038	-5.55817555	2.03E-05
Col8a1	-5.528924454	0.000299
Tmem74b	-5.429090047	7.22E-05
LOC100910278	-5.362717961	0.000713
LOC102552360	-5.248151976	4.14E-05
Ptchd4	-5.160149367	3.09E-05
Gbp1	-5.110796074	0.000726
LOC103692344	-5.090968203	0.017143
Erich4	-4.995213232	0.001335
LOC102553691	-4.935843616	0.021276
LOC108348048	-4.894387423	0.002313
Fam205c	-4.804092472	0.008779
Lypd5	-4.76746458	7.84E-06
Asb4	-4.736425479	0.043782
Prdm12	-4.720598227	0.001938
Cyp26a1	-4.691482899	3.58E-09
Stat4	-4.687977723	3.97E-05

Oas1e	-4.683457329	0.013364
Xkr5	-4.672597077	0.002621
Zfp773-ps1	-4.574507921	2.58E-06
Themis2	-4.50741734	0.000257
LOC100360779	-4.34697044	0.002587
RGD1310935	-4.304791758	0.010127
Shox2	-4.269185918	0.038787
LOC684773	-4.246874729	0.003123
LOC362921	-4.154485156	0.040918
Tsku	-4.137595409	0.001671
Scn11a	-4.132365816	0.001823
RGD1561730	-4.054802939	0.004468
Olr59	-4.006989612	1.56E-06
Adra2a	-3.994083649	9.78E-08
Klhdc4	-3.954020573	0.001275
Hrh2	-3.934585977	0.017965
Atp10b	-3.901925049	0.001676
LOC100910068	-3.860626596	0.000166
Ly6k	-3.860626596	0.000166
Tet1	-3.760864791	6.23E-05
Adra1b	-3.725799188	6.46E-09
LOC108349608	-3.713966864	0.004789
Prr20e	-3.693250265	0.015481
RGD1561231	-3.665553518	0.002255
Allc	-3.655678662	0.001984
Smco4	-3.62974338	0.004343
Socs6	-3.628196004	0.001138
Rln1	-3.602815609	0.002579
Zfp36l3	-3.540581022	0.00051
LOC108350654	-3.533961145	0.010695
Gkn3	-3.499696971	0.006215
Trim54	-3.497440268	0.000246
LOC103693051	-3.470472603	0.005978
Rd3l	-3.458942265	0.009084
Prg2	-3.454430473	0.002682
Cxcl13	-3.452129825	0.002467
Ddx43	-3.433781877	0.034253
Crhr2	-3.378296859	1.82E-06
Enthd1	-3.376983121	0.018215
Col24a1	-3.337268661	0.000131
RGD1561143	-3.315247309	0.041611
Exoc1l	-3.30448818	7.80E-05
LOC680316	-3.302457589	0.01001
Gml	-3.286653251	0.008753
Gimap9	-3.264566196	5.93E-06
Plcg2	-3.255966913	0.00019
LOC103689995	-3.194741814	7.59E-11
LOC100361265	-3.153042311	0.024631
Fcrl2	-3.137973561	0.042544
Ripor3	-3.136630782	1.87E-05
Cd247	-3.131716591	1.06E-05
Slc39a6	-3.114309799	0.005629
Fuom	-3.10635086	5.30E-09
Vill	-3.062010377	0.00045
LOC103690131	-3.041825104	0.006075
Rph3al	-3.0232405	0.016334

Ret	-3.007660193	0.000299
Klhl1	-2.991534836	3.11E-07
Lrrc19	-2.964491681	3.10E-05
Scn5a	-2.924325808	0.004987
Car10	-2.873907767	0.000122
Mapk13	-2.866629556	6.84E-11
LOC103692936	-2.861906337	0.000548
Samsn1	-2.861359678	0.005166
LOC100911278	-2.820682457	0.00303
LOC298795	-2.809849269	0.041516
Fap	-2.804677843	0.019048
Dpp4	-2.789756584	0.00175
Itgb7	-2.767212363	5.68E-05
LOC103692555	-2.75047017	3.52E-05
Cdpf1	-2.745421008	0.001594
Tmem196	-2.742633437	4.34E-08
Cd226	-2.722945389	0.003574
Nags	-2.722094191	0.018807
Twist2	-2.721059914	0.010357
Sh2d7	-2.713331007	0.000837
Tcerg1l	-2.70572344	6.66E-08
Rasgef1c	-2.666891744	7.87E-06
Ltb	-2.646222452	0.004792
Cyp11b2	-2.639129071	0.000566
Slc16a12	-2.629812735	0.000615
Gabrq	-2.628676419	0.026759
Spint3	-2.614051618	0.002604
Trdn	-2.602000994	2.28E-07
Lgals7	-2.599955447	0.000553
LOC102546864	-2.596092905	0.007975
Pcdh11x	-2.590702114	3.94E-05
Ms4a6bl	-2.576053793	0.025769
P4ha3	-2.565869023	9.61E-15
Igfn1	-2.563888958	9.95E-05
Myl4	-2.5513648	0.011583
Cbln2	-2.540939676	0.00051
Fibin	-2.523896977	0.009785
Batf3	-2.521025909	0.000417
Ermap	-2.511148229	0.037326
Glra1	-2.509801986	0.042454
Sema3a	-2.508645649	1.08E-06
Cep295nl	-2.50741232	7.58E-12
Rexo5	-2.493818478	0.003056
Zic4	-2.489793193	0.007121
LOC102551847	-2.486145355	0.001298
Lhx3	-2.481923432	0.017447
Ppp1r17	-2.470988608	0.016612
Ocm2	-2.4699614	0.019546
LOC100910678	-2.469737649	0.033255
Ust5r	-2.453130504	0.041974
Tdrp	-2.451791792	0.00303
Plcxd3	-2.428727893	0.015192
Lamc2	-2.427155747	1.49E-05
Cux2	-2.416924609	3.14E-07
Gcdh	-2.415231048	0.000735
LOC300308	-2.402598664	1.47E-10

Taf11	-2.400284396	0.001938
LOC108352751	-2.390899188	0.007978
Yju2	-2.385931351	0.004927
Loxl2	-2.384734405	0.004455
Nr1h4	-2.382852777	2.96E-05
Camk2d	-2.373834746	5.71E-42
Gkn2	-2.364050902	0.000449
LOC102546678	-2.363269003	2.53E-07
LOC108348429	-2.354463166	5.36E-08
LOC102555476	-2.350022961	0.006306
Gfi1	-2.342214985	0.000107
RGD1565071	-2.335569942	1.84E-06
Ccl9	-2.332792915	0.000393
Rprm	-2.32311736	1.02E-06
Tekt4	-2.319111874	0.012754
Fhod3	-2.307311484	4.76E-10
Efna5	-2.30657333	1.04E-05
Il1rapl2	-2.303920548	0.007807
Ovol2	-2.29604562	0.021037
Atp2b4	-2.294898147	2.06E-09
Calca	-2.286619823	0.001813
LOC100911326	-2.283618704	0.02471
Srpk3	-2.277587657	9.76E-07
Pmfbp1	-2.2756349	0.001507
Col6a3	-2.268795267	2.47E-06
Ano2	-2.267971168	0.046361
LOC108348215	-2.265293603	0.011495
Dpt	-2.265227246	0.016023
LOC102551559	-2.260047346	0.000128
Hs3st2	-2.248545146	1.47E-09
Bhmt	-2.242829954	1.08E-09
Vstm5	-2.238318675	0.020018
Gnrh1	-2.235119463	0.031203
Tnnc2	-2.233812587	4.78E-07
LOC103693257	-2.229663409	0.003867
Cox6a2	-2.22465635	0.039892
Clcnka	-2.179697573	0.004531
Fam205a	-2.170393049	0.041393
Hjv	-2.170259171	0.040918
Plg	-2.163388045	0.029659
Dmgdh	-2.158019469	0.024965
Bmper	-2.157747246	2.74E-07
LOC108351576	-2.154721161	1.23E-06
Zic1	-2.148903216	0.003855
LOC108348336	-2.128499175	0.018499
Catsperz	-2.122291902	0.000173
Mospd4	-2.103428851	0.048343
Mybpc1	-2.097232454	0.037912
Slc23a3	-2.092387158	9.74E-07
Psma8	-2.091546441	0.002804
Crhr1	-2.089362237	6.66E-08
Mael	-2.082481019	0.042811
Col9a1	-2.07731871	5.16E-08
Slfn4	-2.062652091	0.009104
Aunip	-2.058165241	0.044104
Gtse1	-2.052241236	0.030005

Rsl1	-2.044391526	0.032169
Ipcef1	-2.038894013	3.34E-05
Mef2c	-2.037970269	7.29E-09
LOC102555341	-2.035198664	0.000264
Lilra5	-2.032391655	0.009584
Shisa3	-2.027393548	0.006706
LOC100911537	-2.016960567	0.030372
Npffr1	-2.010643996	0.000356
RGD1561662	-2.008543276	0.000227
Best3	-2.008299005	0.004084
Hs3st5	-1.989483388	0.021426
Slc17a4	-1.984955376	0.00334
Cdc6	-1.98167677	0.007259
Gpr15	-1.973393389	4.31E-07
Cbln3	-1.972122314	1.34E-05
LOC100911699	-1.971435637	5.43E-10
Barx2	-1.968518984	0.03497
Stx1a	-1.96743401	4.27E-05
Gpx3	-1.964248315	5.57E-10
LOC100909715	-1.963102049	0.02993
Erich6b	-1.962464452	1.05E-13
Gnb3	-1.961804398	0.003646
Nphs2	-1.961126915	0.002501
Chrna1	-1.955298282	2.36E-05
LOC108352191	-1.943548428	1.00E-05
Adprhl1	-1.937856839	0.03497
Satb2	-1.936353548	0.012436
LOC684998	-1.932448602	0.000194
Mas1l	-1.931491168	0.004021
Galns	-1.927709795	3.27E-08
LOC103691893	-1.925626665	0.013162
LOC102554130	-1.921423855	0.000991
Lin7a	-1.915738129	0.001123
Atp6v1b1	-1.914077785	0.023096
Adamtsl2	-1.903822493	1.58E-20
Zic3	-1.895893837	0.001821
Pstpip1	-1.894324391	0.002546
Ttc22	-1.891926398	1.31E-07
Vom2r52	-1.891201862	0.000147
LOC108349482	-1.885462583	2.39E-05
Dpp10	-1.883001918	3.13E-14
Nppa	-1.882569268	0.000391
Xlr3a	-1.87590092	0.019932
LOC103690508	-1.87518967	0.000264
Grid2ip	-1.87417134	0.001955
Npr3	-1.8691229	0.017606
Slfn14	-1.865644005	0.043852
Nle1	-1.857850627	0.013563
LOC103694522	-1.855328292	0.020232
LOC103691272	-1.854227777	8.27E-05
Gpr153	-1.852349674	0.000275
Cxcl6	-1.840252937	0.010018
Gckr	-1.836782081	0.023318
Dkk1	-1.836061236	0.02161
Ccdc38	-1.835531771	0.046276
Chrna4	-1.831594816	0.000793

Plek2	-1.822697072	0.004468
Cntd1	-1.821964011	3.68E-11
Smim24	-1.820867506	0.000247
RGD1566368	-1.816270924	4.55E-07
Car5a	-1.815431387	6.54E-10
Psmb11	-1.814044302	8.57E-06
Rwdd2a	-1.798110384	1.73E-05
Mnda	-1.797782432	0.000117
LOC108348167	-1.795742507	6.16E-09
C8g	-1.786150698	0.028669
Ccl24	-1.782810194	3.56E-05
Sfrp5	-1.782752172	0.000717
LOC103690039	-1.778175862	2.82E-05
LOC108350822	-1.771665428	0.001731
LOC100364265	-1.769881392	3.02E-07
Dhh	-1.769691425	4.19E-06
Steap3	-1.762616074	0.002198
N5	-1.761048882	2.63E-08
LOC100912904	-1.759664764	4.33E-07
Il36rn	-1.754272044	0.011109
LOC103691072	-1.75243437	0.010427
Prok1	-1.742980454	0.011403
Plekhm3	-1.742938943	0.032601
LOC108349071	-1.738357599	0.034253
Cyp2c6v1	-1.731492519	0.034893
LOC499219	-1.728254099	3.83E-07
Cmtm4	-1.727589268	0.035312
RGD1565117	-1.72509121	0.000242
Camk2n1	-1.722750012	0.000841
Gfra2	-1.720389778	1.45E-08
Piwil1	-1.720043045	8.26E-10
Gsdma	-1.719382097	1.20E-09
RGD1566251	-1.715212741	1.05E-05
LOC108351580	-1.713177109	0.011399
LOC103693202	-1.711206722	9.70E-06
Mixl1	-1.704290883	0.001576
Blk	-1.703459417	0.00737
Dlx4	-1.697094324	0.004315
Nkx2-1	-1.692618945	0.023907
Tm2d3	-1.687713779	0.02732
Tubd1	-1.684876834	0.000821
Cdh7	-1.683343732	1.64E-05
Pklr	-1.681567504	0.003545
Sh2b2	-1.680210299	0.000151
Tent5c	-1.679895976	8.50E-05
Rhod	-1.677292976	0.009625
Akr1d1	-1.676613666	4.66E-22
Cck	-1.674059493	0.001593
Cntnap5b	-1.673812861	0.001314
Plekhg6	-1.673281721	0.000579
Zik1	-1.671974902	0.048641
LOC102551633	-1.671444252	0.037732
Dach2	-1.671253787	8.38E-08
LOC103690779	-1.670592675	2.56E-05
Ramp3	-1.665803045	0.005235
Cplx3	-1.665465638	4.79E-06

Tpbg	-1.662455269	6.78E-06
Doc2a	-1.65591803	0.000309
Stard8	-1.653074006	0.000518
LOC103693723	-1.647880339	2.99E-05
Tmem269	-1.645838515	0.017005
Cyp4a8	-1.637869603	0.000491
Lrit2	-1.631494508	2.11E-15
Rassf7	-1.629310848	0.00196
Efcab6	-1.627106784	6.98E-05
Ctu2	-1.624477798	0.010541
Ireb2	-1.622828529	0.019533
Spag4	-1.622604907	0.000568
Slc5a3	-1.621622414	0.011048
RGD1561149	-1.619261518	0.002964
Plcb4	-1.618856036	5.33E-05
Cnr2	-1.618745636	5.82E-06
LOC108349536	-1.617143507	1.15E-06
Adam30	-1.616626095	0.011846
Garnl3	-1.614518721	1.02E-06
LOC365238	-1.61290839	6.23E-08
LOC100910885	-1.611996369	0.000174
Mpl	-1.610529548	1.94E-05
Catsperg	-1.610313624	3.56E-05
Tfap2b	-1.608582707	0.038058
Otof	-1.608538076	0.009522
Cpne9	-1.606245722	0.023414
LOC108352465	-1.593420173	0.000677
Ubxn7	-1.593256509	0.000963
Fbxw10	-1.591734344	0.000646
Ifi27l2b	-1.591184874	0.00674
LOC499796	-1.58824001	0.002653
LOC102555814	-1.587744462	0.000349
Gfi1b	-1.587017818	0.03273
Myom3	-1.585258631	0.000222
Clnk	-1.585225671	0.005819
LOC102554611	-1.584778004	0.034679
Hsd17b1	-1.583601646	0.008308
Mip	-1.583117134	1.13E-10
Tgm4	-1.575365706	0.001335
Vwa7	-1.5747262	7.69E-06
Nmur1	-1.572335862	0.005905
LOC500354	-1.572166661	8.06E-05
LOC103692848	-1.570638255	6.62E-07
LOC103689949	-1.568943264	0.000706
Mia	-1.568423732	0.024134
LOC292543	-1.564100602	0.019971
Ccnb1ip1	-1.561821797	0.000197
Scx	-1.561492072	0.015234
Olfm2	-1.560311549	0.000659
Tspan1	-1.559786385	0.000445
LOC680227	-1.553608793	3.95E-17
LOC108352109	-1.552188648	0.017975
Gcat	-1.547982109	9.97E-06
Fyb2	-1.546442962	5.82E-08
LOC100910079	-1.543037199	0.014495
Nanog	-1.541830734	0.012548

Ptpn14	-1.539948179	9.02E-08
Gcsam	-1.538852794	0.023421
Asb14	-1.538820356	0.00221
Gng13	-1.538500679	0.000201
Hrasls	-1.537181084	0.016031
Rassf5	-1.53718053	9.81E-05
LOC303448	-1.533604869	5.28E-24
Lefty2	-1.531147919	0.035979
Slc10a2	-1.527609872	2.70E-16
Runx1	-1.52675587	0.000346
Clec2e	-1.521137294	2.59E-05
Pitpnm3	-1.518294152	0.006979
Oosp1	-1.512115193	0.014693
Upk2	-1.511659603	0.000515
Igsf21	-1.50792274	6.13E-12
Cyp17a1	-1.507672688	0.037559
Hpcal1	-1.507511012	3.83E-24
LOC361914	-1.502523609	0.005116
Rgs4	-1.500649794	0.022459
Muc6	-1.500039036	0.049938
Usp35	-1.498547388	1.11E-24
LOC680077	-1.498378958	2.13E-06
LOC102551901	-1.497088609	0.005593
Stac2	-1.493669833	0.002496
Flt3	-1.493049293	0.013633
Ttc39a	-1.487139797	2.03E-06
Cars2	-1.485881197	0.020612
LOC102548478	-1.483140592	2.17E-08
LOC100363112	-1.481914667	0.010191
Pnpla3	-1.4729624	3.70E-05
Naip5	-1.466760314	1.71E-05
Pla2g2d	-1.465760608	0.011794
Dusp26	-1.464635557	1.59E-06
LOC103690170	-1.461386792	0.01427
RGD1305298	-1.461386792	0.01427
LOC108350576	-1.457349387	9.54E-09
Gcm2	-1.45491453	3.26E-07
Adap2	-1.454845176	0.000783
LOC499565	-1.453305418	0.041528
LOC103690158	-1.445895339	0.028959
LOC100363314	-1.445036128	0.000206
Scgb2a2	-1.443359621	0.005834
LOC108352957	-1.442291465	0.008308
Scube1	-1.441428351	0.00983
Epop	-1.437320538	0.018171
LOC103692792	-1.436106352	1.41E-07
Tshz3	-1.433965991	0.007193
Nhs	-1.433537732	0.00411
Ppef2	-1.433530833	0.00473
Cideb	-1.425380265	0.003545
Harbi1	-1.422971198	5.34E-06
Hmcn2	-1.421830841	0.003446
Gltpd2	-1.418091462	0.041445
Hsf4	-1.416250785	2.56E-07
Draxin	-1.415197655	0.011794
Asb5	-1.414364635	0.019216

Ncr1	-1.412556307	0.000117
LOC499469	-1.410894678	0.000591
Ephx4	-1.405921239	0.00144
RGD1563056	-1.40021688	0.004378
Birc3	-1.399362588	0.017174
Slc41a2	-1.399075315	0.00033
Igf1	-1.397710071	0.003589
Tmem202	-1.39680116	3.99E-06
LOC501396	-1.396004536	0.000648
Msr1	-1.387172423	0.012233
Trpv2	-1.387003553	2.15E-13
LOC108353290	-1.386543709	0.002277
C2cd4c	-1.384655458	7.21E-06
LOC691414	-1.383832382	0.001176
Trim31	-1.382526879	4.81E-05
LOC100911109	-1.379581001	0.000257
P2ry2	-1.37865389	0.019533
Dbndd1	-1.378548242	2.82E-05
Mxra7	-1.375718209	1.14E-06
Trpm8	-1.37217523	0.000283
Fam131c	-1.371454385	2.61E-05
Nlrp1a	-1.371392576	7.90E-14
LOC100911993	-1.370492419	0.000114
RGD1309139	-1.370286071	1.81E-06
Dnajb7	-1.368316725	0.029093
Zfp558	-1.365441694	1.47E-09
Slc25a52	-1.36535203	0.000739
LOC102552911	-1.364224371	0.008998
Adgrd1	-1.363493699	0.012116
Postn	-1.359080826	0.008539
Plac8l1	-1.354373477	0.035984
Cd40	-1.348270339	7.68E-05
LOC302192	-1.345349234	0.033906
Lgr5	-1.345301418	0.015248
Efcc1	-1.343465426	0.036029
Nog	-1.343324336	6.75E-06
Cabp1	-1.342958837	0.022443
Rbm47	-1.340532744	1.91E-07
Ccm2l	-1.340141381	0.000115
MGC114483	-1.339503604	1.50E-06
Rnd1	-1.337434249	0.036247
Tgif1	-1.336871717	0.044818
Tlr6	-1.336796226	1.31E-08
Egfem1	-1.330350246	9.93E-05
Lyve1	-1.326597786	0.00046
LOC100363129	-1.325888033	0.032789
Ptgfrn	-1.325861017	0.034353
Gstt2	-1.323551694	0.000495
Cacna1g	-1.323301721	0.000918
Med12l	-1.322559261	8.74E-09
LOC500846	-1.317793532	0.013247
LOC102555598	-1.312926184	0.006306
LOC499742	-1.312159693	7.26E-10
LOC103694875	-1.311819335	0.030099
Fpr1	-1.310079137	0.006548
Rasgef1b	-1.309348639	0.026511

Nat8f3	-1.307778072	0.024631
Kcnk3	-1.305980063	0.005547
Zic2	-1.305909087	0.032753
Liph	-1.305129029	0.018684
Suv39h2	-1.301112578	0.046023
LOC102547060	-1.299584283	4.35E-06
Hsd3b7	-1.296575539	0.04117
Zfhx4	-1.295624606	0.000165
Pip5kl1	-1.289605727	8.17E-06
Wisp2	-1.288041314	0.009841
Abcg3l1	-1.279299102	2.10E-09
Art2b	-1.277854326	0.04008
LOC108352688	-1.276631403	0.000417
Cdc14b	-1.273634451	1.28E-08
N4bp3	-1.267599774	4.26E-05
Cd3eap	-1.266032203	0.001805
Rtbdn	-1.264591837	0.00051
LOC108353239	-1.26308019	1.50E-09
Tpd52l3	-1.263045204	0.003745
Siglec1	-1.260589804	0.005816
Tnfaip8l2	-1.258159788	0.016169
Chtf18	-1.25769973	0.026446
RGD1310212	-1.256129744	4.14E-05
LOC102550396	-1.254923553	1.23E-10
Gipr	-1.254285446	0.001731
Sh3bgrl2	-1.253811778	0.010277
Igfbp6	-1.252122155	0.023096
Slc24a5	-1.249764292	0.032747
Ptprt	-1.246472428	4.21E-05
Lin28a	-1.244142637	0.03141
Lin28b	-1.24344867	0.049362
Il1rn	-1.23377233	0.014964
Oasl2	-1.232666763	0.03421
Clcnkb	-1.232189269	0.007479
Gpr82	-1.231456474	0.01279
Treml1	-1.23037682	0.019583
LOC108350921	-1.229414778	0.00023
Vgll3	-1.229163234	0.008425
Bpifc	-1.229160914	0.031922
LOC500035	-1.227898479	1.01E-07
Tmie	-1.225719822	3.29E-05
Pcbd1	-1.225480883	0.000373
LOC102549174	-1.220323126	0.032789
Btbd11	-1.219785937	0.000596
Dhrs7l1	-1.219121239	4.03E-07
Prph	-1.217767789	0.013014
Gemin6	-1.215599133	0.000117
Pou6f2	-1.213538614	0.001072
Prkcd	-1.212340893	0.011444
Ugt2b17	-1.211850687	0.011413
Dok5	-1.208578111	5.69E-09
LOC499843	-1.206856098	0.000193
Elac1	-1.205475804	0.030284
Kcnh6	-1.201219422	0.001086
LOC102551311	-1.200267925	3.69E-11
Grip2	-1.200203708	1.24E-12

Tssk3	-1.197834949	0.040554
Sync	-1.196837529	9.09E-05
Cldn15	-1.195413893	0.000153
Ybey	-1.192135249	2.93E-07
Cdhr3	-1.191178281	0.009991
Coro2a	-1.189721454	2.92E-05
Dact2	-1.189712367	0.028866
Mreg	-1.189400847	0.030059
Pou3f2	-1.18698803	0.017721
Gng4	-1.185932988	0.000372
LOC108350133	-1.18582785	0.000246
Socs2	-1.182821293	0.000824
Ftcd	-1.181348179	0.020723
Naglt1	-1.176931314	4.22E-10
Insyn2b	-1.172675899	3.26E-11
Optc	-1.17144821	0.000247
Rbm41	-1.171042478	0.006374
Sh3rf3	-1.170834247	5.40E-07
Tmem145	-1.170652646	0.000528
Lrrtm3	-1.16240603	2.08E-05
Zic5	-1.161527426	0.030277
Fam83f	-1.158989846	0.047756
Ascl2	-1.158202842	0.014101
Fkbp11	-1.154570586	7.13E-06
RGD1563482	-1.152235115	5.37E-05
Nlrp3	-1.150020612	0.000314
Cit	-1.144825656	0.01444
Pou2f2	-1.142566049	6.63E-05
LOC503053	-1.13809155	3.88E-06
LOC100361645	-1.13713031	2.05E-08
Ms4a6b	-1.13646975	5.46E-06
Zyg11a	-1.136050754	0.002651
LOC102550530	-1.134435864	0.001331
Lzts1	-1.132646346	0.001164
Armc7	-1.131879028	2.39E-05
Pcdh7	-1.131744042	0.027262
Col2a1	-1.129879816	0.043374
Mapk12	-1.128748147	0.002222
Rock1	-1.127845519	2.14E-10
LOC102547753	-1.12757505	0.001851
Gap43	-1.127320092	6.56E-07
Tbx10	-1.124899061	0.033367
LOC102555942	-1.123443711	0.018011
Hrc	-1.121453079	0.025093
Ssh1	-1.118684996	1.08E-05
Cybrd1	-1.117890171	1.56E-05
LOC100361655	-1.117128446	0.00325
Nrg1	-1.116495888	3.29E-07
Elavl4	-1.115447238	0.001585
Lipogenin	-1.114621969	4.99E-05
Prrg3	-1.110953579	0.001415
Alb	-1.110487105	0.030217
Fam92b	-1.109327676	0.000554
Cdh18	-1.10902116	0.046872
Prcd	-1.108803688	0.02471
Slco1b2	-1.105784748	0.006559

Slc7a3	-1.104742431	0.00242
Chrna5	-1.104581788	0.018583
Trim45	-1.103856626	0.000568
Sfxn2	-1.100474102	0.000415
LOC100910478	-1.100150133	0.049525
Thrb	-1.097661274	4.76E-10
LOC317165	-1.094663474	2.34E-08
Smoc1	-1.090570887	0.017721
Acr	-1.090038027	0.004624
LOC24906	-1.086521161	2.77E-07
LOC102548541	-1.086437584	1.69E-06
Th	-1.085211847	0.014198
LOC691254	-1.083580134	2.86E-10
LOC108351541	-1.080216884	7.68E-05
Hacd4	-1.079507072	0.000571
Ints7	-1.076874088	0.003956
LOC499240	-1.075551898	0.020786
Chek2	-1.075009513	0.015123
RGD1560556	-1.074644544	0.000116
LOC685668	-1.072616766	0.036917
Arr3	-1.069956836	9.83E-05
Htr2b	-1.065606898	0.047195
Cyp4f17	-1.064186283	5.73E-06
Zfp90	-1.059928978	1.52E-09
Cdk15	-1.05619681	0.00665
Coro6	-1.055186486	0.009082
Cldn20	-1.053407345	0.020662
Lmbrd2	-1.052281602	0.000592
Lrrn4cl	-1.051480924	0.011878
LOC498084	-1.051313765	0.038378
LOC102547626	-1.050027549	0.001206
B3gntl1	-1.049703789	0.002604
LOC499136	-1.045442701	2.14E-10
LOC108348074	-1.044903984	0.000275
Pcdhga11	-1.043480112	0.0002
Mapk11	-1.042939632	0.043242
Slc5a5	-1.042891964	9.58E-08
Tmc5	-1.042395187	0.000334
Cds2	-1.040199899	0.006825
Atp6v1c2	-1.039488992	1.52E-09
Pdzrn3	-1.03610843	0.000149
Ctla2a	-1.035444598	0.028888
Dleu7	-1.030639219	0.00176
Ahnak2	-1.029674953	7.85E-05
Slfn5	-1.029021984	0.021916
LOC681367	-1.02787073	6.20E-07
Ly6g5b	-1.026210614	0.020018
Rgs17	-1.021162408	4.85E-11
LOC102549812	-1.019304455	0.000422
Kcnip1	-1.01800438	1.20E-05
Cabyr	-1.015100515	0.020487
Il3ra	-1.014759713	9.09E-05
Cd14	-1.010994909	0.039655
Klf6	-1.009639244	1.46E-12
Megf11	-1.008961614	0.000971
Ankrd39	-1.006873681	1.48E-05

Milr1	-1.005045228	0.013506
Sst	-1.003846942	1.66E-12
Pde9a	-1.00145288	0.003149
Farp1	-1.000288288	3.81E-08
Nkap	-1.000175555	0.022747
Gab1	1.001462614	0.00448
Ddo	1.001875948	0.003293
Slc30a10	1.003194889	0.035065
Notch2	1.003328376	0.000391
Inpp5b	1.004926511	6.23E-05
Ptpre	1.009977787	0.010829
Ptk2b	1.010052722	0.003957
Golt1b	1.012000165	0.020879
Pllp	1.012288486	2.54E-05
Ryr3	1.01338854	5.20E-05
Serhl2	1.01443165	0.011889
Tecta	1.015585764	0.029346
Ceacam1	1.017831407	0.002793
Mcam	1.019525356	2.59E-05
Cttn	1.021359269	6.08E-05
Slit3	1.021510815	0.00492
Ldb3	1.024194378	0.021235
Adgrb3	1.024551349	0.006559
LOC100362819	1.026804204	8.87E-09
Dhx33	1.026836706	0.02616
Lmo2	1.027035499	0.021209
Rnf150	1.027578548	0.043017
Dll1	1.027638299	0.001731
Frat1	1.028089792	0.000695
Gnb4	1.028342612	0.001263
Gpr37	1.029609682	0.000824
Raver2	1.032289172	0.002477
Erbb3	1.032873902	0.011709
Ccdc71l	1.033993426	0.022747
Kndc1	1.034900873	0.00831
RGD1310819	1.034963341	0.000161
Spcs3	1.035277799	0.033219
Gpr62	1.03606381	0.00557
Camk1d	1.038940973	4.67E-06
Dph1	1.042740397	5.79E-08
Zfp385b	1.045552521	4.91E-05
Slc16a11	1.046455521	0.026511
Arid1b	1.0468246	4.35E-05
Slitrk2	1.048213003	0.032813
Ppp4r4	1.04883736	1.71E-07
Evi2a	1.049887533	0.009641
LOC100363494	1.051802171	3.34E-05
Pbld1	1.052408878	0.014842
Rgs5	1.054039881	0.020232
Gjc2	1.058118208	0.011147
Mybph	1.059797174	3.97E-05
Extl3	1.060895208	0.001362
Bcl6	1.061613455	0.015632
Hist1h1c	1.06195518	0.027295
Grik2	1.063049824	0.000712
Tob2	1.06763898	0.025799

Scarb2	1.068079981	1.51E-06
Flrt3	1.068097445	0.000295
Nhej1	1.068640102	0.044051
Ahcyl2	1.068820034	0.001081
Slc35d1	1.06986737	0.005232
Chm	1.072926019	0.000393
Tf	1.072995713	0.003374
Apod	1.074322668	0.003427
Mob1b	1.074382808	0.037431
Pcyox1	1.077434863	0.000301
Chrna2	1.078674775	0.03583
Slc38a10	1.081368628	0.043799
Nefl	1.083226669	0.044104
Kbtbd11	1.083293191	3.54E-05
Exoc8	1.084212436	0.008641
Cdo1	1.085141815	0.008995
Sipa1l3	1.086232619	0.002141
Gpr27	1.094037277	0.00381
Cotl1	1.09552795	0.018312
Rffl	1.097831113	0.000942
Thbs3	1.098216288	1.73E-07
Ankle2	1.098925137	0.000697
Car12	1.099937986	4.06E-08
Tmem63a	1.1010117	0.001053
Tmem18	1.103278881	0.036818
LOC100911951	1.103837571	2.65E-14
Sh2d5	1.104643477	2.52E-06
Frmd4b	1.105468849	4.74E-08
Fam155b	1.106328532	0.00334
Rrn3	1.106769181	0.000269
Kpna4	1.109379738	4.53E-07
Cyp2j10	1.114244527	0.039444
Vps50	1.11571651	4.32E-08
Sumo4	1.120193612	0.001627
Tomm70	1.120295357	0.001373
Sgpp2	1.120552045	0.009398
Pik3c2b	1.121332811	0.014693
Fscn2	1.125062452	0.011272
Ddx17	1.126715801	6.14E-05
Scamp5	1.127284609	0.009058
LOC314407	1.127501601	0.00584
Plppr4	1.127899685	0.014235
Dcp1a	1.128723674	0.045381
Slc44a5	1.135259468	0.041635
Cadm2	1.137781398	0.015559
Serinc5	1.138267435	4.10E-06
Tgfa	1.139134676	4.54E-06
Fn3krp	1.139320515	1.76E-10
Ppfia4	1.139443404	1.93E-11
Hs3st1	1.142467063	0.002575
Ankrd36	1.143913482	0.02023
LOC103690006	1.143923316	8.66E-06
Pcdh1	1.146219412	0.024236
Ppil6	1.146847788	0.006526
Tmcc3	1.147040967	6.43E-07
Efna2	1.147816622	0.019781

Tagln	1.149407567	0.00048
Clptm1l	1.150262318	0.013834
Cttnbp2	1.153259939	5.23E-11
Cpne6	1.15472684	0.029097
LOC100361087	1.155179933	0.019533
Epha6	1.161898772	0.015206
Sema3d	1.162944888	0.038233
Mog	1.164575346	0.000174
Astn2	1.165732792	0.000366
Arl15	1.166137658	7.11E-06
Galnt6	1.168270472	0.00024
Tmem38a	1.16894851	8.88E-06
RGD1563354	1.173826391	1.73E-10
Dusp6	1.176555541	0.002933
Slc1a1	1.177086732	0.002464
Smcr8	1.1816417	0.026597
Cbx4	1.182911899	4.62E-08
Scg2	1.183446163	0.002807
Mctp1	1.184399195	0.002666
Grm1	1.185356591	1.14E-07
Mast4	1.188387739	0.000198
Opalin	1.189183652	0.001593
Bcl11b	1.190322905	0.005286
Cecr2	1.191294505	0.000171
Pxdc1	1.192181873	0.000514
Zfpm2	1.195739067	0.00584
Lgi1	1.197343838	0.013523
Gli2	1.197868015	0.008548
Cnp	1.199882302	0.000245
Plp1	1.203878019	0.009146
Rcn3	1.205227248	0.002752
Acan	1.205890951	0.013563
Inf2	1.207766157	0.005903
Msra	1.207782747	0.001165
Ninj2	1.211777882	0.002313
Il1rap	1.220577584	0.013728
Homer3	1.222357585	0.001206
Galnt18	1.223640982	0.000695
Cdc40	1.224762481	0.000665
Glcci1	1.231757422	0.00033
Epha7	1.234604131	0.017901
Fanci	1.240303744	0.000141
Hs3st4	1.245097057	0.001616
Grm5	1.251508917	0.008311
Tprkb	1.252474976	0.020662
Plxdc1	1.252655206	1.24E-05
LOC100910957	1.252669937	0.000432
Prpf4	1.254610646	0.001278
Sema5a	1.261692345	0.017307
Pdgfb	1.26620676	0.029671
Zfp804a	1.268886151	0.004624
Pxylp1	1.271771725	0.000841
Arl6ip5	1.27356554	0.001054
Dcc	1.273931704	3.59E-09
Chtopl1	1.284341647	3.20E-10
Abca8a	1.285164442	0.001005

Zswim5	1.286201864	5.21E-13
Gpr17	1.28720733	1.01E-07
Cebpd	1.306933212	4.40E-05
Csmd3	1.307348033	0.008322
Cebpb	1.308267454	0.002407
Ddr1	1.309014281	0.000177
Scn3b	1.310297247	0.001268
Egr1	1.314434934	0.004514
Anln	1.31954022	0.008948
Cdc42bpg	1.325574487	0.001304
Nkx6-2	1.328793355	0.000398
St8sia1	1.34005734	0.023404
Chn2	1.355475923	0.000181
Greb1l	1.359612366	0.031074
Gpr155	1.361010951	4.76E-10
Dpf3	1.364787689	0.011673
Ankrd52	1.369476631	0.003394
Tbc1d8b	1.37753196	8.43E-05
Mal	1.377761026	0.006536
Kdm4c	1.381841199	4.76E-07
Cacng8	1.393612212	0.000815
Myom2	1.399244342	0.011109
Kank4	1.399931651	0.002171
Ugt8	1.401574571	0.000726
Tbc1d16	1.402415131	0.001488
Clstn2	1.402857781	0.026511
LOC100361898	1.403366952	0.041528
Msx1	1.405273888	1.36E-06
Ror2	1.412684152	0.003475
Scube2	1.412767173	0.0023
Zfp483	1.415591302	0.013888
Slc6a20	1.418024314	0.013414
Mag	1.418406199	5.28E-05
Mat2a	1.421857338	9.93E-05
Ankdd1a	1.425764225	3.88E-05
Ephb2	1.425916362	0.03114
Esyt3	1.426049386	6.02E-06
Ano3	1.429301008	0.012782
Gabra5	1.430896735	0.02703
Tjp3	1.433086739	0.002679
Stk26	1.438550019	0.00031
Rsph10b	1.440419531	0.008713
Fkbp9	1.440673879	1.81E-11
Kcnab1	1.442915671	4.76E-10
Ncbp2	1.443436586	0.040819
Gpr12	1.447274056	7.90E-14
Selenov	1.453155284	0.016556
LOC100910275	1.458494886	0.003826
B3galt5	1.46056038	1.03E-07
Gjb1	1.462672835	0.000872
Strip2	1.462966372	1.32E-07
Gria1	1.465244154	2.39E-06
Dsp	1.470672701	0.002767
Gabrb3	1.474810881	0.001108
Pmp22	1.476998263	1.68E-07
LOC108349154	1.477611838	0.004949

Pwwp2b	1.478739602	4.89E-07
Rgs10	1.478768579	0.002198
Fa2h	1.478987946	0.00023
Ptp4a1	1.485626108	1.67E-05
Dock10	1.486272865	1.51E-06
Fzd10	1.492601851	0.00334
Gpc4	1.502877236	0.000815
Foxo6	1.510739547	2.16E-06
Prima1	1.514882829	5.33E-05
Nt5dc3	1.516026405	3.84E-05
Arhgef26	1.522257695	5.54E-10
Soat1	1.522759101	0.021481
Hey1	1.526954243	1.52E-05
Hunk	1.528093554	0.010427
LOC108348064	1.552002271	3.18E-07
Itgb4	1.556017551	0.005257
LOC100911779	1.557190573	0.036682
Rreb1	1.558439428	0.00363
Nkrfl	1.562589231	0.003219
Cacna1h	1.566871288	4.91E-05
Sema3e	1.577213413	0.000386
Plekhg1	1.579511451	2.39E-05
Cldn11	1.585371023	0.000598
Gramd1c	1.586596094	0.007093
Slc16a2	1.595177281	0.006812
Gpr176	1.612519326	7.29E-05
Dnah9	1.616564016	8.69E-05
Crlf1	1.621626766	0.000361
Lrp1b	1.624721679	1.55E-10
Nrp1	1.627873035	0.001268
Slc16a14	1.630047013	0.002313
Pxmp4	1.631320524	0.000957
Slc45a3	1.632974881	0.005885
Adamts3	1.634201754	3.92E-18
Syngr2	1.636173247	0.001341
Cd55	1.637501301	0.000141
Fam241a	1.64119591	0.002536
Pxdn	1.642210344	0.000447
Agmo	1.642828644	0.006353
Pgm5	1.644191573	0.007471
Il16	1.646080785	4.15E-06
Kctd4	1.648863631	0.011063
Kcng2	1.654720255	0.011808
Nr4a3	1.658874625	0.000666
Hhip	1.664914233	0.013743
Smo	1.667321076	0.000189
Pkp2	1.669305969	0.021276
Unc5c	1.672917011	0.044281
Kcnip2	1.687388318	0.000181
Tmem88b	1.690775263	7.46E-08
St5	1.692053583	3.15E-05
St6galnac5	1.739910178	5.79E-05
Arl5c	1.741982309	0.001093
LOC100912481	1.74448686	0.001384
Tle1	1.745175961	5.07E-10
Lrrn1	1.753986814	9.58E-08

Nipal4	1.755537763	0.00206
Tmem54	1.756402643	9.52E-07
Wnt2	1.75828048	0.030209
Tnfrsf19	1.758889329	0.016257
Ppm1e	1.766508971	0.00101
Rasgrp1	1.773178532	2.62E-06
Dusp5	1.778107874	0.00144
Pou3f1	1.785964272	3.26E-11
Elovl7	1.79282738	0.011102
Piga	1.793140849	0.000233
Pcdhga12	1.793169456	0.002322
Ttyh2	1.803539823	0.000161
Tgfbr3	1.803979114	0.015962
Dusp4	1.806159342	0.032753
Wipf3	1.832423682	2.08E-06
LOC102555457	1.833456063	0.028708
Gdf10	1.835154718	0.013201
Rerg	1.837442445	7.63E-05
Lyst	1.838227714	7.97E-09
Bhlhe22	1.842286452	0.037389
LOC108348108	1.864221808	0.041228
Gpr161	1.866998177	0.001298
Clca1	1.905692426	0.029817
Arg1	1.908075555	0.03281
Mroh6	1.91112442	0.030148
St18	1.913173266	1.28E-08
Grhl1	1.91521497	0.010701
Cldn1	1.915479695	0.030277
Itga11	1.921700614	0.016216
Sncg	1.924832409	5.68E-05
Kit	1.929751011	0.000352
Tmem125	1.940959493	0.002861
Dgat2	1.945754638	3.28E-08
Dhx35	1.950507284	0.001129
Dock4	1.967982432	1.26E-13
Nr3c2	1.972246146	2.14E-05
Tmem74	1.980933844	0.005993
Fbn1	1.998450363	0.001268
Calml4	2.004127504	2.24E-05
Foxo1	2.004684217	0.000587
Pcsk1	2.074975503	0.04803
Pdyn	2.080229983	9.08E-05
Smpdl3b	2.100145282	2.79E-06
Slc2a9	2.124168648	0.002306
LOC108348049	2.132967363	3.94E-06
Tp53inp1	2.138547085	3.27E-08
NEWGENE_620180	2.161031178	0.034977
Iqgap2	2.166668208	1.42E-11
Sec14l5	2.174958736	2.60E-22
Slc9a2	2.191046245	0.007279
Mmp23	2.224974548	0.001177
Epcam	2.231281041	0.034098
Ccdc85a	2.237029843	0.000306
Gstm6	2.247179629	1.83E-06
Cnot1	2.248133641	0.02344
Icam5	2.250161031	3.96E-07

Ddit4l	2.255267487	0.003829
Lpl	2.261929572	2.42E-06
Hpca	2.278117429	4.76E-10
Mpp7	2.304082395	7.04E-05
Tanc1	2.325914636	3.56E-06
Vav3	2.341455131	7.27E-12
Kcp	2.343514311	1.71E-07
Gjd2	2.384788763	0.005724
Galnt17	2.41381329	0.000136
Scn4a	2.423264151	0.011383
Atp2a1	2.43080032	0.007082
Kifc3	2.431698851	0.027406
Itgbl1	2.462997305	0.000278
RT1-CE13	2.463988773	0.026291
Col6a5	2.472679332	0.013921
Lhx9	2.475191724	0.00167
Piezo2	2.475451982	0.001367
Klk10	2.515717369	0.016935
Kcns2	2.579073625	0.010334
Krt27	2.599029455	0.007265
Plppr1	2.601157719	6.10E-10
Rem2	2.620664302	1.14E-06
Neurog2	2.626679203	0.018592
Dip2a	2.71685633	0.006559
LOC108349745	2.717911453	0.032848
Htr1a	2.861521536	0.03199
Twist1	2.896067171	0.000473
Shisa6	2.897454885	0.00917
Ppl	2.910607302	2.58E-05
Fgfr4	2.94375611	0.001661
Htr4	2.953203572	0.00334
LOC688286	3.015013234	0.025234
LOC102555817	3.020636089	0.046084
Lingo3	3.043133068	4.61E-08
Btbd16	3.075969292	0.001845
Grin2a	3.132890309	0.000511
Fibcd1	3.144059891	2.24E-07
Meox2	3.187945377	2.07E-05
Prox1	3.238904291	1.78E-09
Amfr	3.286116219	0.022702
Lyzl4	3.300834408	2.11E-05
Tdrd12	3.305332608	0.049863
Mas1	3.313017612	0.00184
Cabp7	3.363718918	8.25E-07
LOC102552204	3.385604641	0.008308
Rpl39l	3.399662492	0.003406
Tcam1	3.415700283	0.002104
Tmem30b	3.473774885	0.040918
Slc9a4	3.533220798	0.001341
Itln1	3.546693669	0.045534
RGD1562811	3.564640576	0.017244
Lrrc10b	3.576427547	9.91E-11
Fat4	3.656662386	1.48E-05
Nts	3.71554122	0.01678
Timd4	3.739170061	0.000193
Abcb1b	3.864312926	5.42E-05

Svep1	3.865623496	1.63E-08
RGD1564382	3.888852818	0.019546
Itprid1	3.961940982	0.025001
Nr2c2	4.050879701	0.007074
Ghsr	4.073002008	0.00132
Crebbp	4.107670795	0.011337
LOC102553270	4.171146703	4.47E-06
Clec9a	4.22035788	0.01572
Crygd	4.276822431	0.030695
Epha8	4.348494662	3.83E-24
Chst9	4.385286774	2.54E-05
Prdm4	4.569338302	0.019712
Fzd5	4.711003457	0.036818
Klk7	4.715465402	0.002133
Gria3	4.857238257	0.021937
Vstm1	5.059924698	0.00422
LOC300303	5.140497212	0.018583
Otop3	5.153909927	0.000346
Ephx1	5.485614402	0.014798
Cd3e	5.502043874	1.73E-07
Wnt3a	5.58141585	0.001851
Serpina3n	5.728237679	5.23E-07
Btg1	5.810560726	0.008425
Cxcr1	5.912479062	1.06E-06
Nhlh1	5.917110984	0.000301
LOC102546572	6.321917967	0.018414
Cd320	7.22722404	0.032246
C1ql2	7.261671561	2.98E-19
Smpd2	7.769853824	0.003332
Nelfcd	7.808807978	0.028534
Smg5	8.047522652	0.013686
Klk8	8.376454255	3.30E-05
Vta1	10.42018014	0.025474
Vps39	10.5472062	0.007435
Trim37	25.90192809	7.59E-11
