# Supplementary material for: Transcriptomic Profiling of Electroacupuncture Regulating the Molecular Network in Hippocampus of Rats with Cerebral Ischemia-Reperfusion Injury
Source: Evid Based Complement Alternat Med. 2022 Sep 2;2022:6053106. doi: 10.1155/2022/6053106 (PMC9463016; doi:10.1155/2022/6053106)
Supplement: Supplementary Materials — Table S1: differentially expressed genes of Model/Sham group; Table S2: preliminary enrichment results of Model/Sham group; Table S3: differentially expressed genes of EA/Model group; Table S4: upregulated gene analysis; Table S5: downregulated gene analysis; Table S6: all gene analysis. [file 6053106.f1.zip › Table S4 (1).pdf]

Table S4 Up-regulated Gene Analysis

Category	Term
GO Biological Processes	GO:0061564
GO Biological Processes	GO:0032989
GO Biological Processes	GO:0034330
GO Biological Processes	GO:0031175
GO Biological Processes	GO:0048812
GO Biological Processes	GO:0120039
GO Biological Processes	GO:0048858
GO Biological Processes	GO:0032990
GO Biological Processes	GO:0007409
GO Biological Processes	GO:0007155
GO Biological Processes	GO:0007411
GO Biological Processes	GO:0097485
GO Biological Processes	GO:0006935
GO Biological Processes	GO:0042330
GO Biological Processes	GO:0048667
GO Biological Processes	GO:0050808
GO Biological Processes	GO:0000902
GO Biological Processes	GO:0000904
GO Biological Processes	GO:0007215
GO Biological Processes	GO:0050890
GO Biological Processes	GO:0007423
GO Biological Processes	GO:0033674
GO Biological Processes	GO:0007611
GO Biological Processes	GO:0009611
GO Biological Processes	GO:0043583
GO Biological Processes	GO:0048729
GO Biological Processes	GO:0072574
GO Biological Processes	GO:0002315
GO Biological Processes	GO:0072575
GO Biological Processes	GO:1901888
GO Biological Processes	GO:0001568
GO Biological Processes	GO:0021782
GO Biological Processes	GO:0010001
GO Biological Processes	GO:0007272
GO Biological Processes	GO:0008366
GO Biological Processes	GO:0001944
GO Biological Processes	GO:0007169
GO Biological Processes	GO:0048839
GO Biological Processes	GO:0072576
GO Biological Processes	GO:0051963
GO Biological Processes	GO:0043524
GO Biological Processes	GO:0050919
GO Biological Processes	GO:0051347
GO Biological Processes	GO:0050679
GO Biological Processes	GO:0032763
GO Biological Processes	GO:0035239
GO Biological Processes	GO:0048678
GO Biological Processes	GO:0007610
GO Biological Processes	GO:0060078
GO Biological Processes	GO:0048169
GO Biological Processes	GO:0030308
GO Biological Processes	GO:0097061
GO Biological Processes	GO:0045216
GO Biological Processes	GO:0048514

GO Biological Processes	GO:0050771
GO Biological Processes	GO:0042552
GO Biological Processes	GO:0042063
GO Biological Processes	GO:0007167
GO Biological Processes	GO:0033564
GO Biological Processes	GO:0007517
GO Biological Processes	GO:1901215
GO Biological Processes	GO:0098609
GO Biological Processes	GO:0021510
GO Biological Processes	GO:0006929
GO Biological Processes	GO:0003007
GO Biological Processes	GO:0001508
GO Biological Processes	GO:0051272
KEGG Pathway	rno04360
KEGG Pathway	rno05217
KEGG Pathway	rno04724
KEGG Pathway	rno05033
KEGG Pathway	rno04330
KEGG Pathway	rno04720
KEGG Pathway	rno04010
KEGG Pathway	rno04340
KEGG Pathway	rno04713
KEGG Pathway	rno04310
Reactome Gene Sets	R-RNO-112316
Reactome Gene Sets	R-RNO-6794362
Reactome Gene Sets	R-RNO-112315
Reactome Gene Sets	R-RNO-112314
Reactome Gene Sets	R-RNO-438066
Reactome Gene Sets	R-RNO-425407
Reactome Gene Sets	R-RNO-9013408
Reactome Gene Sets	R-RNO-388844
Reactome Gene Sets	R-RNO-3928665
Reactome Gene Sets	R-RNO-442755
Reactome Gene Sets	R-RNO-9006934
Reactome Gene Sets	R-RNO-8849932
Reactome Gene Sets	R-RNO-112409
Reactome Gene Sets	R-RNO-210500

Description	Pvalue	Enrichmei
axon development	1E-10	3.7
cellular component morphogenesis	1E-10	3.1
cell junction organization	2.51E-10	3.3
neuron projection development	5.01E-10	2.8
neuron projection morphogenesis	1E-09	3.2
plasma membrane bounded cell projection morphogenesis	1.58E-09	3.2
cell projection morphogenesis	2E-09	3.2
cell part morphogenesis	7.94E-09	3
axonogenesis	7.94E-09	3.5
cell adhesion	2E-08	2.5
axon guidance	5.01E-08	4.1
neuron projection guidance	5.01E-08	4.1
chemotaxis	6.31E-08	3
taxis	7.94E-08	3
cell morphogenesis involved in neuron differentiation	7.94E-08	3
synapse organization	2E-07	3.4
cell morphogenesis	2E-07	2.4
cell morphogenesis involved in differentiation	6.31E-07	2.6
glutamate receptor signaling pathway	7.94E-07	8.6
cognition	2.51E-06	2.9
sensory organ development	0.00001	2.3
positive regulation of kinase activity	1.58E-05	2.5
learning or memory	1.58E-05	2.9
response to wounding	1.58E-05	2.5
ear development	2E-05	3.2
tissue morphogenesis	2E-05	2.3
hepatocyte proliferation	2.51E-05	21
marginal zone B cell differentiation	2.51E-05	21
epithelial cell proliferation involved in liver morphogenesis	2.51E-05	21
regulation of cell junction assembly	3.16E-05	3.3
blood vessel development	3.16E-05	2.4
glial cell development	3.16E-05	4.1
glial cell differentiation	3.98E-05	3.3
ensheathment of neurons	3.98E-05	4.1
axon ensheathment	3.98E-05	4.1
vasculature development	5.01E-05	2.3
transmembrane receptor protein tyrosine kinase signaling pathway	5.01E-05	2.7
inner ear development	6.31E-05	3.1
liver morphogenesis	6.31E-05	17
regulation of synapse assembly	6.31E-05	4.2
negative regulation of neuron apoptotic process	6.31E-05	3.4
negative chemotaxis	7.94E-05	6.7
positive regulation of transferase activity	7.94E-05	2.2
positive regulation of epithelial cell proliferation	7.94E-05	3.2
regulation of mast cell cytokine production	0.0001	28
tube morphogenesis	0.0001	2.1
response to axon injury	0.0001	4.8
behavior	0.0001	2
regulation of postsynaptic membrane potential	0.0001	4.3
regulation of long-term neuronal synaptic plasticity	0.000126	7.6
negative regulation of cell growth	0.000126	3.2
dendritic spine organization	0.000126	7.4
cell-cell junction organization	0.000158	3.4
blood vessel morphogenesis	0.000158	2.5

negative regulation of axonogenesis	0.000158	5.1
myelination	0.000158	3.8
gliogenesis	0.000158	2.8
enzyme linked receptor protein signaling pathway	0.0002	2.1
anterior/posterior axon guidance	0.0002	23
muscle organ development	0.0002	2.7
negative regulation of neuron death	0.0002	2.8
cell-cell adhesion	0.000251	2.3
spinal cord development	0.000251	3.6
substrate-dependent cell migration	0.000251	8.7
heart morphogenesis	0.000251	2.8
action potential	0.000251	4.3
positive regulation of cellular component movement	0.000251	2.1
Axon guidance	0.000126	3.4
Basal cell carcinoma	0.000316	5.3
Glutamatergic synapse	0.000794	3.7
Nicotine addiction	0.001585	5.9
Notch signaling pathway	0.001995	4.5
Long-term potentiation	0.003162	4.2
MAPK signaling pathway	0.003981	2.2
Hedgehog signaling pathway	0.005012	4.5
Circadian entrainment	0.005012	3.4
Wnt signaling pathway	0.01	2.6
Neuronal System	0.000001	3.4
Protein-protein interactions at synapses	3.16E-06	7.4
Transmission across Chemical Synapses	0.000398	3.2
Neurotransmitter receptors and postsynaptic signal transmission	0.000794	3.7
Unblocking of NMDA receptors, glutamate binding and activation	0.001	8.5
SLC-mediated transmembrane transport	0.001259	2.8
RHOG GTPase cycle	0.001585	4.8
Receptor-type tyrosine-protein phosphatases	0.003981	9.4
EPH-ephrin mediated repulsion of cells	0.003981	6.1
Activation of NMDA receptors and postsynaptic events	0.003981	6.1
Signaling by Receptor Tyrosine Kinases	0.00631	2.1
Synaptic adhesion-like molecules	0.007943	7.4
RAF-independent MAPK1/3 activation	0.007943	7
Glutamate Neurotransmitter Release Cycle	0.01	6.7

Counts	Genes
33	Pmp22|Plp1|Apod|Smo|Cnp|Dcc|Ddr1|Epha6|Mag|Erbb3|Nr4a3|Epha8|Slit3|Nefl|Efna2|Epha7|
42	Pmp22|Smo|Cnp|Dcc|Ddr1|Itgb4|Epha6|Mag|Erbb3|Ugt8|Nr4a3|Cttn|Epha8|Slit3|Nefl|Chn2|Ef
37	Grin2a|Grm5|Htr1a|Pdgfb|Pmp22|Gabrb3|Il1rap|Slc1a1|Itgb4|Ugt8|Ptk2b|Cttn|Sncg|Cldn1|Cea
45	Pmp22|Plp1|Apod|Smo|Cnp|Dcc|Ddr1|Epha6|Mag|Erbb3|Ugt8|Ptk2b|Nr4a3|Cttn|Epha8|Slit3|N
35	Pmp22|Smo|Cnp|Dcc|Ddr1|Epha6|Erbb3|Ugt8|Nr4a3|Cttn|Epha8|Slit3|Nefl|Efna2|Epha7|Sema
35	Pmp22|Smo|Cnp|Dcc|Ddr1|Epha6|Erbb3|Ugt8|Nr4a3|Cttn|Epha8|Slit3|Nefl|Efna2|Epha7|Sema
35	Pmp22|Smo|Cnp|Dcc|Ddr1|Epha6|Erbb3|Ugt8|Nr4a3|Cttn|Epha8|Slit3|Nefl|Efna2|Epha7|Sema
35	Pmp22|Smo|Cnp|Dcc|Ddr1|Epha6|Erbb3|Ugt8|Nr4a3|Cttn|Epha8|Slit3|Nefl|Efna2|Epha7|Sema
28	Pmp22|Smo|Cnp|Dcc|Epha6|Erbb3|Nr4a3|Epha8|Slit3|Efna2|Epha7|Sema3d|Nrp1|Lgi1|Lhx9|P
43	Mog|Pmp22|Dcc|Il1rap|Ddr1|Itgb4|Mag|Ptk2b|Acan|Ninj2|Tcam1|Cttn|Epha8|Kit|Cldn1|Mcam
21	Smo|Dcc|Epha6|Nr4a3|Epha8|Slit3|Efna2|Epha7|Sema3d|Nrp1|Lgi1|Lhx9|Neurog2|Sema3e|W
21	Smo|Dcc|Epha6|Nr4a3|Epha8|Slit3|Efna2|Epha7|Sema3d|Nrp1|Lgi1|Lhx9|Neurog2|Sema3e|W
31	Pdgfb|Scg2|Smo|Dcc|Epha6|Cxcr1|Nr4a3|Epha8|Kit|Slit3|Efna2|Lyst|Il16|Epha7|Sema3d|Nrp1
31	Pdgfb|Scg2|Smo|Dcc|Epha6|Cxcr1|Nr4a3|Epha8|Kit|Slit3|Efna2|Lyst|Il16|Epha7|Sema3d|Nrp1
30	Pmp22|Smo|Cnp|Dcc|Epha6|Erbb3|Nr4a3|Epha8|Slit3|Efna2|Epha7|Sema3d|Nrp1|Lgi1|Lhx9|P
24	Grin2a|Grm5|Htr1a|Pdgfb|Pmp22|Gabrb3|Il1rap|Slc1a1|Cttn|Sncg|Nefl|Ppfia4|Nrp1|Sema3e|A
40	Pmp22|Smo|Cnp|Dcc|Ddr1|Epha6|Erbb3|Ugt8|Nr4a3|Cttn|Epha8|Msx1|Slit3|Nefl|Efna2|Epha7
33	Pmp22|Smo|Cnp|Dcc|Epha6|Erbb3|Nr4a3|Epha8|Slit3|Efna2|Epha7|Sema3d|Nrp1|Lgi1|Lhx9|S
9	Grin2a|Grm1|Grm5|Plp1|Slc1a1|Homer3|Gria3|Gria1|Grik2
24	Cebpb|Egr1|Grin2a|Grm5|Gabrb3|Htr4|Slc1a1|Gabra5|Kcnab1|Gria1|Crebbp|Kit|Arl6ip5|Ghsr|
33	Crygd|Pdgfb|Gabrb3|Slc1a1|Ddr1|Cebpd|Hpca|Notch2|Erbb3|Gabra5|Nr4a3|Kit|Msx1|Fbn1|Dl
26	Egr1|Grm5|Pdgfb|Tgfa|Fgfr4|Mas1|Slc1a1|Ddr1|Epha6|Rasgrp1|Erbb3|Ptk2b|Epha8|Kit|Ceaca
21	Cebpb|Egr1|Grin2a|Grm5|Gabrb3|Slc1a1|Gabra5|Kcnab1|Gria1|Crebbp|Kit|Arl6ip5|Ghsr|Nts|A
27	Egr1|Grin2a|Pdgfb|Tgfa|Pcsk1|Apod|Smo|Slc1a1|Ddr1|Itgb4|Arg1|Mag|Notch2|Erbb3|Pllp|Cld
18	Gabrb3|Ddr1|Cebpd|Hpca|Gabra5|Nr4a3|Msx1|Dll1|Efna2|Hey1|Plppr4|Wnt3a|Gli2|Prox1|Ror
31	Pdgfb|Smo|Ddr1|Itgb4|Notch2|Tgfbr3|Nr4a3|Ceacam1|Msx1|Dll1|Twist1|Wnt2|Hey1|Epha7|N
4	Cebpb|Tgfa|Notch2|Prox1
4	Notch2|Ptk2b|Dll1|Dock10
4	Cebpb|Tgfa|Notch2|Prox1
16	Apod|Il1rap|Cldn1|Ghsr|Epha7|Clstn2|Nrp1|Pkp2|Adgrb3|Wnt3a|Slitrk2|Ephb2|Gpc4|Fzd5|Flrt
26	Egr1|Pdgfb|Scg2|Tgfa|Chm|Smo|Slc1a1|Meox2|Notch2|Tgfbr3|Ptk2b|Mcam|Ceacam1|Dll1|Fo
12	Pdgfb|Pmp22|Plp1|Mal|Smo|Itgb4|Mag|Dll1|Pou3f1|Ror2|Fa2h|Nkx6-2
16	Egr1|Pdgfb|Pmp22|Plp1|Mal|Smo|Cnp|Itgb4|Mag|Erbb3|Dll1|Pou3f1|Ror2|Fa2h|Nkx6-2|Gpr17
12	Pmp22|Plp1|Mal|Itgb4|Mag|Ugt8|Pllp|Cldn11|Serinc5|Pou3f1|Fa2h|Nkx6-2
12	Pmp22|Plp1|Mal|Itgb4|Mag|Ugt8|Pllp|Cldn11|Serinc5|Pou3f1|Fa2h|Nkx6-2
27	Egr1|Pdgfb|Scg2|Tgfa|Chm|Smo|Slc1a1|Meox2|Notch2|Tgfbr3|Ptk2b|Mcam|Ceacam1|Dll1|Fo
21	Pdgfb|Tgfa|Fgfr4|Ddr1|Epha6|Erbb3|Ptk2b|Nr4a3|Epha8|Kit|Efna2|Foxo1|Epha7|Nrp1|Slc30a1
16	Gabrb3|Cebpd|Hpca|Gabra5|Nr4a3|Dll1|Efna2|Hey1|Plppr4|Wnt3a|Gli2|Prox1|Ror2|Fat4|Ephb
4	Cebpb|Tgfa|Notch2|Prox1
11	Il1rap|Ghsr|Epha7|Clstn2|Adgrb3|Wnt3a|Slitrk2|Ephb2|Gpc4|Flrt3|Lrrn1
14	Cebpb|Gabrb3|Smo|Slc1a1|Mag|Erbb3|Gabra5|Ptk2b|Grik2|Nr4a3|Nefl|Nrp1|Slc30a10|Crlf1
7	Slit3|Epha7|Sema3d|Sema3e|Sema5a|Unc5c|Flrt3
27	Egr1|Grm5|Pdgfb|Tgfa|Fgfr4|Mas1|Slc1a1|Ddr1|Epha6|Rasgrp1|Erbb3|Ptk2b|Epha8|Kit|Ceaca
15	Pdgfb|Scg2|Tgfa|Smo|Arg1|Notch2|Nr4a3|Cldn1|Ghsr|Twist1|Wnt2|Wnt3a|Prox1|Rreb1|Sema
3	Nr4a3|Kit|Bcl6
31	Pdgfb|Scg2|Tgfa|Smo|Slc1a1|Ddr1|Meox2|Notch2|Tgfbr3|Ptk2b|Nr4a3|Mcam|Dll1|Twist1|Hey
9	Pdgfb|Pcsk1|Apod|Slc1a1|Arg1|Mag|Nefl|Nts|Flrt3
32	Cebpb|Egr1|Grin2a|Grm1|Grm5|Htr1a|Pmp22|Gabrb3|Cnp|Slc1a1|Gabra5|Kcnab1|Gria1|Crebb
10	Grin2a|Grm1|Grm5|Gabrb3|Nr3c2|Gria3|Gabra5|Gria1|Grik2|Chrna2
6	Egr1|Grin2a|Grm5|Grik2|Kit|Ephb2
14	Mag|Msx1|Slit3|Prdm4|Epha7|Sema3d|Nrp1|Sema3e|Wnt3a|Bcl6|Tomm70|Sema5a|Rerg|Hspa
6	Grin2a|Cttn|Dock10|Tanc1|Ephb2|Dip2a
13	Pmp22|Ugt8|Cldn1|Ceacam1|Cldn11|Pkp2|Dsp|Mpp7|Kifc3|Ephb2|Grhl1|Tjp3|Svep1
21	Pdgfb|Scg2|Tgfa|Smo|Slc1a1|Meox2|Notch2|Tgfbr3|Ptk2b|Mcam|Dll1|Hey1|Nrp1|Sema3e|Plx

8	Mag|Epha7|Sema3d|Nrp1|Sema3e|Wnt3a|Sema5a|Ephb2
11	Pmp22|Plp1|Mal|Itgb4|Mag|Ugt8|Pllp|Serinc5|Pou3f1|Fa2h|Nkx6-2
17	Egr1|Pdgfb|Pmp22|Plp1|Mal|Smo|Cnp|Itgb4|Mag|Erbb3|Ptk2b|Dll1|Pou3f1|Ror2|Fa2h|Nkx6-2|
27	Egr1|Pdgfb|Tf|Tgfa|Fgfr4|Ddr1|Epha6|Erbb3|Tgfbr3|Ptk2b|Nr4a3|Epha8|Kit|Gdf10|Msx1|Efna
3	Dcc|Lhx9|Unc5c
17	Egr1|Pmp22|Smo|Meox2|Erbb3|Tgfbr3|Kcnab1|Msx1|Dll1|Wnt2|Hey1|Pkp2|Wnt3a|Prox1|My
16	Cebpb|Gabrb3|Smo|Slc1a1|Mag|Erbb3|Gabra5|Ptk2b|Grik2|Nr4a3|Sncg|Nefl|Nrp1|Slc30a10|C
23	Dcc|Il1rap|Mag|Tcam1|Kit|Cldn1|Ceacam1|Cldn11|Clstn2|Epcam|Pkp2|Wnt3a|Dsp|Pcdh1|Kif
11	Grin2a|Smo|Dcc|Gria1|Acan|Slit3|Nefl|Dll1|Wnt3a|Plxdc1|Gli2
5	Pdgfb|Cttn|Epha8|Nrp1|Itga11
16	Smo|Notch2|Tgfbr3|Msx1|Slit3|Dll1|Twist1|Wnt2|Hey1|Nrp1|Pkp2|Wnt3a|Prox1|Dsp|Fat4|Zfp
9	Grin2a|Pmp22|Scn4a|Gjd2|Gria1|Grik2|Cacna1h|Scn3b|Pkp2
28	Egr1|Grm1|Pdgfb|Tf|Plp1|Smo|Ptp4a1|Ptk2b|Nr4a3|Kit|Cldn1|Mcam|Twist1|Cacna1h|Atp2a1|
13	Smo|Dcc|Epha6|Epha8|Slit3|Efna2|Epha7|Sema3d|Nrp1|Sema3e|Sema5a|Ephb2|Unc5c
7	Smo|Wnt2|Hhip|Wnt3a|Gli2|Fzd5|Fzd10
9	Grin2a|Grm1|Grm5|Slc1a1|Homer3|Gria3|Gria1|Grik2|Gnb4
5	Grin2a|Gabrb3|Gria3|Gabra5|Gria1
6	Notch2|Crebbp|Dll1|Hey1|Aph1b|Tle1
6	Grin2a|Grm1|Grm5|Gria1|Crebbp|Calml4
14	Pdgfb|Tgfa|Fgfr4|Il1rap|Rasgrp1|Erbb3|Dusp4|Kit|Efna2|Cacna1h|Dusp6|Cacng8|Dusp5|Hspa1
5	Smo|Gpr161|Hhip|Gli2|Scube2
7	Grin2a|Gria3|Gria1|Cacna1h|Ryr3|Gnb4|Calml4
9	Crebbp|Wnt2|Wnt3a|Ror2|Gpc4|Fzd5|Tle1|Fzd10|Frat1
21	Grin2a|Grm1|Grm5|Gabrb3|Il1rap|Slc1a1|Homer3|Gria3|Gabra5|Kcnab1|Gjd2|Gria1|Grik2|Kc
9	Grin2a|Grm1|Grm5|Il1rap|Homer3|Gria3|Gria1|Ppfia4|Slitrk2
12	Grin2a|Gabrb3|Slc1a1|Gria3|Gabra5|Gria1|Grik2|Arl6ip5|Nefl|Ppfia4|Chrna2|Gnb4
9	Grin2a|Gabrb3|Gria3|Gabra5|Gria1|Grik2|Nefl|Chrna2|Gnb4
4	Grin2a|Gria3|Gria1|Nefl
12	Slc9a2|Slc9a4|Apod|Slc1a1|Slc6a20|Slc16a2|Slc30a10|Slc35d1|Slc45a3|Ahcyl2|Slc44a5|Slc2a
6	Mcam|Vav3|Mpp7|Arhgef26|Ankle2|Elmo2
3	Il1rap|Ppfia4|Slitrk2
4	Efna2|Epha7|Vav3|Aph1b
4	Grin2a|Gria3|Gria1|Nefl
15	Pdgfb|Tgfa|Fgfr4|Erbb3|Ptk2b|Dusp4|Kit|Dusp6|Nrp1|Vav3|Aph1b|Elmo2|Flrt3|Thbs3|Ncbp2
3	Grin2a|Gria3|Gria1
3	Dusp4|Dusp6|Dusp5
3	Slc1a1|Arl6ip5|Ppfia4

Sema3d|Nrp1|Lgi1|Lhx9|Plppr4|Neurog2|Sema3e|Wnt3a|Gli2|Slitrk2|Sema5a|Ephb2|Bcl11b|Unc5c|Bhlhe2
na2|Epha7|Sema3d|Nrp1|Lgi1|Lhx9|Plppr4|Neurog2|Sema3e|Dock10|Wnt3a|Gli2|Prox1|Myom2|Klk8|Slitrk
cam1|Nefl|Cldn11|Ppfia4|Nrp1|Pkp2|Sema3e|Adgrb3|Dock10|Dsp|Mpp7|Kifc3|Klk8|Slitrk2|Tanc1|Ephb2|C
efl|Efna2|Gpr37|Hey1|Epha7|Sema3d|Nrp1|Lgi1|Arid1b|Lhx9|Plppr4|Neurog2|Sema3e|Dock10|Wnt3a|Gli2
3d|Nrp1|Lgi1|Lhx9|Plppr4|Neurog2|Sema3e|Dock10|Wnt3a|Gli2|Klk8|Slitrk2|Sema5a|Ephb2|Bcl11b|Unc5
3d|Nrp1|Lgi1|Lhx9|Plppr4|Neurog2|Sema3e|Dock10|Wnt3a|Gli2|Klk8|Slitrk2|Sema5a|Ephb2|Bcl11b|Unc5
3d|Nrp1|Lgi1|Lhx9|Plppr4|Neurog2|Sema3e|Dock10|Wnt3a|Gli2|Klk8|Slitrk2|Sema5a|Ephb2|Bcl11b|Unc5
3d|Nrp1|Lgi1|Lhx9|Plppr4|Neurog2|Sema3e|Dock10|Wnt3a|Gli2|Klk8|Slitrk2|Sema5a|Ephb2|Bcl11b|Unc5

|Ceacam1|Mybph|Fbn1|Cldn11|Clstn2|Epcam|Nrp1|Pkp2|Tecta|Wnt3a|Dsp|Pcdh1|Kifc3|Slitrk2|Fat4|Fibcd

|Lgi1|Lhx9|Vav3|Neurog2|Sema3e|Wnt3a|Gli2|Sema5a|Ephb2|Bcl11b|Gab1|Unc5c|Elmo2|Flrt3|Dock4
|Lgi1|Lhx9|Vav3|Neurog2|Sema3e|Wnt3a|Gli2|Sema5a|Ephb2|Bcl11b|Gab1|Unc5c|Elmo2|Flrt3|Dock4
lppr4|Neurog2|Sema3e|Dock10|Wnt3a|Gli2|Slitrk2|Sema5a|Ephb2|Bcl11b|Unc5c|Bhlhe22|Flrt3|Dip2a|Auts

|Sema3d|Nrp1|Lgi1|Lhx9|Sipa1l3|Plppr4|Neurog2|Sema3e|Dock10|Wnt3a|Bcl6|Gli2|Prox1|Klk8|Slitrk2|Se
ipa1l3|Plppr4|Neurog2|Sema3e|Dock10|Wnt3a|Gli2|Prox1|Slitrk2|Sema5a|Arhgef26|Ephb2|Bcl11b|Unc5c|

l1|Efna2|Twist1|Hey1|Nrp1|Sipa1l3|Plppr4|Wnt3a|Fscn2|Gli2|Prox1|Myom2|Ror2|Fat4|Ephb2|Bcl11b|Fzd5

rp1|Pkp2|Hhip|Sema3e|Wnt3a|Gli2|Prox1|Dsp|Sema5a|Fat4|Zfpm2|Fzd5|Flrt3|Krt27|Greb1l|Cecr2|Astn2

1|Epha7|Nrp1|Hhip|Sema3e|Wnt3a|Plxdc1|Gli2|Prox1|Sema5a|Fat4|Ephb2|Zfpm2|Fzd5|Gab1|Greb1l|Cecr2

p|Grik2|Nr4a3|Kit|Sncg|Arl6ip5|Ghsr|Gpr176|Gpr37|Nts|Aph1b|Adgrb3|Klk8|Tanc1|Foxo6|Ephb2|Amfr|D

2|Klk8|Slitrk2|Sema5a|Fat4|Ephb2|Bcl11b|Cd3e|Unc5c|Bhlhe22|Flrt3|Cecr2|Dip2a|Auts2
