# Supplementary material for: Transcriptomic Profiling of Electroacupuncture Regulating the Molecular Network in Hippocampus of Rats with Cerebral Ischemia-Reperfusion Injury
Source: Evid Based Complement Alternat Med. 2022 Sep 2;2022:6053106. doi: 10.1155/2022/6053106 (PMC9463016; doi:10.1155/2022/6053106)
Supplement: Supplementary Materials — Table S1: differentially expressed genes of Model/Sham group; Table S2: preliminary enrichment results of Model/Sham group; Table S3: differentially expressed genes of EA/Model group; Table S4: upregulated gene analysis; Table S5: downregulated gene analysis; Table S6: all gene analysis. [file 6053106.f1.zip › Table S5.pdf]

Table S5 Down-regulated Gene Analysis

**Category**	**Term**
GO Biological Processes	GO:0044057
GO Biological Processes	GO:0045744
GO Biological Processes	GO:0001817
GO Biological Processes	GO:0010649
GO Biological Processes	GO:0007200
GO Biological Processes	GO:0001819
GO Biological Processes	GO:0099536
GO Biological Processes	GO:1903522
GO Biological Processes	GO:0006936
GO Biological Processes	GO:0099537
GO Biological Processes	GO:0043271
GO Biological Processes	GO:0006820
GO Biological Processes	GO:0098815
GO Biological Processes	GO:0042391
GO Biological Processes	GO:0007268
GO Biological Processes	GO:0098916
GO Biological Processes	GO:0051963
GO Biological Processes	GO:1903524
GO Biological Processes	GO:0002764
GO Biological Processes	GO:0043269
GO Biological Processes	GO:0050673
GO Biological Processes	GO:0008045
GO Biological Processes	GO:0031644
GO Biological Processes	GO:0071674
GO Biological Processes	GO:2000463
GO Biological Processes	GO:0022401
GO Biological Processes	GO:0002029
GO Biological Processes	GO:1901019
GO Biological Processes	GO:0050853
GO Biological Processes	GO:0048663
KEGG Pathway	rno04750
KEGG Pathway	rno04080
KEGG Pathway	rno04621
KEGG Pathway	rno00140
KEGG Pathway	rno04917
KEGG Pathway	rno04670
KEGG Pathway	rno04974
Reactome Gene Sets	R-RNO-373080
Reactome Gene Sets	R-RNO-500792
Reactome Gene Sets	R-RNO-194068
Reactome Gene Sets	R-RNO-159418
Reactome Gene Sets	R-RNO-168643
Reactome Gene Sets	R-RNO-372790
Reactome Gene Sets	R-RNO-418597
Reactome Gene Sets	R-RNO-416476
Reactome Gene Sets	R-RNO-193807
Reactome Gene Sets	R-RNO-983712
Reactome Gene Sets	R-RNO-381771
Reactome Gene Sets	R-RNO-525793
Reactome Gene Sets	R-RNO-198933
Reactome Gene Sets	R-RNO-400042
Reactome Gene Sets	R-RNO-400508
Reactome Gene Sets	R-RNO-2022090
Reactome Gene Sets	R-RNO-382551

Reactome Gene Sets	R-RNO-1474290
Reactome Gene Sets	R-RNO-622312
Reactome Gene Sets	R-RNO-8957322
Reactome Gene Sets	R-RNO-2672351
Reactome Gene Sets	R-RNO-193368
Reactome Gene Sets	R-RNO-5676594
Reactome Gene Sets	R-RNO-420092
Reactome Gene Sets	R-RNO-418594

Description

regulation of system process
negative regulation of G protein-coupled receptor signaling pathway
regulation of cytokine production
regulation of cell communication by electrical coupling
phospholipase C-activating G protein-coupled receptor signaling pathway
positive regulation of cytokine production
synaptic signaling
regulation of blood circulation
muscle contraction
trans-synaptic signaling
negative regulation of ion transport
anion transport
modulation of excitatory postsynaptic potential
regulation of membrane potential
chemical synaptic transmission
anterograde trans-synaptic signaling
regulation of synapse assembly
positive regulation of blood circulation
immune response-regulating signaling pathway
regulation of ion transport
epithelial cell proliferation
motor neuron axon guidance
regulation of nervous system process
mononuclear cell migration
positive regulation of excitatory postsynaptic potential
negative adaptation of signaling pathway
desensitization of G protein-coupled receptor signaling pathway
regulation of calcium ion transmembrane transporter activity
B cell receptor signaling pathway
neuron fate commitment
Inflammatory mediator regulation of TRP channels
Neuroactive ligand-receptor interaction
NOD-like receptor signaling pathway
Steroid hormone biosynthesis
Prolactin signaling pathway
Leukocyte transendothelial migration
Protein digestion and absorption
Class B/2 (Secretin family receptors)
GPCR ligand binding
Bile acid and bile salt metabolism
Recycling of bile acids and salts
Nucleotide-binding domain, leucine rich repeat containing receptor (NLR) signaling pathways
Signaling by GPCR
G alpha (z) signalling events
G alpha (q) signalling events
Synthesis of bile acids and bile salts via 27-hydroxycholesterol
Ion channel transport
Synthesis, secretion, and inactivation of Glucagon-like Peptide-1 (GLP-1)
Myogenesis
Immunoregulatory interactions between a Lymphoid and a non-Lymphoid cell
Adrenaline,noradrenaline inhibits insulin secretion
Incretin synthesis, secretion, and inactivation
Assembly of collagen fibrils and other multimeric structures
Transport of small molecules

Collagen formation
Inflammasomes
Metabolism of steroids
Stimuli-sensing channels
Synthesis of bile acids and bile salts via 7alpha-hydroxycholesterol
TNF receptor superfamily (TNFSF) members mediating non-canonical NF-kB pathway
Glucagon-type ligand receptors
G alpha (i) signalling events

Pvalue	Enrichment	Counts	Genes
5.01187E-05	2	37	Adra1b|Calca|Camk2d|Igf1|Nppa|Thrb|Adra2a|Th|Cck|Hrh
0.000199526	5	8	Calca|Gipr|Rgs4|Htr2b|Atp2b4|Crhr2|Arr3|Rph3al
0.000199526	1.8	39	Calca|Igf1|Klrk1|Adra2a|Plcg2|Mapk13|Lgals7|Htr2b|P2ry
0.000251189	12	4	Camk2d|Trdn|Cabp1|Hrc
0.000316228	3.8	10	Adra1b|Calca|Adra2a|Npr3|Htr2b|P2ry2|Crhr1|Nmur1|Fpr1
0.000398107	2	28	Calca|Klrk1|Adra2a|Plcg2|Mapk13|Htr2b|P2ry2|Runx1|Prg
0.000501187	2.1	25	Th|Chrna5|Hrh2|Chrna4|Glra1|Htr2b|Cacna1g|Pclo|Doc2a|
0.000794328	2.4	18	Adra1b|Calca|Camk2d|Nppa|Thrb|Adra2a|Th|Hrh2|Shox2|
0.000794328	2.6	15	Camk2d|Scn5a|Glra1|Htr2b|Atp2b4|Cacna1g|Nmur1|Chrna
0.000794328	2.1	23	Th|Chrna5|Hrh2|Chrna4|Glra1|Htr2b|Cacna1g|Pclo|Doc2a|
0.000794328	2.7	14	Calca|Camk2d|Plcb4|Adra2a|Hrh2|Rgs4|Crhr1|Trdn|Il1rn|C
0.000794328	2	25	Mip|Glra1|Slc7a3|Slc10a2|Slco1b2|Nr1h4|Gabrq|Gabre|Nm
0.001	4.4	7	Chrna4|Rgs4|Pclo|Stx1a|Grip2|Cux2|Ssh1
0.001258925	1.9	26	Camk2d|Nppa|Chrna5|Cck|Chrna4|Scn5a|Glra1|Rgs4|Kcnk
0.001995262	2	21	Th|Chrna5|Hrh2|Chrna4|Glra1|Htr2b|Cacna1g|Pclo|Doc2a|
0.001995262	2	21	Th|Chrna5|Hrh2|Chrna4|Glra1|Htr2b|Cacna1g|Pclo|Doc2a|
0.002511886	2.9	10	Tpbg|Efna5|Cux2|Cbln2|Dkk1|Lrrtm3|Il1rapl2|Farp1|Mef2
0.002511886	4.4	6	Adra1b|Nppa|Scn5a|Rgs4|Crhr2|Hrc
0.002511886	2.2	16	Klrk1|Cd247|Plcg2|Cd14|Nr1h4|Sh2b2|Cd40|Fpr1|Ermap|
0.003162278	1.6	35	Calca|Camk2d|Igf1|Nppa|Plcb4|Adra2a|Cck|Hrh2|Chrna4|S
0.003162278	3	9	Calca|Nr1h4|Olr59|Fap|Loxl2|Lgr5|Bmper|Col8a1|Mef2c
0.003162278	4.9	5	Nog|Sema3a|Lhx3|Lamc2|Mycbp2
0.003162278	2.4	13	Calca|Igf1|Cck|Chrna4|Glra1|Rgs4|Scn11a|Pclo|Nrg1|Stx1a
0.003162278	3.2	8	Calca|Itgb7|Plg|Hsd3b7|Gpr15|Ccl24|Ccl9|Cxcl13
0.003981072	4.7	5	Rgs4|Stx1a|Grip2|Cux2|Ssh1
0.003981072	5.9	4	Calca|Gipr|Htr2b|Arr3
0.003981072	5.9	4	Calca|Gipr|Htr2b|Arr3
0.003981072	3.1	8	Nppa|Adra2a|Plcg2|Crhr1|Trdn|Cabp1|Hrc|Stac2
0.005011872	4.6	5	Plcg2|Sh2b2|Mnda|Blk|Mef2c
0.005011872	3.4	7	Nkx2-1|Pou3f2|Runx1|Nrg1|Lhx3|Satb2|Epop
3.98107E-06	4.3	14	Camk2d|Igf1|Plcb4|Plcg2|Trpv2|Mapk13|Htr2b|P2ry2|Map
6.30957E-05	2.4	25	Adra1b|Calca|Sst|Thrb|Gipr|Adra2a|Chrna5|Gnrh1|Cck|Hrh
0.000158489	3	15	Plcb4|Trpv2|Mapk13|Mapk12|Birc3|Prkcd|Nlrp6|Nlrp3|Na
0.002511886	3.4	8	Cyp11b2|Hsd3b5|Cyp17a1|Hsd17b1|Akr1d1|Ugt2b17|Cyp
0.005011872	3.3	7	Th|Cyp17a1|Mapk13|Mapk12|Socs2|Socs6|Mapk11
0.006309573	2.7	9	Plcg2|Mapk13|Rassf5|Mapk12|Rock1|Cldn15|Ctnna1|Cldn
0.007943282	2.8	8	Dpp4|Col2a1|Pga5|Col15a1|Col8a1|Col9a1|Col6a3|Col24a
5.01187E-05	5.9	8	Calca|Gipr|Ramp3|Crhr1|Gnb3|Crhr2|Gng4|Gng13
0.000316228	2.3	22	Calca|Sst|Gipr|Adra2a|Gnrh1|Cck|Hrh2|Rln1|Htr2b|P2ry2|
0.000316228	6.3	6	Alb|Slc10a2|Slco1b2|Nr1h4|Akr1d1|Hsd3b7
0.000794328	8.9	4	Alb|Slc10a2|Slco1b2|Nr1h4
0.001	5.1	6	Mapk12|Birc3|Nlrp3|Pstpip1|Nlrp1a|Mapk11
0.001	1.9	27	Calca|Sst|Gipr|Plcb4|Adra2a|Gnrh1|Cck|Hrh2|Rln1|Rgs4|H
0.001258925	6.1	5	Adra2a|Gnb3|Gng4|Rgs17|Gng13
0.001258925	2.7	13	Plcb4|Gnrh1|Cck|Rgs4|Htr2b|P2ry2|Gnb3|Gng4|Prkcd|Prok
0.001584893	12	3	Nr1h4|Akr1d1|Hsd3b7
0.001995262	2.8	11	Camk2d|Trpv2|Atp2b4|Trdn|Clcnka|Clcnkb|Trpm8|Atp10b
0.002511886	11	3	Dpp4|Gnb3|Gng13
0.002511886	6.8	4	Mapk12|Ctnna1|Mef2c|Mapk11
0.003162278	3.3	8	Klrk1|Cd247|Col2a1|Itgb7|Cd40|Pilrb2|Cd226|Siglec1
0.003162278	6.5	4	Adra2a|Gnb3|Gng4|Gng13
0.003162278	9.7	3	Dpp4|Gnb3|Gng13
0.003162278	4.9	5	Col2a1|Lamc2|Loxl2|Col15a1|Col8a1
0.003162278	1.7	28	Alb|Camk2d|Trpv2|Slc7a3|Atp2b4|Slco1b2|Trdn|Gnb3|Ireb

0.003981072	3.9	6	Col2a1|Lamc2|Loxl2|Col15a1|Col8a1|P4ha3
0.005011872	8.2	3	Nlrp3|Pstpip1|Nlrp1a
0.006309573	2.9	8	Alb|Cyp17a1|Hsd17b1|Slc10a2|Slco1b2|Nr1h4|Akr1d1|Hs
0.007943282	3.1	7	Trpv2|Trdn|Clcnka|Clcnkb|Trpm8|Best3|Ano2
0.007943282	7.1	3	Nr1h4|Akr1d1|Hsd3b7
0.007943282	7.1	3	Birc3|Cd40|Ltb
0.007943282	4.9	4	Gipr|Gnb3|Gng4|Gng13
0.007943282	2.1	14	Sst|Plcb4|Adra2a|Rgs4|Cnr2|Gnb3|Cxcl6|Gng4|Prkcd|Fpr1|

2|Shox2|Chrna4|Scn5a|Glra1|Rgs4|Atp2b4|Scn11a|Cacna1g|Sema3a|Runx1|Pclo|Crhr1|Crhr2|Rock1|Nrg1|S

2|Runx1|Prg2|Twist2|Cd14|Nr1h4|Cxcl6|Crhr2|Pou2f2|Flt3|Cd40|Nlrp6|Nlrp3|Osm|Clnk|Ermap|Gbp1|Mnda

2|Cd14|Nr1h4|Crhr2|Pou2f2|Flt3|Cd40|Nlrp3|Osm|Clnk|Mnda|Tlr6|Cd226|Ccm2l|Nlrp1a|Ltb|Postn|Oas2|M
Gabrq|Gabre|Chrna1|Tpbg|Otof|Plg|Lin7a|Nrg1|Stx1a|Cabp1|Camk2n1|Cbln2|Ssh1|Farp1|Mef2c|Cplx3

nur1|Clcnka|Clcnkb|Slc5a5|Prkcd|Ust5r|LOC292543|Sfxn2|Slc7a12|Pla2g2d|Rtbdn|Slc16a12|Slc24a5|Best3

3|Scn11a|Cacna1g|Pclo|Cnr2|Trdn|Il1rn|Kcnip1|Gabrq|Gabre|Chrna1|Stx1a|Kcnh6|Grip2|Cux2|Ssh1|Mef2c

Scn5a|Plcg2|Trpv2|Rgs4|P2ry2|Scn11a|Cacna1g|Ramp3|Crhr1|Trdn|Nr1h4|Il1rn|Crhr2|Kcnip1|Gabre|Clcnka

n2|Chrna4|Rln1|Glra1|Htr2b|P2ry2|Cnr2|Crhr1|Npffr1|Crhr2|Gabrq|Gabre|Nmur1|Chrna1|Plg|Fpr1

tr2b|P2ry2|Ramp3|Cnr2|Crhr1|Gnb3|Cxcl6|Crhr2|Rock1|Gng4|Prkcd|Prok1|Fpr1|Rgs17|Ccl9|Cxcl13|Gng13

o2|Clcnka|Clcnkb|Plg|Gng4|Slc5a5|Steap3|Trpm8|Psmb11|Slc39a6|Cybrd1|Atp10b|Slc24a5|Best3|Atp6v1c2

a|Tlr6|Cd226|Tsku|Ccm2l|Il36rn|Nlrp1a|Ltb|Postn|Oas2|Mpl|Lrrc19|Mapk11|Lilra5
