# Supplementary material for: Transcriptomic Profiling of Electroacupuncture Regulating the Molecular Network in Hippocampus of Rats with Cerebral Ischemia-Reperfusion Injury
Source: Evid Based Complement Alternat Med. 2022 Sep 2;2022:6053106. doi: 10.1155/2022/6053106 (PMC9463016; doi:10.1155/2022/6053106)
Supplement: Supplementary Materials — Table S1: differentially expressed genes of Model/Sham group; Table S2: preliminary enrichment results of Model/Sham group; Table S3: differentially expressed genes of EA/Model group; Table S4: upregulated gene analysis; Table S5: downregulated gene analysis; Table S6: all gene analysis. [file 6053106.f1.zip › Table S6.pdf]

Table S6 All Gene Analysis

Category	Term
GO Biological Processes	GO:0006935
GO Biological Processes	GO:0042330
GO Biological Processes	GO:0034330
GO Biological Processes	GO:0044057
GO Biological Processes	GO:0048812
GO Biological Processes	GO:0007155
GO Biological Processes	GO:0061564
GO Biological Processes	GO:0120039
GO Biological Processes	GO:0048858
GO Biological Processes	GO:0031175
GO Biological Processes	GO:0007411
GO Biological Processes	GO:0000902
GO Biological Processes	GO:0097485
GO Biological Processes	GO:0032989
GO Biological Processes	GO:0051963
GO Biological Processes	GO:0007423
GO Biological Processes	GO:0099536
GO Biological Processes	GO:0000904
GO Biological Processes	GO:0032990
GO Biological Processes	GO:0043583
GO Biological Processes	GO:0007409
GO Biological Processes	GO:0001817
GO Biological Processes	GO:0099537
GO Biological Processes	GO:0048839
GO Biological Processes	GO:0048667
GO Biological Processes	GO:0043269
GO Biological Processes	GO:0050808
GO Biological Processes	GO:0042391
GO Biological Processes	GO:1901888
GO Biological Processes	GO:0050890
GO Biological Processes	GO:0043408
GO Biological Processes	GO:0001819
GO Biological Processes	GO:0050673
GO Biological Processes	GO:0043410
GO Biological Processes	GO:0050919
GO Biological Processes	GO:0098916
GO Biological Processes	GO:0007268
GO Biological Processes	GO:0006820
GO Biological Processes	GO:0001508
GO Biological Processes	GO:0021536
GO Biological Processes	GO:0043524
KEGG Pathway	rno04080
KEGG Pathway	rno04750
KEGG Pathway	rno04360
KEGG Pathway	rno05033
KEGG Pathway	rno04020
KEGG Pathway	rno04713
KEGG Pathway	rno04723
KEGG Pathway	rno04724
KEGG Pathway	rno04670
KEGG Pathway	rno04728
Reactome Gene Sets	R-RNO-112316
Reactome Gene Sets	R-RNO-112314
Reactome Gene Sets	R-RNO-112315

Reactome Gene Sets	R-RNO-6794362
Reactome Gene Sets	R-RNO-373080
Reactome Gene Sets	R-RNO-382551
Reactome Gene Sets	R-RNO-416476
Reactome Gene Sets	R-RNO-500792
Reactome Gene Sets	R-RNO-418597
Reactome Gene Sets	R-RNO-629597
Reactome Gene Sets	R-RNO-400042
Reactome Gene Sets	R-RNO-438066
Reactome Gene Sets	R-RNO-372790
Reactome Gene Sets	R-RNO-5576892
Reactome Gene Sets	R-RNO-388844
Reactome Gene Sets	R-RNO-1474290
Reactome Gene Sets	R-RNO-194068
Reactome Gene Sets	R-RNO-5576891
Reactome Gene Sets	R-RNO-159418
Reactome Gene Sets	R-RNO-422475
Reactome Gene Sets	R-RNO-9675108
Reactome Gene Sets	R-RNO-2022090
Reactome Gene Sets	R-RNO-622323
Reactome Gene Sets	R-RNO-193807
Reactome Gene Sets	R-RNO-390666
Reactome Gene Sets	R-RNO-629594
Reactome Gene Sets	R-RNO-381676
Reactome Gene Sets	R-RNO-198933

**Description**	**Pvalue**
chemotaxis	5.01187E-08
taxis	6.30957E-08
cell junction organization	6.30957E-08
regulation of system process	7.94328E-08
neuron projection morphogenesis	0.0000001
cell adhesion	0.0000001
axon development	1.25893E-07
plasma membrane bounded cell projection morphogenesis	1.25893E-07
cell projection morphogenesis	1.99526E-07
neuron projection development	2.51189E-07
axon guidance	3.16228E-07
cell morphogenesis	3.16228E-07
neuron projection guidance	3.16228E-07
cellular component morphogenesis	3.16228E-07
regulation of synapse assembly	5.01187E-07
sensory organ development	5.01187E-07
synaptic signaling	6.30957E-07
cell morphogenesis involved in differentiation	6.30957E-07
cell part morphogenesis	0.000001
ear development	1.25893E-06
axonogenesis	1.25893E-06
regulation of cytokine production	1.58489E-06
trans-synaptic signaling	1.99526E-06
inner ear development	2.51189E-06
cell morphogenesis involved in neuron differentiation	3.16228E-06
regulation of ion transport	5.01187E-06
synapse organization	7.94328E-06
regulation of membrane potential	7.94328E-06
regulation of cell junction assembly	7.94328E-06
cognition	1.58489E-05
regulation of MAPK cascade	1.58489E-05
positive regulation of cytokine production	1.58489E-05
epithelial cell proliferation	1.58489E-05
positive regulation of MAPK cascade	1.58489E-05
negative chemotaxis	1.99526E-05
anterograde trans-synaptic signaling	1.99526E-05
chemical synaptic transmission	1.99526E-05
anion transport	1.99526E-05
action potential	2.51189E-05
diencephalon development	0.000794328
negative regulation of neuron apoptotic process	0.003162278
Neuroactive ligand-receptor interaction	3.98107E-06
Inflammatory mediator regulation of TRP channels	5.01187E-05
Axon guidance	0.000251189
Nicotine addiction	0.000630957
Calcium signaling pathway	0.000794328
Circadian entrainment	0.001
Retrograde endocannabinoid signaling	0.003162278
Glutamatergic synapse	0.003981072
Leukocyte transendothelial migration	0.005011872
Dopaminergic synapse	0.006309573
Neuronal System	0.000001
Neurotransmitter receptors and postsynaptic signal transmission	5.01187E-05
Transmission across Chemical Synapses	6.30957E-05

Protein-protein interactions at synapses	0.0001
Class B/2 (Secretin family receptors)	0.000501187
Transport of small molecules	0.000794328
G alpha (q) signalling events	0.001
GPCR ligand binding	0.001995262
G alpha (z) signalling events	0.002511886
Highly calcium permeable nicotinic acetylcholine receptors	0.003981072
Adrenaline,noradrenaline inhibits insulin secretion	0.003981072
Unblocking of NMDA receptors, glutamate binding and activation	0.003981072
Signaling by GPCR	0.003981072
Phase 0 - rapid depolarisation	0.003981072
Receptor-type tyrosine-protein phosphatases	0.005011872
Collagen formation	0.005011872
Bile acid and bile salt metabolism	0.006309573
Cardiac conduction	0.006309573
Recycling of bile acids and salts	0.006309573
Axon guidance	0.006309573
Nervous system development	0.007943282
Assembly of collagen fibrils and other multimeric structures	0.007943282
Presynaptic nicotinic acetylcholine receptors	0.007943282
Synthesis of bile acids and bile salts via 27-hydroxycholesterol	0.007943282
Serotonin receptors	0.007943282
Highly calcium permeable postsynaptic nicotinic acetylcholine receptors	0.007943282
Glucagon-like Peptide-1 (GLP1) regulates insulin secretion	0.01
Immunoregulatory interactions between a Lymphoid and a non-Lymphoid cell	0.01

<table>
<tr><th>Enrichment</th><th>Counts</th><th>Genes</th></tr>
<tr><td>2.2</td><td>53</td><td>Calca|Pdgfb|Scg2|Dpp4|Smo|Dcc|Nog|Nkx2-1|Epha6|Gap43|Sema3a|C</td></tr>
<tr><td>2.2</td><td>53</td><td>Calca|Pdgfb|Scg2|Dpp4|Smo|Dcc|Nog|Nkx2-1|Epha6|Gap43|Sema3a|C</td></tr>
<tr><td>2.1</td><td>56</td><td>Grin2a|Grm5|Htr1a|Pdgfb|Pmp22|Gabrb3|Il1rap|Slc1a1|Itgb4|Cdh7|Ug</td></tr>
<tr><td>2</td><td>65</td><td>Adra1b|Calca|Camk2d|Grin2a|Grm1|Htr1a|Igf1|Nppa|Pdgfb|Tf|Thrb|Ac</td></tr>
<tr><td>2.2</td><td>54</td><td>Pmp22|Smo|Cnp|Cck|Dcc|Nog|Nkx2-1|Ddr1|Epha6|Gap43|Erbb3|Sema</td></tr>
<tr><td>1.9</td><td>74</td><td>Calca|Mog|Pmp22|Ret|Dpp4|Dcc|Il1rap|Ddr1|Itgb7|Itgb4|Cdh7|Mag|Lg</td></tr>
<tr><td>2.3</td><td>47</td><td>Pmp22|Plp1|Apod|Smo|Cnp|Cck|Dcc|Nog|Nkx2-1|Ddr1|Epha6|Mag|Ga</td></tr>
<tr><td>2.1</td><td>54</td><td>Pmp22|Smo|Cnp|Cck|Dcc|Nog|Nkx2-1|Ddr1|Epha6|Gap43|Erbb3|Sema</td></tr>
<tr><td>2.1</td><td>54</td><td>Pmp22|Smo|Cnp|Cck|Dcc|Nog|Nkx2-1|Ddr1|Epha6|Gap43|Erbb3|Sema</td></tr>
<tr><td>1.9</td><td>70</td><td>Pmp22|Plp1|Apod|Smo|Cnp|Cck|Dcc|Nog|Nkx2-1|Ddr1|Epha6|Mag|Ga</td></tr>
<tr><td>2.7</td><td>32</td><td>Smo|Dcc|Nog|Nkx2-1|Epha6|Gap43|Sema3a|Nr4a3|Epha8|Slit3|Efna2|</td></tr>
<tr><td>1.9</td><td>71</td><td>Pmp22|Smo|Cnp|Cck|Dcc|Hrh2|Nog|Nkx2-1|Ddr1|Itgb7|Cdh7|Epha6|G</td></tr>
<tr><td>2.7</td><td>32</td><td>Smo|Dcc|Nog|Nkx2-1|Epha6|Gap43|Sema3a|Nr4a3|Epha8|Slit3|Efna2|</td></tr>
<tr><td>2</td><td>62</td><td>Pmp22|Smo|Cnp|Cck|Dcc|Nog|Nkx2-1|Ddr1|Itgb4|Epha6|Mag|Gap43|E</td></tr>
<tr><td>3.5</td><td>21</td><td>Il1rap|Tpbg|Ghsr|Efna5|Epha7|Clstn2|Cux2|Cbln2|Dkk1|Lrrtm3|Il1rapl</td></tr>
<tr><td>1.9</td><td>64</td><td>Ascl2|Crygd|Gfi1|Igf1|Pdgfb|Ret|Thrb|Gabrb3|Th|Col2a1|Mip|Nog|Ocm</td></tr>
<tr><td>2.2</td><td>46</td><td>Grin2a|Grm1|Grm5|Htr1a|Gabrb3|Th|Chrna5|Htr4|Hrh2|Il1rap|Chrna4|</td></tr>
<tr><td>2</td><td>58</td><td>Pmp22|Smo|Cnp|Cck|Dcc|Hrh2|Nog|Nkx2-1|Itgb7|Epha6|Gap43|Erbb3</td></tr>
<tr><td>2</td><td>54</td><td>Pmp22|Smo|Cnp|Cck|Dcc|Nog|Nkx2-1|Ddr1|Epha6|Gap43|Erbb3|Sema</td></tr>
<tr><td>2.5</td><td>33</td><td>Gfi1|Igf1|Gabrb3|Col2a1|Nog|Ocm|Ddr1|Cebpd|Hpca|Kcnk3|Gabra5|N</td></tr>
<tr><td>2.2</td><td>41</td><td>Pmp22|Smo|Cnp|Cck|Dcc|Nog|Nkx2-1|Epha6|Gap43|Erbb3|Sema3a|Nr</td></tr>
<tr><td>1.8</td><td>68</td><td>Calca|Cebpb|Egr1|Igf1|Lpl|Mog|Klrk1|Adra2a|Fgfr4|Apod|Il1rap|Arg1|</td></tr>
<tr><td>2.2</td><td>42</td><td>Grin2a|Grm1|Grm5|Htr1a|Gabrb3|Th|Chrna5|Htr4|Hrh2|Il1rap|Chrna4|</td></tr>
<tr><td>2.5</td><td>30</td><td>Gfi1|Igf1|Gabrb3|Col2a1|Ocm|Cebpd|Hpca|Kcnk3|Gabra5|Nr4a3|Dll1|</td></tr>
<tr><td>2</td><td>47</td><td>Pmp22|Smo|Cnp|Cck|Dcc|Nog|Nkx2-1|Epha6|Gap43|Erbb3|Sema3a|Nr</td></tr>
<tr><td>1.8</td><td>66</td><td>Calca|Camk2d|Cebpb|Grin2a|Grm1|Grm5|Htr1a|Igf1|Nppa|Pdgfb|Tf|Pl</td></tr>
<tr><td>2.2</td><td>36</td><td>Grin2a|Grm5|Htr1a|Pdgfb|Pmp22|Gabrb3|Il1rap|Slc1a1|Pclo|Cttn|Sncg</td></tr>
<tr><td>2</td><td>47</td><td>Camk2d|Grin2a|Grm1|Grm5|Nppa|Pmp22|Gabrb3|Chrna5|Cck|Chrna4|</td></tr>
<tr><td>2.5</td><td>28</td><td>Apod|Il1rap|Cldn1|Rock1|Tpbg|Ghsr|Efna5|Epha7|Clstn2|Nrp1|Pkp2|C</td></tr>
<tr><td>2.1</td><td>39</td><td>Adra1b|Cebpb|Egr1|Grin2a|Grm5|Igf1|Gabrb3|Th|Cck|Htr4|Hrh2|Nog|</td></tr>
<tr><td>1.7</td><td>62</td><td>Adra1b|Camk2d|Grm1|Grm5|Igf1|Nppa|Pdgfb|Ret|Tgfa|Adra2a|Fgfr4|D</td></tr>
<tr><td>1.9</td><td>47</td><td>Calca|Cebpb|Egr1|Lpl|Klrk1|Adra2a|Il1rap|Plcg2|Rasgrp1|Mapk13|Htr</td></tr>
<tr><td>3.2</td><td>17</td><td>Calca|Cebpb|Pdgfb|Tgfa|Notch2|Nr1h4|Kit|Olr59|Fap|Loxl2|Lgr5|Bmp</td></tr>
<tr><td>1.9</td><td>49</td><td>Adra1b|Camk2d|Grm1|Grm5|Igf1|Pdgfb|Ret|Tgfa|Adra2a|Fgfr4|Dcc|Ig</td></tr>
<tr><td>4.5</td><td>11</td><td>Dpp4|Sema3a|Slit3|Nrg1|Efna5|Epha7|Sema3d|Sema3e|Sema5a|Unc5c</td></tr>
<tr><td>2.1</td><td>38</td><td>Grin2a|Grm1|Grm5|Htr1a|Gabrb3|Th|Chrna5|Htr4|Hrh2|Chrna4|Glra1|</td></tr>
<tr><td>2.1</td><td>38</td><td>Grin2a|Grm1|Grm5|Htr1a|Gabrb3|Th|Chrna5|Htr4|Hrh2|Chrna4|Glra1|</td></tr>
<tr><td>2</td><td>43</td><td>Grm1|Abcb1b|Slc9a2|Slc9a4|Gabrb3|Mip|Slc1a1|Glra1|Slc7a3|Slc10a2</td></tr>
<tr><td>3.3</td><td>16</td><td>Grin2a|Pmp22|Chrna4|Scn5a|Glra1|Scn4a|Scn11a|Cacna1g|Gjd2|Gria1</td></tr>
<tr><td>2.7</td><td>13</td><td>Pcsk1|Smo|Nog|Nkx2-1|Pou3f2|Sema3a|Crhr1|Crhr2|Msx1|Lhx3|Nrp1|</td></tr>
<tr><td>2</td><td>19</td><td>Cebpb|Gabrb3|Smo|Slc1a1|Mag|Erbb3|Gabra5|Ptk2b|Grik2|Nr4a3|Roc</td></tr>
<tr><td>2.2</td><td>40</td><td>Adra1b|Calca|Grin2a|Grm1|Grm5|Htr1a|Sst|Thrb|Gabrb3|Gipr|Adra2a|</td></tr>
<tr><td>3</td><td>17</td><td>Camk2d|Igf1|Plcb4|Il1rap|Plcg2|Trpv2|Mapk13|Htr2b|P2ry2|Mapk12|P</td></tr>
<tr><td>2.4</td><td>21</td><td>Camk2d|Smo|Dcc|Epha6|Plcg2|Sema3a|Epha8|Rock1|Slit3|Efna2|Efna</td></tr>
<tr><td>4.1</td><td>8</td><td>Grin2a|Gabrb3|Chrna4|Gria3|Gabra5|Gria1|Gabrq|Gabre</td></tr>
<tr><td>2</td><td>24</td><td>Adra1b|Camk2d|Grin2a|Grm1|Grm5|Pdgfb|Ret|Plcb4|Fgfr4|Htr4|Hrh2|</td></tr>
<tr><td>2.7</td><td>13</td><td>Camk2d|Grin2a|Plcb4|Gria3|Cacna1g|Gria1|Gnb3|Gng4|Cacna1h|Ryr3</td></tr>
<tr><td>2.1</td><td>16</td><td>Grm1|Grm5|Gabrb3|Plcb4|Mapk13|Gria3|Gabra5|Gria1|Mapk12|Gnb3|</td></tr>
<tr><td>2.3</td><td>13</td><td>Grin2a|Grm1|Grm5|Plcb4|Slc1a1|Homer3|Gria3|Gria1|Grik2|Gnb3|Gn</td></tr>
<tr><td>2.3</td><td>13</td><td>Plcg2|Mapk13|Ptk2b|Rassf5|Mapk12|Cldn1|Rock1|Cldn11|Vav3|Cldn1</td></tr>
<tr><td>2.1</td><td>14</td><td>Camk2d|Grin2a|Plcb4|Th|Mapk13|Gria3|Gria1|Mapk12|Gnb3|Gng4|Gr</td></tr>
<tr><td>2.5</td><td>35</td><td>Camk2d|Grin2a|Grm1|Grm5|Gabrb3|Il1rap|Slc1a1|Chrna4|Glra1|Home</td></tr>
<tr><td>3</td><td>17</td><td>Camk2d|Grin2a|Gabrb3|Chrna4|Glra1|Gria3|Gabra5|Gria1|Grik2|Gnb3</td></tr>
<tr><td>2.5</td><td>22</td><td>Camk2d|Grin2a|Gabrb3|Slc1a1|Chrna4|Glra1|Gria3|Gabra5|Gria1|Grik</td></tr>
</table>

3.9	11	Grin2a|Grm1|Grm5|Il1rap|Homer3|Gria3|Gria1|Ppfia4|Lrrtm3|Il1rapl2|
3.8	9	Calca|Gipr|Ramp3|Crhr1|Gnb3|Crhr2|Gng4|Gnb4|Gng13
1.6	46	Alb|Camk2d|Lpl|Slc9a2|Slc9a4|Tf|Apod|Slc1a1|Trpv2|Slc7a3|Atp2b4|S
2.2	19	Grm1|Grm5|Plcb4|Gnrh1|Cck|Rgs4|Htr2b|P2ry2|Rgs5|Gnb3|Ghsr|Gng4
1.8	30	Calca|Grm1|Grm5|Htr1a|Sst|Gipr|Adra2a|Gnrh1|Cck|Htr4|Hrh2|Rln1|H
4.2	6	Adra2a|Gnb3|Gng4|Gnb4|Rgs17|Gng13
8.7	3	Chrna4|Chrna1|Chrna2
4.6	5	Adra2a|Gnb3|Gng4|Gnb4|Gng13
4.6	5	Camk2d|Grin2a|Gria3|Gria1|Nefl
1.6	39	Calca|Grm1|Grm5|Htr1a|Sst|Gipr|Plcb4|Adra2a|Gnrh1|Cck|Htr4|Hrh2|F
3.8	6	Camk2d|Scn5a|Scn4a|Scn11a|Cacng8|Scn3b
5.4	4	Il1rap|Ppfia4|Il1rapl2|Slitrk2
2.9	8	Col2a1|Itgb4|Lamc2|Loxl2|Col15a1|Col8a1|Adamts3|P4ha3
3.6	6	Alb|Slc10a2|Slco1b2|Nr1h4|Akr1d1|Hsd3b7
2.3	12	Camk2d|Nppa|Scn5a|Scn4a|Kcnk3|Atp2b4|Scn11a|Kcnip2|Trdn|Kcnip
5.1	4	Alb|Slc10a2|Slco1b2|Nr1h4
1.9	19	Ret|Gfra2|Dcc|Col2a1|Gap43|Sema3a|Mapk12|Rock1|Efna2|Efna5|Eph
1.9	19	Ret|Gfra2|Dcc|Col2a1|Gap43|Sema3a|Mapk12|Rock1|Efna2|Efna5|Eph
3.4	6	Col2a1|Itgb4|Lamc2|Loxl2|Col15a1|Col8a1
6.8	3	Chrna4|Chrna1|Chrna2
6.8	3	Nr1h4|Akr1d1|Hsd3b7
6.8	3	Htr1a|Htr4|Htr2b
6.8	3	Chrna4|Chrna1|Chrna2
3.8	5	Gnb3|Gng4|Gnb4|Kcng2|Gng13
2.4	10	Klrk1|Cd247|Col2a1|Itgb7|Cd40|Pilrb2|Cd226|Siglec1|Icam5|Cd3e

xcr1|Cnr2|Nr4a3|Epha8|Cxcl6|Kit|Slit3|Tpbg|Efna2|Lyst|Nrg1|Efna5|Il16|Prkcd|Lhx3|Epha7|Lamc2|Hsd3b
xcr1|Cnr2|Nr4a3|Epha8|Cxcl6|Kit|Slit3|Tpbg|Efna2|Lyst|Nrg1|Efna5|Il16|Prkcd|Lhx3|Epha7|Lamc2|Hsd3b
t8|Ptk2b|Pclo|Nr1h4|Cttn|Sncg|Cldn1|Chrna1|Ceacam1|Nefl|Cldn11|Nrg1|Efna5|Ppfia4|Cabp1|Nrp1|Pkp2|C
dra2a|Th|Fgfr4|Cck|Hrh2|Shox2|Slc1a1|Chrna4|Scn5a|Glra1|Scn4a|Mag|Rgs4|Homer3|Atp2b4|Scn11a|Cacn
13a|Ugt8|Nr4a3|Cttn|Epha8|Rock1|Slit3|Nefl|Tpbg|Efna2|Efna5|Lhx3|Epha7|Lamc2|Sema3d|Nrp1|Lgi1|Lhx
als7|Ccn5|Ptk2b|Acan|Milr1|Ninj2|Tcam1|Cttn|Epha8|Kit|Cldn1|Mcam|Mia|Ceacam1|Rock1|Mybph|Fbn1|
p43|Erbb3|Sema3a|Nr4a3|Epha8|Slit3|Nefl|Efna2|Efna5|Lhx3|Epha7|Lamc2|Sema3d|Nrp1|Lgi1|Lhx9|Mycl
13a|Ugt8|Nr4a3|Cttn|Epha8|Rock1|Slit3|Nefl|Tpbg|Efna2|Efna5|Lhx3|Epha7|Lamc2|Sema3d|Nrp1|Lgi1|Lhx
13a|Ugt8|Nr4a3|Cttn|Epha8|Rock1|Slit3|Nefl|Tpbg|Efna2|Efna5|Lhx3|Epha7|Lamc2|Sema3d|Nrp1|Lgi1|Lhx
p43|Erbb3|Sema3a|Ugt8|Ptk2b|Nr4a3|Cttn|Epha8|Rock1|Slit3|Nefl|Cit|Tpbg|Efna2|Efna5|Gpr37|Hey1|Lhx
Efna5|Lhx3|Epha7|Lamc2|Sema3d|Nrp1|Lgi1|Lhx9|Mycbp2|Neurog2|Sema3e|Wnt3a|Gli2|Sema5a|Ephb2|E
ap43|Erbb3|Sema3a|Ugt8|Nr4a3|Cttn|Epha8|Msx1|Rock1|Slit3|Nefl|Tpbg|Efna2|Nrg1|Efna5|Lhx3|Epha7|L
Efna5|Lhx3|Epha7|Lamc2|Sema3d|Nrp1|Lgi1|Lhx9|Mycbp2|Neurog2|Sema3e|Wnt3a|Gli2|Sema5a|Ephb2|E
rbb3|Sema3a|Ugt8|Nr4a3|Cttn|Epha8|Rock1|Slit3|Nefl|Tpbg|Chn2|Efna2|Efna5|Lhx3|Epha7|Lamc2|Sema3

n|Slc1a1|Ddr1|Cebpd|Hpca|Notch2|Erbb3|Kcnk3|Atp2b4|Gabra5|Nr4a3|Twist2|Kit|Msx1|Fbn1|Dll1|Efna2|
Glra1|Pdyn|Htr2b|Gabra5|Cacna1g|Gjd2|Gria1|Grik2|Rgs10|Pclo|Sncg|Doc2a|Gabrq|Gabre|Chrna1|Tpbg|O
|Sema3a|Nr4a3|Epha8|Rock1|Slit3|Tpbg|Efna2|Efna5|Lhx3|Epha7|Lamc2|Sema3d|Nrp1|Lgi1|Lhx9|Mycbp
13a|Ugt8|Nr4a3|Cttn|Epha8|Rock1|Slit3|Nefl|Tpbg|Efna2|Efna5|Lhx3|Epha7|Lamc2|Sema3d|Nrp1|Lgi1|Lhx
r4a3|Msx1|Dll1|Efna2|Otof|Lin7a|Hey1|Lhx3|Plppr4|Lgr5|Bmper|Wnt3a|Gli2|Mnda|Prox1|Ror2|Tsku|Fat4|
4a3|Epha8|Slit3|Efna2|Efna5|Lhx3|Epha7|Lamc2|Sema3d|Nrp1|Lgi1|Lhx9|Mycbp2|Plppr4|Neurog2|Sema3
Plcg2|Rasgrp1|Mapk13|Lgals7|Homer3|Htr2b|P2ry2|Runx1|Crebbp|Prg2|Nr4a3|Twist2|Cd14|Nr1h4|Cxcl6|
Glra1|Pdyn|Htr2b|Gabra5|Cacna1g|Gjd2|Gria1|Grik2|Pclo|Sncg|Doc2a|Gabrq|Gabre|Chrna1|Tpbg|Otof|Plg|
Efna2|Otof|Lin7a|Hey1|Lhx3|Plppr4|Lgr5|Bmper|Wnt3a|Gli2|Mnda|Prox1|Ror2|Tsku|Fat4|Atp6v1b1|Ephb
4a3|Epha8|Slit3|Tpbg|Efna2|Efna5|Lhx3|Epha7|Lamc2|Sema3d|Nrp1|Lgi1|Lhx9|Mycbp2|Plppr4|Neurog2|S
p1|Plcb4|Adra2a|Fgfr4|Cck|Hrh2|Chrna4|Scn5a|Scn4a|Hpca|Arg1|Plcg2|Trpv2|Rgs4|Homer3|P2ry2|Scn11a
|Chrna1|Nefl|Nrg1|Efna5|Ppfia4|Cabp1|Nrp1|Cbln2|Lrrtm3|Sema3e|Igsf21|Adgrb3|Dock10|Igfn1|Farp1|Kl
Scn5a|Nr3c2|Glra1|Scn4a|Rgs4|Kcnk3|Gria3|Scn11a|Gabra5|Cacna1g|Gjd2|Gria1|Ptk2b|Grik2|Pclo|Kcnip
ux2|Cbln2|Rhod|Dkk1|Lrrtm3|Il1rapl2|Adgrb3|Wnt3a|Farp1|Slitrk2|Ephb2|Gpc4|Fzd5|Flrt3|Mef2c|Lrrn1|V
lc1a1|Chrna4|Gabra5|Kcnab1|Gria1|Crebbp|Crhr1|Il1rn|Kit|Arl6ip5|Tpbg|Ghsr|Nrg1|Cux2|Dkk1|Nts|Aph1
cc|Igfbp6|Plcg2|Rasgrp1|Notch2|Htr2b|Ptk2b|Grik2|Ramp3|Il1rn|Dusp4|Epha8|Kit|Crhr2|Arl6ip5|Ceacam
2b|P2ry2|Runx1|Prg2|Nr4a3|Cd14|Nr1h4|Kit|Cd55|Crhr2|Scamp5|Twist1|Il16|Pou2f2|Flt3|Cd40|Nlrp3|Dhx

fbp6|Plcg2|Rasgrp1|Notch2|Htr2b|Ptk2b|Ramp3|Il1rn|Epha8|Kit|Crhr2|Arl6ip5|Ceacam1|Rock1|Tpbg|Nrg1

Pdyn|Htr2b|Gabra5|Cacna1g|Gjd2|Gria1|Grik2|Pclo|Sncg|Doc2a|Gabrq|Gabre|Chrna1|Tpbg|Otof|Lin7a|Stx
Pdyn|Htr2b|Gabra5|Cacna1g|Gjd2|Gria1|Grik2|Pclo|Sncg|Doc2a|Gabrq|Gabre|Chrna1|Tpbg|Otof|Lin7a|Stx
|Gabra5|Slco1b2|Nr1h4|Gabrq|Gabre|Nmur1|Arl6ip5|Clcnka|Clcnkb|Ceacam1|Slc6a20|Slc5a5|Prkcd|Ust5

Chrna5|Mas1|Gnrh1|Cck|Htr4|Hrh2|Chrna4|Rln1|Glra1|Pdyn|Htr2b|P2ry2|Gria3|Gabra5|Gria1|Grik2|Cnr2|

r3|Kcnk3|Gria3|Gabra5|Kcnab1|Gjd2|Gria1|Grik2|Gnb3|Kcns2|Arl6ip5|Chrna1|Nefl|Lin7a|Gng4|Stx1a|Kc

Slco1b2|Trdn|Gnb3|Ireb2|Clcnka|Clcnkb|Soat1|Plg|Slc6a20|Gng4|Slc5a5|Steap3|Trpm8|Slc16a2|Slc30a10|[illegible]

tr2b|P2ry2|Cxcr1|Ramp3|Cnr2|Crhr1|Gnb3|Cxcl6|Crhr2|Gng4|Gpr37|Prok1|Fpr1|Gnb4|Ccl9|Cxcl13|Gng13

Rln1|Rgs4|Htr2b|P2ry2|Cxcr1|Rgs5|Ramp3|Cnr2|Crhr1|Gnb3|Cxcl6|Crhr2|Rock1|Ghsr|Gng4|Gpr37|Prkcd|P

7|Sema3d|Nrp1|Lgi1|Ccl24|Lhx9|Mycbp2|Vav3|Neurog2|Sema3e|Wnt3a|Gli2|Sema5a|Ephb2|Bcl11b|Ccl9|
7|Sema3d|Nrp1|Lgi1|Ccl24|Lhx9|Mycbp2|Vav3|Neurog2|Sema3e|Wnt3a|Gli2|Sema5a|Ephb2|Bcl11b|Ccl9|
bln2|Rhod|Lrrtm3|Sema3e|Igsf21|Adgrb3|Dock10|Cldn15|Igfn1|Farp1|Dsp|Mpp7|Ctnna1|Kifc3|Klk8|Slitrk
a1g|Sema3a|Ptk2b|Runx1|Pclo|Nr4a3|Crhr1|Cttn|Kit|Crhr2|Rock1|Ghsr|Foxo1|Nrg1|Cacna1h|Stx1a|Atp2a
9|Mycbp2|Plppr4|Neurog2|Sema3e|Dock10|Wnt3a|Gli2|Farp1|Klk8|Tsku|Slitrk2|Tet1|Sema5a|Ephb2|Bcl1
Cldn11|Nrg1|Efna5|Clstn2|Epcam|Fap|Nrp1|Pcdhga11|Pkp2|Lyve1|Rhod|Tecta|Cntnap5b|Wnt3a|Cldn15|Ig
p2|Plppr4|Neurog2|Sema3e|Wnt3a|Gli2|Ctnna1|Tsku|Slitrk2|Sema5a|Ephb2|Bcl11b|Zic2|Unc5c|Bhlhe22|
9|Mycbp2|Plppr4|Neurog2|Sema3e|Dock10|Wnt3a|Gli2|Farp1|Klk8|Tsku|Slitrk2|Tet1|Sema5a|Ephb2|Bcl1
9|Mycbp2|Plppr4|Neurog2|Sema3e|Dock10|Wnt3a|Gli2|Farp1|Klk8|Tsku|Slitrk2|Tet1|Sema5a|Ephb2|Bcl1
3|Epha7|Lamc2|Sema3d|Nrp1|Lgi1|Arid1b|Lhx9|Klhl1|Mycbp2|Plppr4|Neurog2|Sema3e|Dock10|Wnt3a|Gl

amc2|Sema3d|Nrp1|Lgi1|Lhx9|Mycbp2|Sipa1l3|Dkk1|Plppr4|Neurog2|Sema3e|Col15a1|Dock10|Wnt3a|Bc

d|Nrp1|Lgi1|Lhx9|Mycbp2|Plppr4|Neurog2|Sema3e|Dock10|Wnt3a|Gli2|Prox1|Farp1|Myom2|Klk8|Tsku|S

Otof|Lin7a|Twist1|Hey1|Lhx3|Nrp1|Plaat1|Hsf4|Sipa1l3|Plppr4|Lgr5|Bmper|Tfap2b|Wnt3a|Fscn2|Col8a1|C
tof|Plg|Lin7a|Nrg1|Stx1a|Chrna2|Cabp1|Clstn2|Arid1b|Camk2n1|Cbln2|Aph1b|Wnt3a|Ssh1|Farp1|Ephb2|S
2|Sipa1l3|Dkk1|Plppr4|Neurog2|Sema3e|Col15a1|Dock10|Wnt3a|Gli2|Prox1|Farp1|Dact2|Tsku|Slitrk2|Tet1
9|Mycbp2|Plppr4|Neurog2|Sema3e|Dock10|Wnt3a|Gli2|Farp1|Klk8|Tsku|Slitrk2|Tet1|Sema5a|Ephb2|Bcl1

e|Wnt3a|Gli2|Tsku|Slitrk2|Sema5a|Ephb2|Bcl11b|Zic2|Unc5c|Bhlhe22|Flrt3|Mef2c|Draxin|Auts2
Kit|Cd55|Crhr2|Scamp5|Ceacam1|Dll1|Ghsr|Twist1|Il16|Pou2f2|Flt3|Cd40|Nlrp6|Nlrp3|Dhx33|Osm|Clnk|E
|Lin7a|Stx1a|Chrna2|Clstn2|Arid1b|Camk2n1|Cbln2|Aph1b|Ssh1|Farp1|Ephb2|Shisa6|Mef2c|Cplx3

ema3e|Dock10|Wnt3a|Gli2|Farp1|Tsku|Slitrk2|Tet1|Sema5a|Ephb2|Bcl11b|Zic2|Unc5c|Bhlhe22|Flrt3|Elav
|Cacna1g|Kcnab1|Ptk2b|Kcnip2|Ramp3|Crhr1|Trdn|Nr1h4|Il1rn|Sncg|Crhr2|Kcnip1|Gabre|Kcns2|Arl6ip5|C

|Cnr2|Trdn|Il1rn|Kcnip1|Gabrq|Gabre|Arl6ip5|Chrna1|Cacna1h|Stx1a|Kcnh6|Chrna2|Grip2|Scn3b|Pkp2|Cu

1|Rock1|Tpbg|Foxo1|Nrg1|Dusp6|Gpr37|Prkcd|Dusp5|Epha7|Cd40|Nlrp6|Prok1|Nrp1|Dhx33|Ccl24|Slc30a1
33|Osm|Clnk|Wnt3a|Tomm70|Mnda|Tlr6|Cd226|Ccm2l|Ephb2|Cd3e|Fzd5|Nlrp1a|Ltb|Postn|Oas2|Clec9a|M

|Gpr37|Prkcd|Cd40|Prok1|Nrp1|Dhx33|Ccl24|Slc30a10|Osm|Tnfrsf19|Dkk1|Sh3rf3|Bmper|Tlr6|Ror2|Denn

|Pcyox1|Slc16a11|Ttyh2|LOC292543|Sfxn2|Slc7a12|Slc35d1|Pla2g2d|Rtbdn|Clca1|Slc16a12|Ano3|Slc24a

Psmb11|Slc39a6|Gnb4|Cybrd1|Slc35d1|Atp10b|Nipal4|Slc45a3|Clca1|Slc24a5|Ahcyl2|Best3|Atp6v1c2|Slc4

1|Kcnh6|Olr59|Dusp5|Grip2|Pde9a|Scn3b|Pkp2|Cux2|Hrc|Tnnc2|Tomm70|Ssh1|Tmem38a|Dsp|Tshz3|Klk8|

fn1|Dsp|Pcdh1|Ctnna1|Kifc3|Slitrk2|Cdh18|Fat4|Fibcd1|Icam5|Tjp3|Itga11|Gpc4|Cadm2|Dgcr2|Pcdh7|Postı

i2|Farp1|Ctnna1|Klk8|Tsku|Slitrk2|Tet1|Sema5a|Fat4|Ephb2|Bcl11b|Cd3e|Mospd4|Zic2|Postn|Unc5c|Blk|B

l6|Ssh1|Gli2|Prox1|Farp1|Dact2|Klk8|Tsku|Slitrk2|Tet1|Cdh18|Sema5a|Arhgef26|Ephb2|Bcl11b|Zic2|Postn

litrk2|Tet1|Sema5a|Ephb2|Bcl11b|Zic2|Postn|Unc5c|Bhlhe22|Flrt3|Elavl4|Ldb3|Mef2c|Pgm5|Dip2a|Draxin

Gli2|Igfn1|Mnda|Prox1|Myom2|Ror2|Tsku|Fat4|Atp6v1b1|Ephb2|Smoc1|Bcl11b|Tgif1|Nhs|Fzd5|Bhlhe22|C

|Sema5a|Arhgef26|Ephb2|Bcl11b|Zic2|Unc5c|Bhlhe22|Flrt3|Mpl|Elavl4|Mef2c|Col24a1|Dip2a|Draxin|Auts

Ermap|Wnt3a|Bcl6|Tomm70|Gbp1|Mnda|Tlr6|Cd226|Tsku|Ccm2l|Il36rn|Ephb2|Cd3e|Fzd5|Nlrp1a|Ltb|Postı

Clcnka|Clcnkb|Twist1|Cacna1h|Atp2a1|Kcnh6|Il16|Cacng8|Cabp1|Prss8|Scn3b|Slc30a10|Hrc|Tmem38a|Kcı

10|Osm|Tnfrsf19|Dkk1|Sh3rf3|Bmper|Gbp1|Ppef2|Tlr6|Dusp26|Ror2|Dennd2b|Sfrp5|Ephb2|Fzd5|Ccl9|Gab

[illegible]|Ptprt|Elmo2|Mybpc1|Flrt3|Mpl|Itgbl1|Otoa|Fyb2|Cldn20|Thbs3|Svep1|Cdhr3|Megf11
